# Ahcy Acts as an Effector of Hnf4a‐Driven Super‐Enhancer Activation to Alleviate MASLD During Intermittent Fasting

**DOI:** 10.1002/advs.76826

**Published:** 2026-07-27

**Authors:** Huafeng Chen, Shilin Zhang, Xiaojie Deng, Wenqiang Xie, Fen Xu, Jie Shen, Hua Liang

**Affiliations:** ^1^ Department of Endocrinology and Metabolism The Eighth Affiliated Hospital Southern Medical University (The First People's Hospital of Shunde, Foshan) Foshan Guangdong China; ^2^ Department of Endocrinology and Metabolism Guangzhou First People's Hospital Guangzhou Guangdong China; ^3^ Department of Endocrinology and Metabolism The Third Affiliated Hospital Sun Yat‐sen University Guangzhou Guangdong China; ^4^ Medical Research Center The Eighth Affiliated Hospital Southern Medical University (The First People's Hospital of Shunde, Foshan) Foshan Guangdong China

**Keywords:** Ahcy, Hnf4a, intermittent fasting, MASLD, super‐enhancer

## Abstract

Intermittent fasting (IF) ameliorates metabolic dysfunction‐associated steatotic liver disease (MASLD), but the underlying mechanism remains unclear. Combined CUT&Run and transcriptomic analysis shows that hepatic Ahcy is controlled by the super‑enhancer (SE) and acts as a key mediator of IF's benefit. Liver‑specific Ahcy knockout worsens high‑fat diet (HFD)‑induced hepatic steatosis and blunts the protective effect of IF. Furthermore, inhibition of Brd4 or deletion of the core SE region reduces Ahcy expression and exacerbates lipid accumulation in vivo or in vitro. Hnf4a is identified as the transcription factor driving the Ahcy‐SE. In vitro and in vivo experiments demonstrate that IF‐activated Hnf4a directly binds to and activates the Ahcy‐SE. Ahcy knockdown attenuates the lipid‐deposition‐reducing effect of Hnf4a overexpression in mice fed a HFD. In Ahcy^LKO^ mice, the liver SAM/SAH ratio is reduced, and Reduced Representation Bisulfite Sequencing (RRBS) and transcriptome sequencing reveal liver‐specific methylation remodeling. One manifestation of this remodeling is hypermethylation of metabolic gene promoters, including the Acot12 promoter, where aberrant recruitment of Dnmt3b leads to silencing of the gene and impaired lipid hydrolysis. Therefore, these findings define an IF‐Hnf4a‐Ahcy pathway that activates SE‐driven Ahcy to orchestrate protective epigenetic reprogramming in MASLD.

## Graphical abstract

Our study identifies an Hnf4a‐driven super‐enhancer of hepatic Ahcy that mediates the protective effects of intermittent fasting against MASLD. Disruption of the Ahcy super‐enhancer reduces Ahcy expression and exacerbates lipid accumulation. This pathway prevents aberrant promoter hypermethylation by maintaining the SAM/SAH balance, as exemplified by reduced hypermethylation and silencing of Acot12, thereby sustaining lipid hydrolysis and limiting hepatic steatosis.

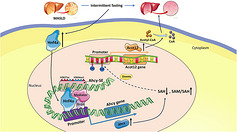

## 1 Introduction

Metabolic dysfunction‐associated steatotic liver disease (MASLD) is the most prevalent chronic liver disease worldwide, and its notably rising incidence in recent years has made it a significant public health challenge [1]. MASLD encompasses a spectrum ranging from simple hepatic steatosis to metabolic dysfunction‐associated steatohepatitis (MASH), and may advance further to liver fibrosis and cirrhosis. MASLD is regarded as the hepatic manifestation of metabolic syndrome, being closely associated with obesity, insulin resistance, and dyslipidemia [2], and frequently co‐occurs with type 2 diabetes and cardiovascular diseases [3]. Despite its substantial health burden, no approved targeted drugs are currently available. Therefore, lifestyle interventions, particularly dietary management, remain the cornerstone of MASLD prevention and treatment [4, 5].

Accumulating evidence underscores the critical role of epigenetic modifications in MASLD pathogenesis. DNA methylation is a key epigenetic mechanism. Global DNA hypomethylation has been observed in MASLD patients and correlates with histological severity [6]. Consistently, aberrant DNA methylation patterns in genes involved in lipid metabolism, inflammation, and fibrosis suggest that epigenetic dysregulation is a fundamental contributor to the disease process [7, 8]. In addition to DNA methylation, histone modifications are also implicated in MASLD. For example, H3K27me3, a repressive histone methylation mark catalyzed by EZH2, is dysregulated in high‑fat diet (HFD)‐induced MASLD liver tissue and has been associated with lipid deposition and fibrosis [9, 10]. The establishment of DNA methylation is centrally governed by the methionine cycle, which regulates cellular methylation potential. This cycle produces the universal methyl donor S‐adenosylmethionine (SAM), which transfers its methyl group to various substrates including DNA, generating S‐adenosylhomocysteine (SAH) [11]. SAH is a potent inhibitor of methyltransferases and is hydrolyzed to homocysteine by S‐adenosylhomocysteine hydrolase (Ahcy) [12, 13]. The SAM:SAH ratio serves as a critical indicator of cellular methylation capacity [12, 13], and perturbations in this balance have been implicated in MASLD‐related metabolic dysregulation. Notably, Ahcy inhibition plays a pivotal role in MASLD pathogenesis. Genetic mutation of Ahcy in zebrafish [14], yeast [15], and Ahcy‐deficient patients [16] promotes hepatic steatosis. In mice, various diets including high‐fat and methyl‐donor‐deficient diets induce MASLD accompanied by reduced hepatic Ahcy expression and elevated SAH levels [17, 18]. In vitro, treatment with the Ahcy inhibitor DZA increases SAH levels and promotes dose‐dependent intracellular triglyceride accumulation and large lipid droplet formation in rat primary hepatocytes [6].

Intermittent fasting (IF), a dietary strategy involving regular cycles of fasting and eating, is recognized for its benefits in weight loss, inflammation reduction, and gut microbiota modulation [19]. Studies in mice demonstrate that IF counteracts HFD‐induced metabolic disturbances, reducing weight gain, improving glucose homeostasis, and alleviating hepatic steatosis and inflammation [20, 21]. Furthermore, IF improves MASH‐related pathological damage and reverses liver fibrosis by increasing hepatic PPARα activity and thereby upregulating PCK1 expression, as reported in a recent study [22]. Although IF represents an effective intervention against MASLD, its specific molecular mechanisms remain incompletely understood. IF induces widespread alterations in epigenetic marks, including DNA methylation, across genes associated with metabolism, inflammation, and cell survival [23, 24]. However, it is still unclear whether IF's therapeutic effects involve modulating the methionine cycle and restoring DNA methylation patterns.

Super‑enhancers (SEs), which are dense clusters of enhancers bound by transcription factors and coactivators such as Brd4 and Mediator, are well known for controlling cell identity genes [25]. Notably, they have also emerged as pivotal regulators of gene expression in metabolic diseases [26]. In HFD models, SE‐associated gene expression positively correlates with hepatic fat accumulation, particularly in lipid metabolism and inflammatory pathways [27]. Specific SEs directly control the expression of metabolic genes such as PCK1 and CYP8B1, influencing hepatic lipid metabolism [28, 29]. The activation of SEs frequently involves recruitment of specific transcription factors that promote expression of metabolism‐related genes, thereby shaping liver pathology [29, 30].

Based on these findings, we hypothesize that IF remodels the SE landscape, thereby driving expression of the methionine cycle‐related enzyme Ahcy and orchestrating protective DNA methylation reprogramming. This study demonstrates that IF ameliorates MASLD by upregulating Ahcy through Hnf4a‐mediated SE activation, leading to restoration of the SAM/SAH ratio and targeted methylation alterations. Our findings define an IF–Hnf4a–Ahcy epigenetic axis that underlies the therapeutic efficacy of this dietary intervention against MASLD.

## 2 Results

### 2.1 SE Remodeling Is an Epigenetic Mechanism Involved in the Amelioration of MASLD by IF

**FIGURE 1:**
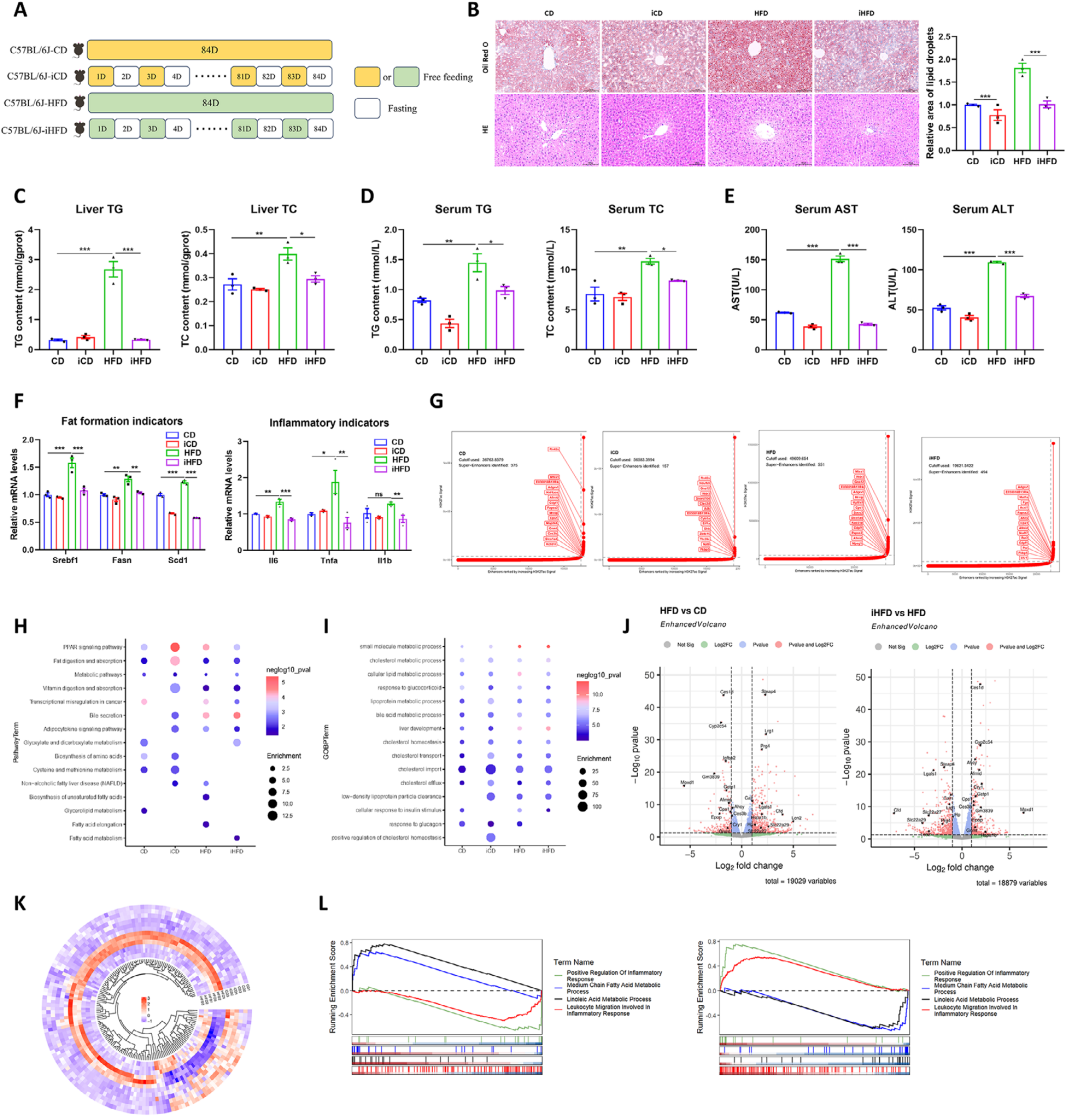
The therapeutic effect of IF on HFD‐induced MASLD is accompanied by SE remodeling. (A) 8‐week‐old male C57BL/6J mice (n = 8) were fed a normal diet or HFD for 12 weeks. CD and HFD groups had ad libitum access to food and water, while iCD and iHFD groups underwent IF protocols. (B) Upper figure: Oil Red O staining of liver tissues and quantitative analysis of lipid droplets (bar graph). Lower figure: HE staining of liver tissues (20×). (C) Liver tissue TG and TC levels in each group. (D) Serum TG and TC levels. (E) Serum ALT and AST levels. (F) The mRNA expression levels of hepatic fat formation indicators Srebf1, Fasn, Scd1, and inflammatory indicators Il6, Tnfa, Il1b. (G) Enhancer hockey stick plots generated using the ROSE algorithm based on H3K27Ac ChIP‐seq signals, with enhancers above the inflection point defined as SEs. (H) Kyoto Encyclopedia of Genes and Genomes (KEGG) analysis results for target genes regulated by SEs. (I) Gene Ontology (GO)‐Biological Process (BP) analysis results for target genes regulated by SEs. (J) Volcano plot of differentially expressed genes (DEGs) between HFD vs CD and iHFD vs HFD groups. (K) Heatmap of DEGs for each sample. (L) Gene set enrichment analysis (GSEA) of inflammation‐related pathways and fatty acid metabolism‐related pathways in the GO‐BP database for HFD vs CD groups and iHFD vs HFD groups. *p < 0.05, **p < 0.01, ***p < 0.001.

Mice fed HFD exhibited hallmark features of MASLD, including increased body and liver weight, elevated fasting blood glucose, insulin resistance (Figure S1A–F), and pronounced hepatic steatosis (Figure 1A,B), with no significant change in liver‐to‐body weight ratio (Figure S1B). This was accompanied by dyslipidemia, elevated liver injury markers (ALT/AST), and upregulation of lipogenic and inflammatory genes (Figure 1C–F). IF under HFD conditions (iHFD) significantly reversed these metabolic perturbations, improving glucose homeostasis and insulin sensitivity (Figure S1A–F), attenuating hepatic steatosis (Figure 1B), and normalizing lipid and inflammatory gene expression profiles (Figure 1F).

To investigate the epigenetic underpinnings of IF's therapeutic effect, we profiled the SE landscape in mouse livers using H3K27ac CUT&RUN. We identified distinct sets of SE‐associated genes across CD, iCD, HFD, and iHFD groups (Figure 1G and Figure S1G). These SEs were significantly enriched for regulators of lipid metabolism, fatty acid synthesis, and inflammatory pathways (Figure 1H), as well as lipid‐related biological processes (Figure 1I) and molecular functions including lipid binding and transport (Figure S1H), positioning SE remodeling as a key mechanism in MASLD progression and IF‐mediated recovery.

### 2.2 Ahcy Is a Key SE‐Driven Effector Essential for the Benefits of IF

To identify the critical SE‐driven genes through which IF ameliorates MASLD, we also characterized the transcriptomic changes. Comparative analysis identified DEGs between the HFD and CD groups, as well as between the iHFD and HFD groups (Figure 1J,K). These DEGs were enriched in lipid metabolism and inflammation‐related pathways (KEGG/GO, Figure S1I,J). GSEA confirmed that IF reversed the HFD‐induced transcriptional profile by promoting fatty acid metabolism and suppressing inflammation (Figure 1L).

**FIGURE 2:**
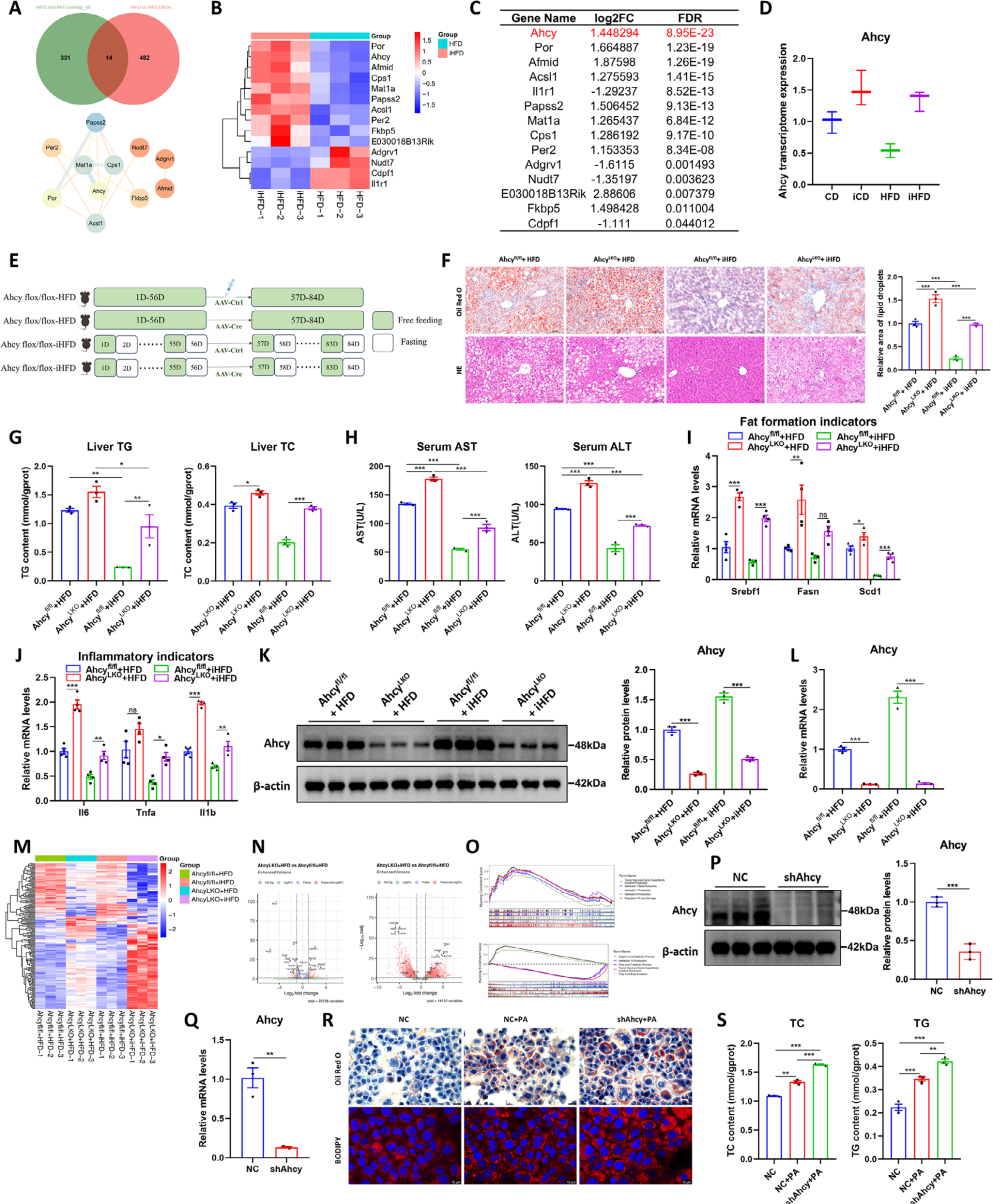
Liver‐specific knockout of Ahcy, a hub SE‐associated gene, attenuates IF‐mediated improvement in MASLD. (A) The intersection of SE target genes overlapping between the iHFD and HFD groups and the DEGs from the iHFD vs HFD group yielded 14 target genes (upper figure), along with their PPI network (lower figure), with discrete points removed. (B) Heatmap of transcriptional expression levels for the 14 target genes. (C) Transcriptome log2 fold change (log2FC) and FDR values for the 14 target genes. (D) Transcriptome expression levels of Ahcy. (E) Mouse Intervention Protocol. 8‐week‐old male Ahcy^fl/fl^ mice (n = 8) were fed a HFD for 12 weeks to induce MASLD. The Ahcy^fl/fl^+HFD and Ahcy^LKO^+HFD groups had ad libitum access to food and water, while the Ahcy^fl/fl^+iHFD and Ahcy^LKO^+iHFD groups underwent IF protocols. After 8 weeks, Ahcy^fl/fl^+HFD and Ahcy^fl/fl^+iHFD groups received tail vein injection of control virus AAV8‐TBG‐Ctrl, while Ahcy^LKO^+HFD and Ahcy^LKO^+iHFD groups received tail vein injection of AAV8‐TBG‐Cre. All groups continued feeding on their respective diets for an additional 4 weeks. (F) Upper figure: Oil Red O staining of liver tissues and quantitative analysis of lipid droplets (bar graph). Lower figure: HE staining of liver tissues (20×). (G) Liver tissue TG and TC levels in each group. (H) Serum ALT and AST levels. (I,J) The mRNA expression levels of hepatic fat formation indicators Srebf1, Fasn, Scd1, and inflammatory indicators Il6, Tnfa, Il1b. (K) The protein expression and quantitative analysis of Ahcy in primary cells of mouse liver. (L) The mRNA expression of Ahcy in primary cells of mouse liver. (M) Heatmap of DEGs for each sample. (N) Volcano plot of DEGs between Ahcy^fl/fl^+HFD vs Ahcy^LKO^+HFD, Ahcy^fl/fl^+iHFD vs Ahcy^LKO^+iHFD groups. (O) GSEA enrichment analysis of inflammation‐related pathways and fatty acid metabolism‐related pathways in the GO‐BP database for Ahcy^LKO^+HFD vs Ahcy^fl/fl^+HFD groups (upper figure) and Ahcy^LKO^+iHFD vs Ahcy^fl/fl^+iHFD groups (lower figure). (P) The protein expression and quantitative analysis of Ahcy in the stable Ahcy‐knockdown AML12 cells. (Q) The mRNA expression of Ahcy in the stable Ahcy‐knockdown AML12 cells. (R) Oil red O staining and BODIPY staining of the stable Ahcy‐knockdown AML12 cells after PA treatment. (S) TC and TG levels in the stable Ahcy‐knockdown AML12 cells after PA treatment. *p < 0.05, **p < 0.01, ***p < 0.001.

To pinpoint key drivers downstream of SE remodeling, we integrated these transcriptomic DEGs (iHFD vs HFD) with SE‐associated genes. This multi‐omics integration yielded 14 high‐confidence candidate genes (Figure 2A,B). Among them, Ahcy showed the most significant upregulation by IF (Figure 2C). Subsequent validation experiments confirmed that a HFD suppressed, while IF restored, hepatic Ahcy expression at both the mRNA and protein levels (Figures 2D and 6A,B).

To establish causality, we generated liver‐specific Ahcy knockout mice (Ahcy^LKO^) (Figure 2K,L) and subjected them to HFD and iHFD interventions (Figure 2E). Strikingly, Ahcy deficiency abolished the protective effects of IF. Compared to the Ahcy^fl/fl^+iHFD group, the Ahcy^LKO^+iHFD group showed no significant differences in liver weight, body weight, liver‐to‐body weight ratio, fasting blood glucose, glucose tolerance, or insulin sensitivity (Figure S2A–F). Instead, they exhibited exacerbated hepatic steatosis (Figure 2F), elevated plasma and liver cholesterol, and increased markers of liver injury (AST, ALT) compared to their IF‐treated controls (Figure 2G,H and Figure S2G). Lipogenic and inflammatory gene expression was also upregulated (Figure 2I,J). Similar exacerbation of MASLD phenotypes was observed in Ahcy^LKO^+HFD mice (Figure 2G–J and Figure S2A–F). Transcriptomic analysis of liver tissues revealed that DEGs from Ahcy^fl/fl^ vs Ahcy^LKO^ comparisons (Figure 2M,N) were enriched in pathways related to steroid and bile acid metabolism, fatty acid metabolism, inflammation, and immune regulation (Figure S2H,I). GSEA further reinforced this finding, revealing that Ahcy loss skewed the transcriptional landscape toward a pro‐inflammatory state and suppressed critical fatty acid metabolism pathways (Figure 2O), underscoring its essential role in mediating IF‐induced metabolic improvement.

To corroborate these in vivo findings and demonstrate a cell‐autonomous role for Ahcy, we turned to an in vitro model. In AML12 cells, Ahcy expression was knocked down using both stable lentiviral transduction and transient siRNA transfection (Figure 2P,Q and Figure S2J,K). Ahcy knockdown exacerbated palmitic acid (PA)‐induced lipid accumulation, with increased intracellular TG and TC levels (Figure 2R,S and Figure S2L,M). Collectively, our in vivo and in vitro loss‐of‐function studies establish Ahcy as a critical mediator of the protective effects of IF against MASLD.

### 2.3 The Ahcy‐SE, Particularly the Enhancer 3 (E3) Component, Drives Its Gene Expression

**FIGURE 3:**
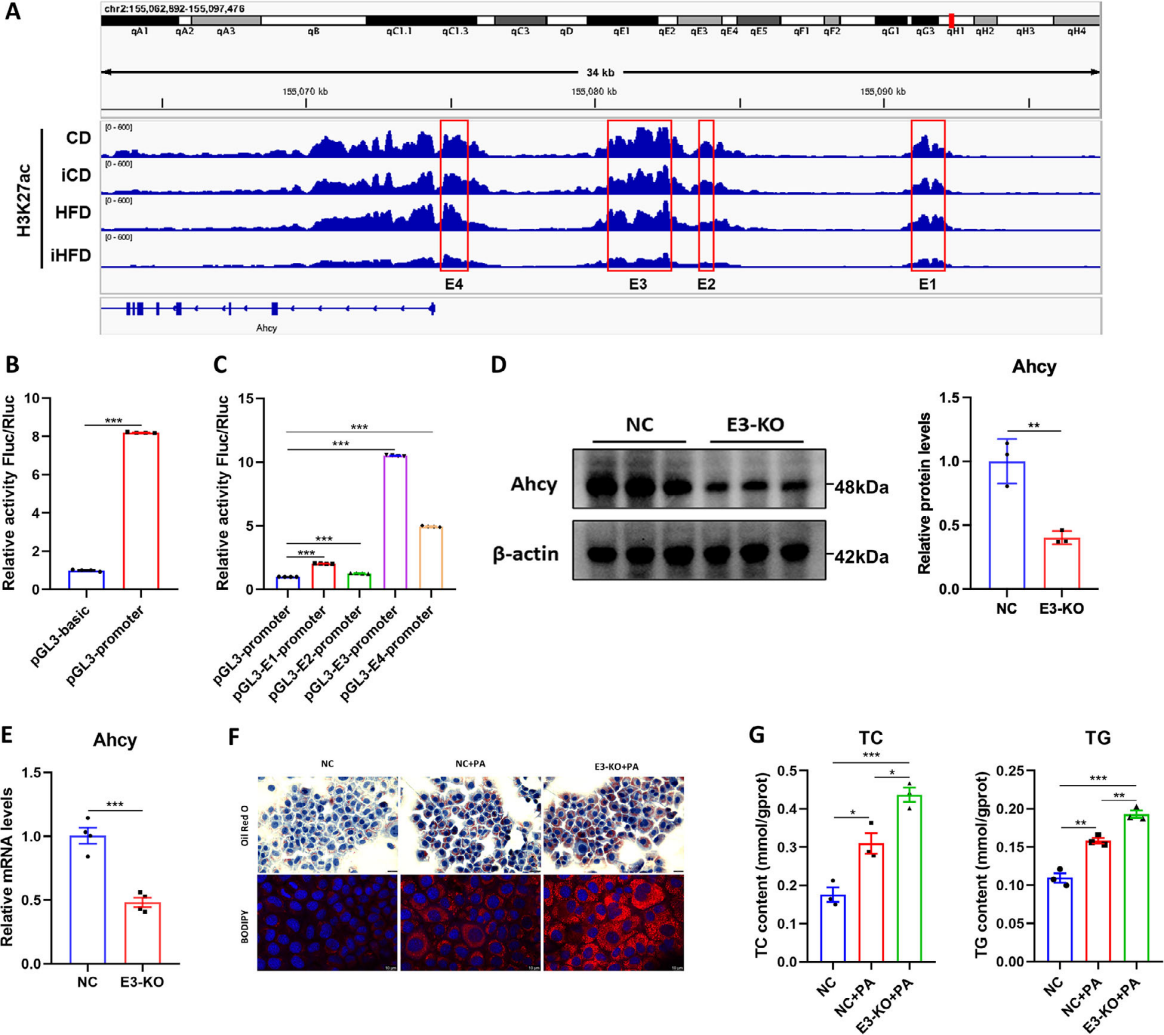
Ahcy is an SE‐driven gene, and the main active component E3 of SE plays an important regulatory role in its transcriptional activity. (A) H3K27ac enrichment and regional segmentation of the Ahcy‐SE. (B) Luciferase reporter gene assays to detect the luciferase activity of the Ahcy promoter. (C) Luciferase reporter gene assays to detect the luciferase activity of the SE active region E1–E4. (D) The protein expression and quantitative analysis of Ahcy after knocking out E3 in AML12 cells. (E) The mRNA expression of Ahcy after knocking out E3 in AML12 cells. (F) Oil Red O and BODIPY staining after PA treatment in AML12 E3‐KO cells. (G) The TC and TG content in AML12 E3‐KO cells after PA treatment. *p < 0.05, **p < 0.01, ***p < 0.001.

We characterized the SE landscape of the Ahcy locus using H3K27ac CUT&RUN profiling in livers from the four experimental groups. A ∼21 kb SE region was identified 15.4 kb upstream of the Ahcy gene (Figure 3A), consistent with public H3K27ac ChIP‐seq data from CD and HFD mouse livers (Figure S3A). This SE was subdivided into four components (E1–E4) based on H3K27ac enrichment peaks (Figure 3A and Figure S3A). Hi‐C analysis from mouse liver in public databases further revealed a spatial interaction between the SE and the Ahcy promoter region (Figure S3B), suggesting potential chromatin looping.

To functionally dissect the Ahcy‐SE, we performed luciferase reporter gene assays using constructs containing the Ahcy promoter alone or in combination with individual SE components (E1–E4). While the promoter alone showed activity above the empty vector (Figure 3B), all SE components significantly enhanced promoter activity (Figure 3C). Among these, E3 exhibited the strongest effect, indicating it as the primary active sub‐region of the SE (Figure 3C).

We further evaluated the necessity of E3 for Ahcy expression by deleting this region using CRISPR‐Cas9 in AML12 cells (Figure S3C,D). E3 deletion led to a marked reduction in both Ahcy mRNA and protein levels (Figure 3D,E). Moreover, upon palmitic acid (PA) challenge, E3‐knockout cells displayed exacerbated lipid accumulation, as evidenced by Oil Red O and BODIPY staining, with increased intracellular TC and TG content (Figure 3F,G). These results establish the Ahcy‐SE, and specifically the E3 component, as a critical regulator of Ahcy transcription and hepatic lipid homeostasis.

### 2.4 Brd4 Inhibition Blunts both Ahcy SE Activity and the Efficacy of IF

**FIGURE 4:**
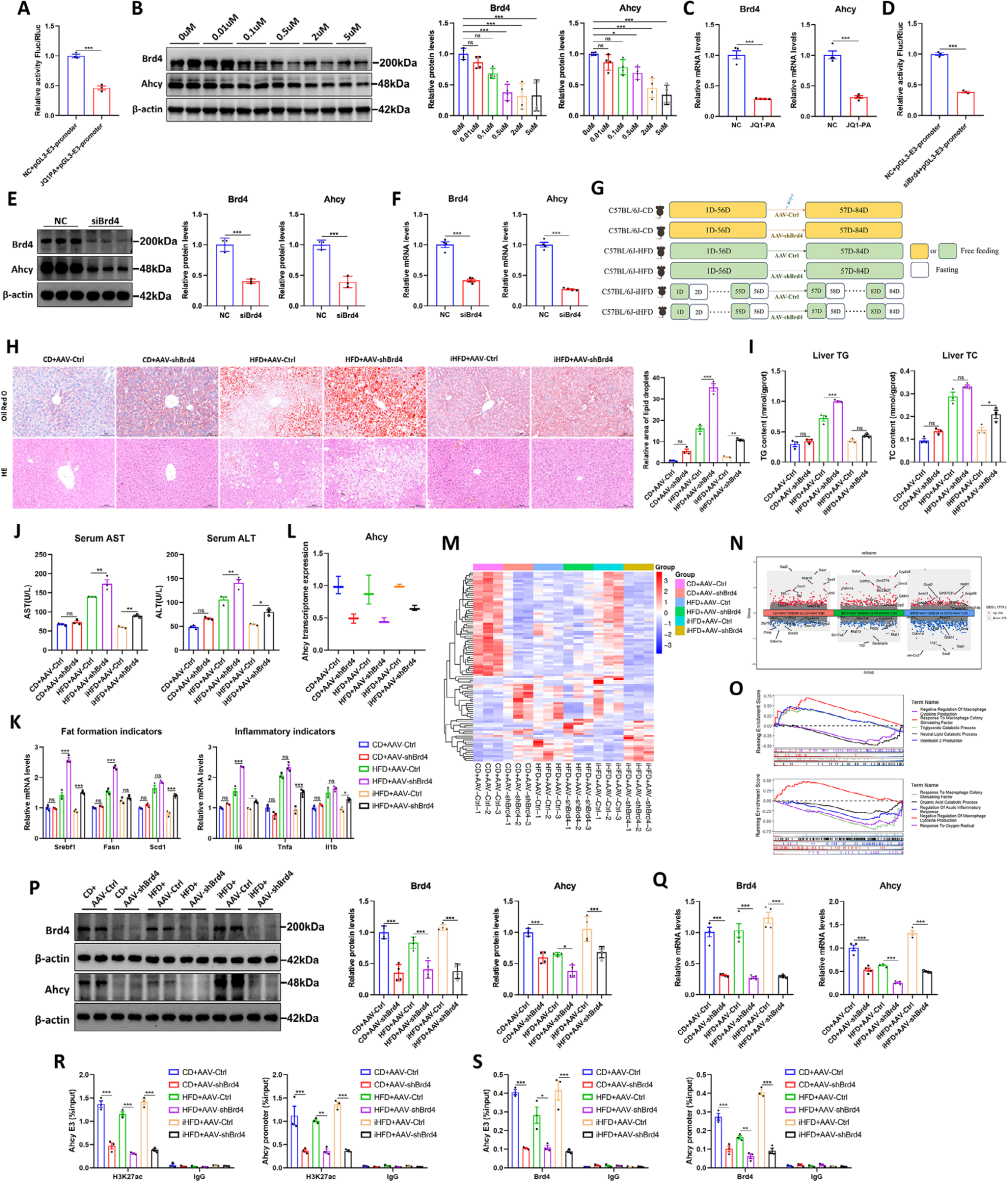
Brd4 depletion inhibits the expression of Ahcy, aggravates the progression of MASLD, and attenuates the therapeutic effects of IF. (A) Luciferase reporter gene assays to detect the luciferase activity of E3 after JQ1‐PA (5 µM) treatment. (B) The protein expression and quantitative analysis of Brd4 and Ahcy after 48 h of JQ1‐PA treatment. (C) The mRNA expression of Brd4 and Ahcy after 48 h of JQ1‐PA (5uM) treatment. (D) Luciferase reporter gene assays to detect the luciferase activity of E3 after transfection with siBrd4. (E) The protein expression and quantitative analysis of Brd4 and Ahcy after 72 h of siBrd4 (50 nM) treatment. (F) The mRNA expression of Brd4 and Ahcy after 72 h of siBrd4 (50 nM) treatment. (G) Mouse Intervention Protocol. 8‐week‐old male C57BL/6J mice (n = 10) were fed either a normal diet or an HFD for 12 weeks. The CD+AAV‐shBrd4 group, CD+AAV‐Ctrl group, HFD+AAV‐shBrd4 group, and HFD+AAV‐Ctrl group had ad libitum access to food and water. The iHFD+AAV‐shBrd4 and iHFD+AAV‐Ctrl groups underwent IF protocols. After 8 weeks, the CD+AAV‐shBrd4, HFD+AAV‐shBrd4, and iHFD+AAV‐shBrd4 groups received tail vein injections of AAV8‐shBrd4, while the CD+AAV‐Ctrl, HFD+AAV‐Ctrl, and iHFD+AAV‐Ctrl groups received tail vein injections of AAV8‐Ctrl. All groups continued their respective diets for an additional 4 weeks. (H) Upper figure: Oil Red O staining of liver tissues and quantitative analysis of lipid droplets (bar graph). Lower figure: HE staining of liver tissues (20×). (I) Liver tissue TG and TC levels in each group. (J) Serum ALT and AST levels. (K) The mRNA expression levels of hepatic fat formation indicators Srebf1, Fasn, Scd1, and inflammatory indicators Il6, Tnfa, Il1b. (L) Transcriptome expression of Ahcy. (M) Heatmap of DEGs for each sample. (N) Volcano plot of DEGs between CD+AAV‐shBrd4 vs CD+AAV‐Ctrl, HFD+AAV‐shBrd4 vs HFD+AAV‐Ctrl, and iHFD+AAV‐shBrd4 vs iHFD+AAV‐Ctrl groups. (O) GSEA enrichment analysis of inflammation‐related pathways and fatty acid metabolism‐related pathways in the GO‐BP database for HFD+AAV‐Ctrl vs iHFD+AAV‐Ctrl groups (left figure) and iHFD+AAV‐shBrd4 vs iHFD+AAV‐Ctrl groups (right figure). (P) The protein expression and quantitative analysis of Brd4 and Ahcy in primary cells of mouse liver. (Q) The mRNA expression of Brd4 and Ahcy in primary cells of mouse liver. (R) ChIP‐qPCR to detect the enrichment of H3K27ac on the Ahcy‐E3 and promoter in mouse liver. (S) ChIP‐qPCR to detect the enrichment of Brd4 on Ahcy‐E3 and the promoter in mouse liver. *p<0.05, **p<0.01, ***p<0.001.

Having established the presence of a critical SE at the Ahcy locus, we next investigated whether this SE was functionally dependent on Brd4, a core architectural component and indispensable transcriptional engine for SE‐driven genes [31, 32]. To address this, we inhibited Brd4 using JQ1‐PA. In the luciferase assay, 5 µM JQ1‐PA significantly reduced the activity of the core enhancer component E3 (Figure 4A). Consistently, JQ1‐PA treatment led to dose‐dependent reductions in Ahcy mRNA and protein levels (Figure 4B,C and Figure S4A,B), with the strongest suppression observed at 5 µM. The essential role of Brd4 was further supported by siRNA‐mediated knockdown, which suppressed both E3 luciferase activity (Figure 4D) and Ahcy expression in AML12 cells (Figure 4E,F and Figure S4C,D).

To evaluate the functional relevance of Brd4 in MASLD and IF, we knocked down Brd4 in mouse liver via AAV‐shBrd4 delivery (Figure 4G). Compared to iHFD+AAV‐Ctrl mice, iHFD+AAV‐shBrd4 mice showed increased liver weight but unchanged liver‐to‐body weight ratio, elevated fasting blood glucose, impaired glucose tolerance, and worsened insulin resistance (Figure S4E,J). Hepatic lipid deposition was aggravated (Figure 4H), accompanied by increased plasma and hepatic TG and TC levels, elevated AST and ALT (Figure 4I,J and Figure S4K), and upregulation of lipogenic and inflammatory genes (Figure 4K). Similar exacerbation of MASLD phenotypes was observed in HFD+AAV‐shBrd4 mice compared to HFD+AAV‐Ctrl mice (Figure 4H–K and Figure S4E–K).

We performed transcriptomic analysis on liver tissues from six mouse groups and visualized DEGs identified from the following comparisons: CD+AAV‐shBrd4 vs CD+AAV‐Ctrl, HFD+AAV‐shBrd4 vs HFD+AAV‐Ctrl, and iHFD+AAV‐shBrd4 vs iHFD+AAV‐Ctrl (Figure 4M,N). Expression of the Ahcy gene in RNA‐seq data across groups was shown in Figure 4L. Ahcy expression was downregulated in Brd4‐knockdown groups compared with their respective controls (Figure 4L). KEGG and GO enrichment analyses of the DEGs revealed significant involvement in steroid hormone biosynthesis, inflammatory response, fatty acid synthesis and degradation, and amino acid metabolism (KEGG, Figure S4L), as well as lipid and fatty acid metabolism, glucose metabolism, and glutathione transferase activity (GO, Figure S4M). GSEA further demonstrated that, compared to the iHFD+AAV‐Ctrl group, the iHFD+AAV‐shBrd4 group exhibited upregulation of inflammation‐related pathways and downregulation of those involved in fatty acid metabolism, effectively counteracting the regulatory benefits conferred by IF (Figure 4O). These results indicate that Brd4 knockdown not only accelerates MASLD progression but also attenuates the therapeutic effects of IF on the disease.

Primary hepatocytes were isolated from mouse livers for protein and RNA extraction. Brd4 knockdown downregulated both Ahcy mRNA and protein expression compared with controls (Figure 4P,Q). Consistently, Brd4 knockdown also led to significantly reduced enrichment of H3K27ac and Brd4 at the Ahcy‐E3 and promoter regions, as shown by ChIP‐qPCR (Figure 4R,S). Collectively, these results demonstrate that SE‐driven expression of Ahcy is regulated by Brd4. Furthermore, inhibition of Brd4 exacerbates MASLD progression and diminishes the therapeutic benefits of IF.

### 2.5 Hnf4a Directly Targets the Ahcy‐SE and Promoter Regions to Drive Its Expression and Alleviate MASLD

**FIGURE 5:**
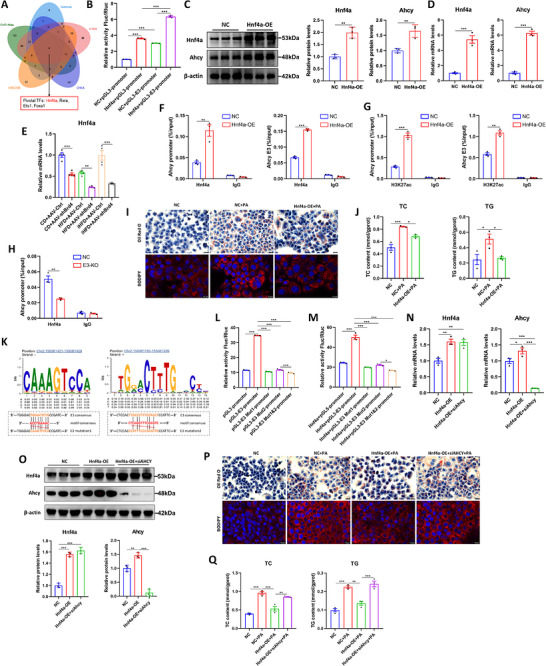
Hnf4a activates Ahcy transcription by binding to the Ahcy‐SE and promoter regions. (A) Intersection of transcription factors predicted to bind the Ahcy gene across five databases: Cistrome, ChIP‐Atlas, GTRD, ENCODE, and CHEA. (B) Luciferase reporter gene assays detected the luciferase activity of the Ahcy‐E3 and promoter after overexpression of Hnf4a. (C) The protein expression and quantitative analysis of Hnf4a and Ahcy in the stable Hnf4a‐overexpressing AML12 cells. (D) The mRNA expression of Hnf4a and Ahcy in the stable Hnf4a‐overexpressing AML12 cells. (E) The mRNA expression of Hnf4a after knockdown of Brd4 in the liver of each group of mice. (F) In the stable Hnf4a‐overexpressing AML12 cells, ChIP‐qPCR was used to detect the enrichment of Hnf4a at the Ahcy‐E3 and promoter. (G) In the stable Hnf4a‐overexpressing AML12 cells, ChIP‐qPCR was used to detect the enrichment of H3K27ac at the Ahcy‐E3 and promoter. (H) In AML12 E3‐KO cells, ChIP‐qPCR was used to detect the enrichment of Hnf4a at the Ahcy promoter. (I) Oil Red O and BODIPY staining of the stable Hnf4a‐overexpressing AML12 cells after PA treatment. (J) TC and TG content in the stable Hnf4a‐overexpressing AML12 cells after PA treatment. (K) Predicted Hnf4a binding sites 1 and 2 within Ahcy‐E3 and their corresponding mutated sequences 1 and 2. L. Luciferase reporter gene assays to detect the baseline luciferase activity of Ahcy‐E3 after mutation of the Hnf4a binding sites. (M) After mutation of the Hnf4a binding site, luciferase reporter gene assays were used to detect luciferase activity of Ahcy‐E3 after overexpression of Hnf4a. (N) The mRNA expression of Hnf4a and Ahcy after knocking down Ahcy in the stable Hnf4a‐overexpressing AML12 cells. (O) The protein expression and quantitative analysis of Hnf4a and Ahcy after knocking down Ahcy in the stable Hnf4a‐overexpressing AML12 cells. (P) After knockdown of Ahcy, Oil Red O and BODIPY staining of the stable Hnf4a‐overexpressing AML12 cells treated with PA. (Q) After knocking down Ahcy in the stable Hnf4a‐overexpressing AML12 cells, the contents of TC and TG in cells. *p<0.05, **p<0.01, ***p<0.001.

**FIGURE 6:**
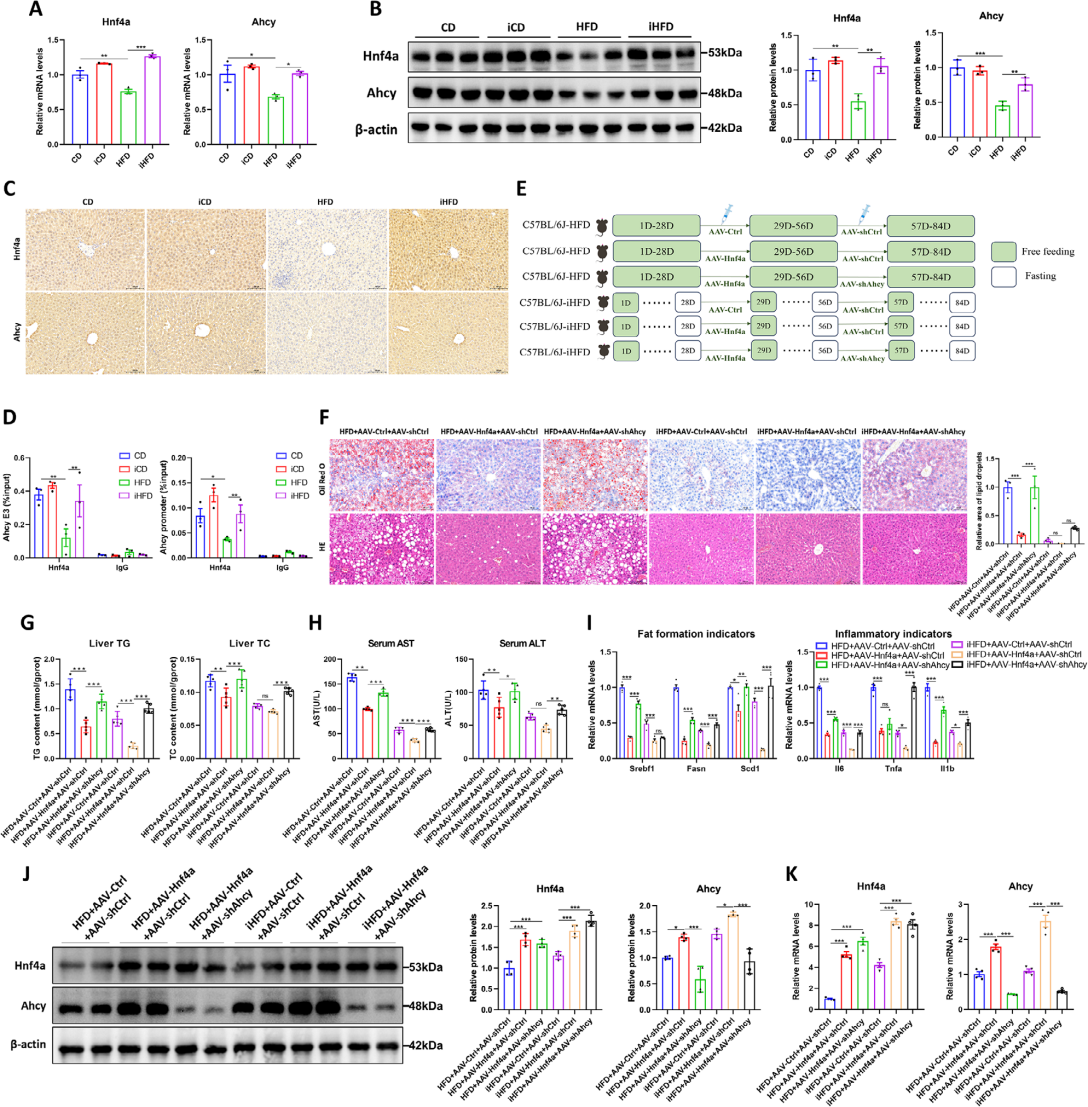
Hnf4a alleviates hepatic lesions in MASLD mice by promoting the expression of Ahcy in vivo. (A) The mRNA expression of Hnf4a and Ahcy in primary liver cells of mice in the CD, iCD, HFD, and iHFD groups. (B) The protein expression and quantitative analysis of Hnf4a and Ahcy in primary liver cells of mice in the CD, iCD, HFD, and iHFD groups. (C) Immunohistochemical results of Hnf4a and Ahcy in the liver of mice in the CD, iCD, HFD, and iHFD groups. D. ChIP‐qPCR to detect the enrichment of Hnf4a on Ahcy‐E3 and the promoter in the liver of mice in the CD, iCD, HFD, and iHFD groups. (E) Mouse Intervention Protocol. 8‐week‐old male C57BL/6J mice (n = 8) were fed an HFD for 12 weeks to induce NAFLD. The HFD+AAV‐Ctrl+AAV‐shCtrl group, HFD+AAV‐Hnf4a+AAV‐shCtrl group, and HFD+AAV‐Hnf4a+AAV‐shAhcy group had ad libitum access to food and water. The iHFD+AAV‐Ctrl+AAV‐shCtrl group, iHFD+AAV‐Hnf4a+AAV‐shCtrl group, and iHFD+AAV‐Hnf4a+AAV‐shAhcy group underwent IF protocols. After 4 weeks, the HFD+AAV‐Ctrl+AAV‐shCtrl group and iHFD+AAV‐Ctrl+AAV‐shCtrl group received tail vein injections of control virus AAV8‐Ctrl. The HFD+AAV‐Hnf4a+AAV‐shCtrl group, HFD+AAV‐Hnf4a+AAV‐shAhcy group, iHFD+AAV‐Hnf4a+AAV‐shCtrl group, and iHFD+AAV‐Hnf4a+AAV‐shAhcy group received tail vein injections of AAV8‐Hnf4a. After 4 weeks, the HFD+AAV‐Ctrl+AAV‐shCtrl group, HFD+AAV‐Hnf4a+AAV‐shCtrl group, iHFD+AAV‐Ctrl+AAV‐shCtrl group, and iHFD+AAV‐Hnf4a+AAV‐shCtrl group received tail vein injections of the control virus AAV8‐Ctrl. The HFD+AAV‐Hnf4a+AAV‐shAhcy group and iHFD+AAV‐Hnf4a+AAV‐shAhcy group received tail vein injections of AAV8‐shAhcy. All groups continued their respective diets for an additional 4 weeks. (F) Upper figure: Oil Red O staining of liver tissues and quantitative analysis of lipid droplets (bar graph). Lower figure: HE staining of liver tissues (20×). (G) Liver tissue TG and TC levels in each group. (H) Serum ALT and AST levels. (I) The mRNA expression levels of hepatic fat formation indicators Srebf1, Fasn, Scd1, and inflammatory indicators Il6, Tnfa, Il1b. (J) The protein expression and quantitative analysis of Hnf4a and Ahcy in primary cells of mouse liver. (K) The mRNA expression of Hnf4a and Ahcy in primary liver cells of mice. *p<0.05, **p<0.01, ***p<0.001.

To identify the transcription factor initiating the Ahcy‐SE, we first integrated predictions from five databases, which yielded four candidate TFs (Figure 5A). JASPAR analysis nominated Hnf4a as the top candidate, based on its highest predicted binding affinity for both the Ahcy SE and promoter (Tables S5 and S6). Supporting this prediction, IF elevated Hnf4a mRNA and protein levels in mouse livers, a trend that paralleled the induction of Ahcy (Figure 6A–C). We then gathered multiple lines of evidence to confirm Hnf4a's direct role. First, interrogation of public ChIP‐seq data (GSE118007) revealed a strong binding peak of Hnf4a within the key SE component E3 (Figure S5A). Protein‐DNA docking simulations further supported the spatial binding capability of the Hnf4a protein to the Ahcy promoter and E3 region (Figure S5B). Ultimately, ChIP‐qPCR in vivo confirmed that Hnf4a enrichment at the Ahcy promoter and E3, which was diminished by HFD, could be effectively restored by IF (Figure 6D).

Functionally, Hnf4a overexpression potently activated the Ahcy promoter and E3 segment in luciferase assays (Figure 5B) and upregulated endogenous Ahcy expression in AML12 cells (Figure 5C,D). Corroborating these findings, ChIP‐qPCR in stable Hnf4a‐overexpressing cells showed enhanced enrichment of Hnf4a and the active mark H3K27ac at the Ahcy promoter and E3 (Figure 5F,G). Conversely, CRISPR–Cas9‐mediated E3 deletion diminished both Ahcy expression and Hnf4a binding at the promoter (Figure 3D,E and Figure 5H). Furthermore, Brd4 knockdown reduced hepatic Hnf4a expression (Figure 5E), positioning Hnf4a downstream of Brd4 in the regulatory pathway controlling Ahcy.

The functional necessity of Hnf4a binding was confirmed using luciferase reporters containing mutations in the predicted Hnf4a motifs within E3, which significantly reduced both basal and Hnf4a‐induced luciferase activity (Figure 5K,M). Functionally, Hnf4a overexpression attenuated PA‐induced lipid accumulation and reduced intracellular TG and TC levels in AML12 cells (Figure 5I,J). Crucially, knockdown of Ahcy in Hnf4a‐overexpressing cells attenuated this lipid‐lowering effect, increasing intracellular lipid deposition and TG/TC levels (Figure 5N–Q). Collectively, these results demonstrate that Hnf4a directly binds to the Ahcy‐E3 and promoter to activate its transcription, thereby ameliorating hepatic steatosis.

To determine whether Hnf4a ameliorates MASLD through Ahcy in vivo, we co‐administered AAV‐Hnf4a and AAV‐shAhcy to mice (Figure 6E). Compared with HFD+AAV‐Ctrl+AAV‐shCtrl controls, Hnf4a‐overexpressing mice exhibited reduced fasting blood glucose, improved glucose tolerance, alleviated insulin resistance, and attenuated hepatic steatosis (Figure S5G,H and Figure 6F). These improvements were accompanied by lower liver weight and liver‐to‐body weight ratio, decreased TG/TC levels, lower AST/ALT, and downregulated lipogenic and inflammatory gene expression, despite no significant changes in body weight or cumulative food intake (Figure 6G–I and Figure S5C–F, I). Notably, concurrent Ahcy knockdown reversed these protective effects, leading to increased liver weight, elevated fasting glucose, exacerbated insulin resistance, enhanced hepatic lipid deposition, and upregulation of injury markers and lipogenic/inflammatory genes, with no significant change in body weight (Figure 6F–I and Figure S5C–I). Similar antagonistic effects were observed in iHFD‐fed mice (Figure 6F–I and Figure S5C–I). Consistent with the in vivo findings, Ahcy knockdown abolished the lipid‐lowering effect of Hnf4a overexpression in AML12 hepatocytes (Figure 5N–Q). In primary hepatocytes, the expression levels of Hnf4a and Ahcy confirmed the efficiency of viral infection (Figure 6J–K). Hnf4a overexpression enhanced Ahcy expression at both mRNA and protein levels (Figure 6J–K). These results establish Ahcy as an essential downstream effector through which Hnf4a ameliorates MASLD.

### 2.6 Ahcy Governs DNA Methylation Homeostasis for Lipid Metabolic Gene Expression

**FIGURE 7:**
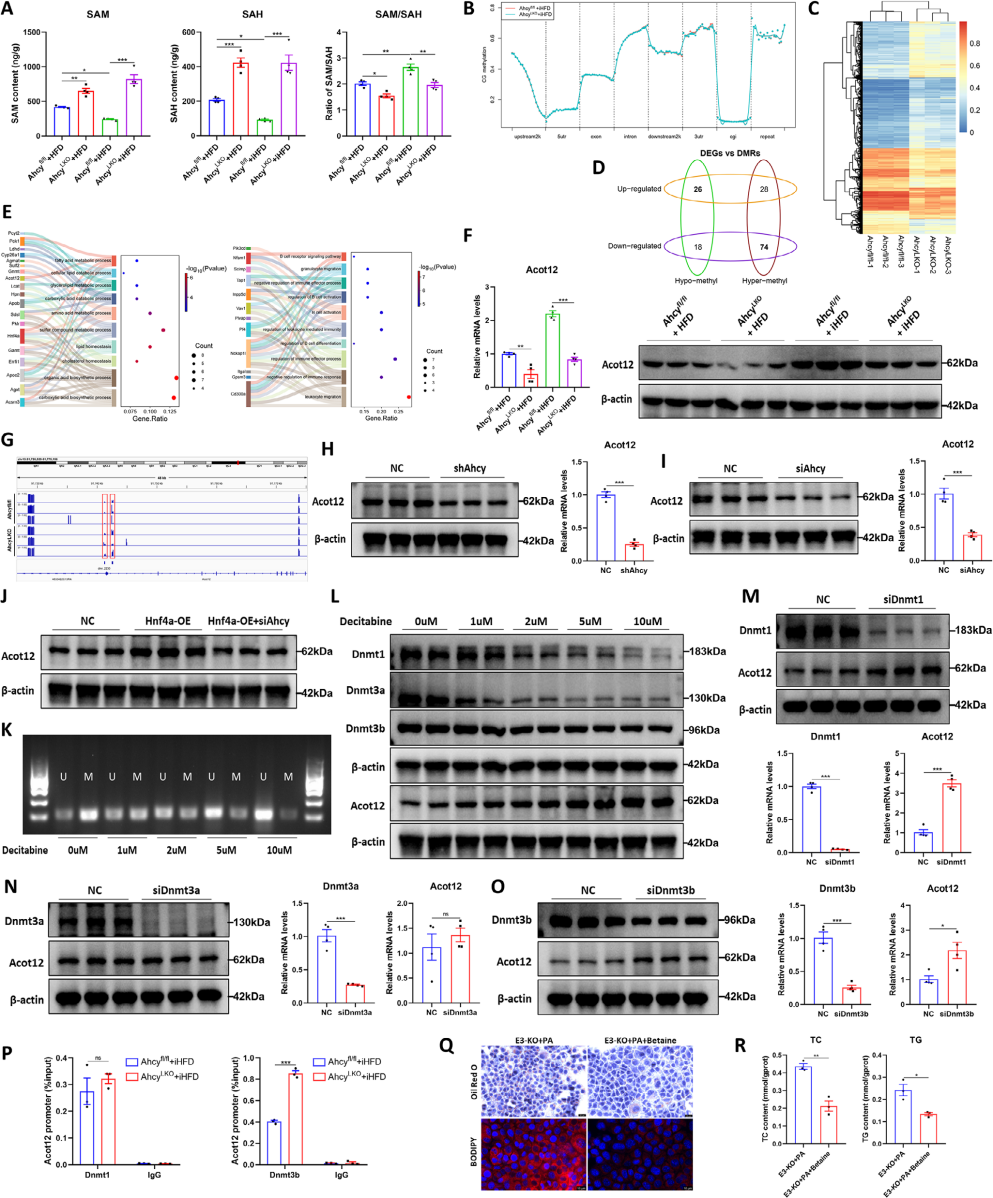
Knockout of Ahcy increases the methylation level of the Acot12 promoter, thus reducing its expression. (A) The values of SAM, SAH, and SAM/SAH in the liver of mice in each group after Ahcy knockout. (B) Methylation level maps of genomic functional elements across groups. (C) Heatmap of DMR methylation level clustering. (D) Association analysis between genes with DMR‐anchored promoter‐associated genes and DEGs in the transcriptome. (E) GO‐BP enrichment analysis results for genes with decreased expression and increased promoter methylation (left figure), and genes with increased expression and decreased promoter methylation (right figure). (F) The mRNA and protein expression of Acot12 in each group of mice after liver‐specific Ahcy knockout. (G) IGV visualization of the DMR region of the Acot12 gene promoter in two groups. (H) The mRNA and protein expression of Acot12 in the stable Ahcy‐knockdown AML12 cells. (I) The mRNA and protein expression of Acot12 after siAhcy treatment in AML12 cells. (J) The protein expression of Acot12 after knocking down Ahcy in the stable Hnf4a‐overexpressing AML12 cells. (K) Effects of different concentrations of decitabine on DMR methylation level of Acot12 in the stable Ahcy‐knockdown AML12 cells. (L) Effects of different concentrations of decitabine on the expression of DNA methyltransferase and Acot12 protein in the stable Ahcy‐knockdown AML12 cells. (M) The mRNA and protein expression of Dnmt1 and Acot12 after intervention with siDnmt1 in AML12 cells. (N) The mRNA and protein expression of Dnmt3a and Acot12 after intervention with siDnmt3a in AML12 cells. (O) The mRNA and protein expression of Dnmt3b and Acot12 after intervention with siDnmt3b in AML12 cells. (P) ChIP‐qPCR to detect the enrichment of Dnmt1 and Dnmt3b on the Acot12 promoter in the liver of mice in Ahcy^fl/fl^+iHFD and Ahcy^LKO^+iHFD groups. (Q) Oil Red O and BODIPY staining of the E3‐KO AML12 cells after betaine treatment. (R) TC and TG content in the E3‐KO AML12 cells after betaine treatment. *p < 0.05, **p < 0.01, ***p < 0.001.

In mammals, Ahcy serves as the sole enzyme responsible for hydrolyzing S‐adenosylhomocysteine (SAH), a potent inhibitor of methyltransferase activity, making the SAM/SAH ratio a critical determinant of cellular methylation potential [11, 12, 33]. To define Ahcy's role in hepatic methylation, we first measured SAM and SAH levels in liver‐specific Ahcy knockout mice under HFD and IF regimens. IF treatment reduced both SAM and SAH levels while increasing the SAM/SAH ratio. Notably, Ahcy knockout significantly elevated hepatic concentrations of both metabolites under both dietary conditions compared to Ahcy^fl/fl^ controls, resulting in a decrease in the SAM/SAH ratio (Figure 7A).

We next performed reduced representation bisulfite sequencing (RRBS) on liver tissues from Ahcy^fl/fl^ and Ahcy^LKO^ mice under IF conditions to assess the impact on DNA methylation. Global methylation analysis revealed minimal changes following Ahcy knockout (Figure 7B), though differential methylation region (DMR) analysis identified 3067 hypermethylated and 2130 hypomethylated regions (Figure S6B). Approximately 10% of these DMRs localized to promoter regions within 2 kb upstream of transcription start sites (Figure S6A). The genomic distribution of DMRs varied across chromosomes (Figure S6C). Cluster analysis of intergroup methylation patterns demonstrated clear separation between genotypes (Figure 7C). KEGG/GO enrichment analyses indicated that DMR‐associated genes were primarily involved in fatty acid metabolism and transcriptional regulation pathways (Figure S6D–E). Integration of methylation and transcriptomic data identified 146 promoter‐associated DMRs with correlated expression changes. Among these, 26 hypomethylated DMRs showed increased transcriptional activity and were enriched for immune process regulation, immune cell differentiation, and activation. Conversely, 74 hypermethylated DMRs exhibited reduced expression and were enriched in lipid and amino acid metabolism pathways, along with organic acid biosynthesis (Figure 7D–E).

Hypomethylated DMRs with increased transcription were enriched for immune processes, whereas hypermethylated DMRs with suppressed expression were linked to lipid and amino acid metabolism (Figure 7E). From the latter category, we focused on Acot12, a known regulator of lipogenesis and cholesterol deposition. Acot12 hydrolyzes long‐chain acyl‐CoA to produce free fatty acids, directly regulating the intracellular acyl‐CoA pool, and represents a key rate‐limiting step in lipid synthesis and lipid droplet accumulation [34, 35]. Liver‐specific Ahcy knockout significantly reduced Acot12 mRNA and protein levels (Figure 7F and Figure S7A). By analyzing the DMRs in the Acot12 promoter region and its surrounding CpG islands (Figure 7G and Figure S6F), we examined the methylation status using methylation‐specific PCR (MSP) experiments. The result confirmed Acot12 promoter hypermethylation (Figure S6G) concomitant with suppressed Acot12 expression (Figure 7H and Figure S7B) in the stable Ahcy‐knockdown AML12 cells. This finding was further replicated with siRNA‐mediated knockdown (Figure 7I and Figures S7C, S6H). We next positioned Ahcy within the regulatory hierarchy. In mice and AML12 cells, overexpression of Hnf4a led to increased Acot12 mRNA and protein expression, but this induction was eliminated by simultaneous knockdown of Ahcy (Figure 7J and Figures S7D, S6I,J), establishing Ahcy as a downstream effector of Hnf4a.

To directly establish the causal role of DNA methylation in Acot12 suppression, we treated the stable Ahcy‐knockdown AML12 cells with the Dnmt inhibitor decitabine, which effectively prevented Acot12 promoter hypermethylation and restored its expression in a dose‐dependent manner (Figure 7K,L and Figure S7E). Correspondingly, the protein levels of methyltransferases including Dnmt1, Dnmt3a, and Dnmt3b showed parallel dose‐dependent reductions (Figure 7K,L and Figure S7E). To identify the specific Dnmt isoform mediating this effect, we performed individual knockdown of each Dnmt. Silencing of either Dnmt1 or Dnmt3b, but not Dnmt3a, significantly increased Acot12 expression (Figure 7M–O and Figure S7F–H). Most conclusively, ChIP‐qPCR analysis demonstrated that liver‐specific Ahcy knockout specifically enhanced Dnmt3b enrichment at the Acot12 promoter, while Dnmt1 binding remained unchanged (Figure 7P), thus identifying Dnmt3b as the primary mediator of Ahcy‐mediated site‐specific hypermethylation at this metabolic gene.

Significantly, supplementing the methyl pool with betaine attenuated palmitate‐induced lipid accumulation and reduced TC and TG levels in Ahcy‐E3‐KO AML12 cells (Figure 7Q,R). To clarify the effect of betaine supplementation on Ahcy deficiency in vivo, we knocked down hepatic Ahcy expression and administered betaine intervention in mice (Figure S8A). Compared to the HFD+AAV‐shAhcy+NC group, the HFD+AAV‐shAhcy+Betaine group showed reduced liver weight and body weight, with a concomitant decrease in liver‐to‐body weight ratio (Figure S8B–D), decreased fasting blood glucose (Figure S8E), and improved glucose tolerance and insulin resistance (Figure S8F). Hepatic lipid deposition was alleviated (Figure S8K), accompanied by decreased levels of plasma and liver TG and TC (Figure S8G,H), reductions in AST and ALT (Figure S8I), and downregulation of adipogenesis and inflammatory indicators (Figure S8J). Similar improvements were observed in the iHFD+AAV‐shAhcy+Betaine group compared to the iHFD+AAV‐shAhcy+NC group (Figure S8). These results demonstrate that restoring the availability of methyl donors functionally ameliorates metabolic defects caused by Ahcy deficiency.

### 2.7 Ahcy Is Required for the Protective Effects of IF Against MASH

**FIGURE 8:**
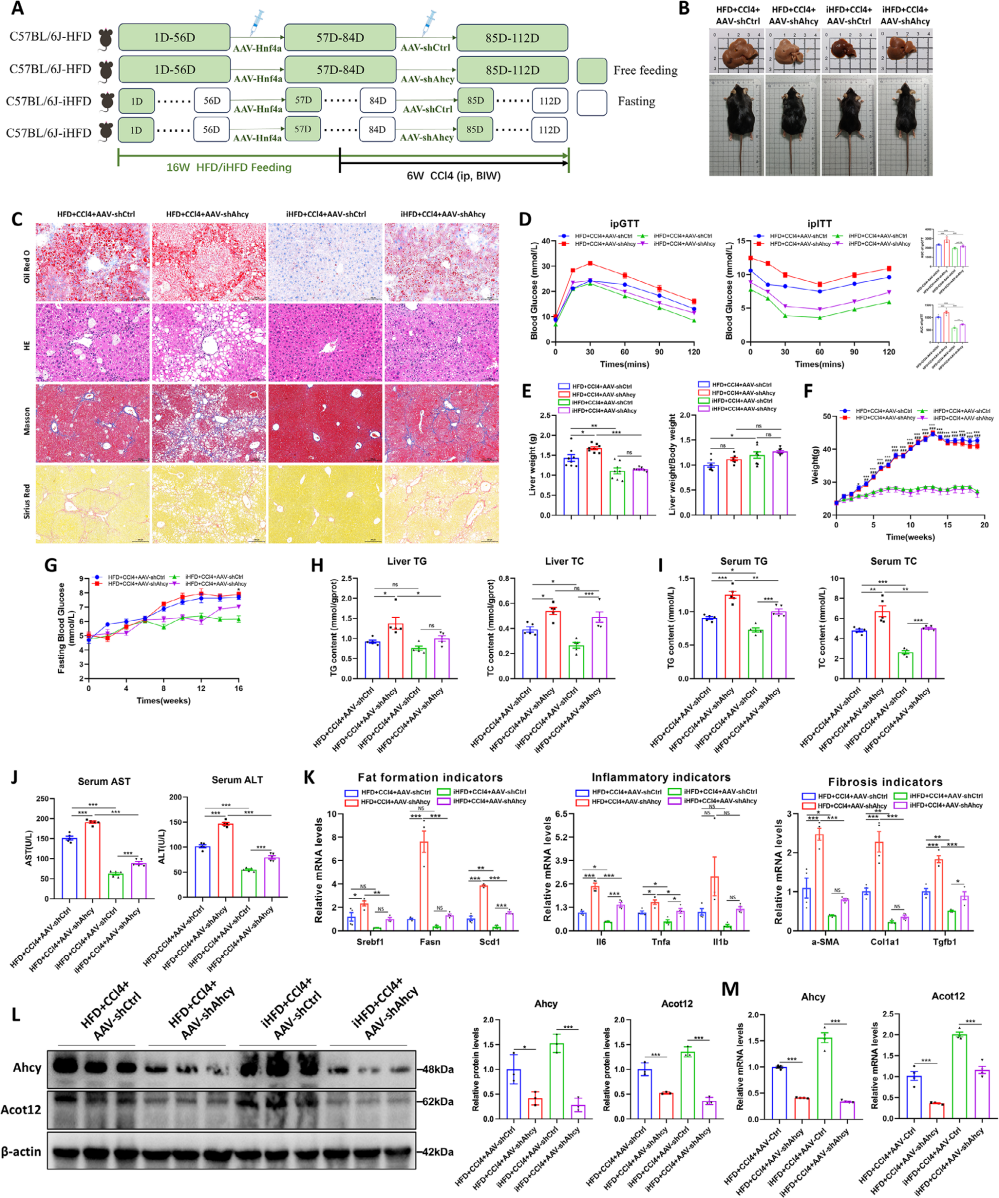
IF alleviates the progression of MASH, and Ahcy knockdown exacerbates hepatic lesions in MASH mice and diminishes the effects of IF. (A) Mouse Intervention Protocol. 8‐week‐old male C57BL/6J mice (n = 8) were fed with HFD and injected with CCl4 intraperitoneally to induce MASH. The HFD+AAV‐Hnf4a+AAV‐shCtrl and HFD+AAV‐Hnf4a+AAV‐shAhcy groups had ad libitum access to food and water, while the iHFD+AAV‐Hnf4a+AAV‐shCtrl and iHFD+AAV‐Hnf4a+AAV‐shAhcy groups underwent IF protocols. After 8 weeks, all groups received a tail vein injection of AAV8‐Hnf4a. Following 2 weeks of continued feeding on the respective dietary models, CCl4 was administered via intraperitoneal injection twice weekly until the end of the experiment. After an additional 2 weeks on the respective diets, the HFD+AAV‐Hnf4a+AAV‐shCtrl and iHFD+AAV‐Hnf4a+AAV‐shCtrl groups received tail vein injections of control virus AAV8‐shCtrl. The HFD+AAV‐Hnf4a+AAV‐shAhcy group and the iHFD+AAV‐Hnf4a+AAV‐shAhcy group received tail vein injections of AAV8‐shAhcy. All groups continued feeding on their respective diets for an additional 4 weeks. (B) Representative images of mice and livers. (C) Oil Red O staining, HE staining, Masson staining, and Sirius Red staining of the liver. (D) ipGTT and ipITT curves and area under the curve (AUC) for each group of mice. (E) Liver weight and liver‐to‐body weight ratio. (F) Weight gain of mice. HFD+CCl4+AAV‐shCtrl vs iHFD+CCl4+AAV‐shCtrl, *p < 0.05, ***p < 0.001. HFD+CCl4+AAV‐shAhcy vs iHFD+CCl4+AAV‐shAhcy, ##*p* < 0.01, ###*p* < 0.001. (G) Fasting blood glucose. iHFD+CCl4+AAV‐shCtrl vs iHFD+CCl4+AAV‐shAhcy, *p < 0.05. (H) Liver tissue TG and TC levels in each group. (I) Serum TG and TC levels. (J) Serum ALT and AST levels. (K) The mRNA expression levels of hepatic fat formation indicators Srebf1, Fasn, Scd1, inflammatory indicators Il6, Tnfa, Il1b, and fibrosis indicators a‐SMA, Col1a1, Tgfb1. (L) The protein expression and quantitative analysis of Ahcy and Acot12 in primary cells of mouse liver. (M) The mRNA expression of Ahcy and Acot12 in primary cells of mouse liver. *p < 0.05, **p < 0.01, ***p < 0.001.

To investigate the role of IF in MASLD progression and the regulatory function of Ahcy, we established a MASH mouse model using a HFD combined with CCl_4_ administration, and performed Hnf4a overexpression or Ahcy knockdown via AAV delivery (Figure 8A). Compared with the HFD+CCl_4_ group, the iHFD+CCl_4_ group showed reduced hepatic lipid deposition and fibrosis, as indicated by Oil Red O, HE, and Masson staining (Figure 8B,C). These improvements were accompanied by enhanced glucose tolerance, decreased insulin resistance, lower liver and body weights, reduced fasting blood glucose, decreased plasma and hepatic TG and TC levels, lower plasma AST and ALT, and downregulated expression of lipogenic, inflammatory, and fibrotic genes (Figure 8D–K). In contrast, Ahcy knockdown in the iHFD+CCl_4_+AAV‐shAhcy group abolished the protective effects of IF, exacerbating steatosis and fibrosis (Figure 8C) and upregulating the gene expression of markers of liver injury, lipid accumulation, inflammation, and fibrosis (Figure 8H–K), despite unchanged body weight, liver weight, and liver‐to‐body weight ratio (Figure 8E,F). Metabolically, Ahcy knockdown also impaired glucose tolerance, elevated fasting blood glucose levels, and increased insulin resistance (Figure 8D,G). Similar exacerbations were observed in HFD+CCl_4_+AAV‐shAhcy mice relative to HFD+CCl_4_+AAV‐shCtrl controls (Figure 8B–K). In primary hepatocytes, Ahcy knockdown reduced Acot12 expression at both mRNA and protein levels (Figure 8L,M). These results collectively demonstrate that IF alleviates MASH progression, whereas Ahcy knockdown exacerbates MASH phenotypes and attenuates the protective effects of IF.

## 3 Discussion

IF demonstrates beneficial effects against MASLD [36, 37, 38]. However, the underlying mechanisms, especially those involving epigenetic regulation, remain incompletely understood. Our study reveals a comprehensive epigenetic pathway through which IF activates SE via the transcription factor Hnf4a, activating the methionine cycle‐related enzyme Ahcy. This activation leads to a protective transcriptional program via DNA methylation modulation, specifically regulating key lipid metabolism genes including Acot12, ultimately restoring hepatic metabolic homeostasis (Figure S9). The IF‐Hnf4a‐Ahcy axis represents a previously unrecognized mechanism connecting dietary cues to epigenetic reprogramming in metabolic disease.

SEs drive high‐level gene expression with cell and tissue specificity, and their involvement in metabolic diseases is increasingly recognized. In HFD‐induced MASLD liver tissues, SEs participate in regulating lipid metabolism genes [28, 29]. SE enhances transcription factor binding to target genes through chromatin conformational changes, promoting the expression of genes involved in hepatic lipid metabolism and inflammatory responses [26, 27]. Our findings indicate that SE‐regulated target genes are significantly enriched in hepatic lipid metabolism pathways. Knocking down Brd4 in mouse liver suppressed SE function, exacerbated MASLD progression, and substantially compromised the therapeutic efficacy of IF. These results suggest that SEs influence MASLD pathogenesis and IF‐mediated improvement by regulating genes involved in fatty acid metabolism and inflammation.

Our study identified Ahcy as a crucial SE‐driven effector in IF‐mediated MASLD amelioration. Integrated multi‐omics analyses demonstrated that Ahcy is governed by a prominent SE, with its activity suppressed by HFD and restored by IF. Functionally, liver‐specific Ahcy knockout exacerbated hepatic steatosis and attenuated IF benefits. Ahcy catalyzes S‐adenosylhomocysteine hydrolysis, thereby influencing methyltransferase activity and regulating gene methylation status in critical physiological processes [39, 40]. The relationship between Ahcy expression and fatty liver is well‐established. In mouse MASH models, downregulated hepatic Ahcy expression results in epigenetic dysregulation of lipid synthesis genes and impaired antioxidant gene transcription, which synergistically amplify oxidative stress and lipogenic signaling [41]. Zebrafish models show that reduced Ahcy activity elevates SAH levels, induces mitochondrial defects, increases lipogenic gene expression, and promotes hepatic inflammation and steatosis [14]. Patients with pathogenic Ahcy variants exhibit liver dysfunction [42, 43]. Consistently, our study confirms that liver‐specific Ahcy knockout enriches DEGs in lipid metabolism pathways and increases expression of lipogenic and inflammatory genes. These findings collectively demonstrate that SE‐associated Ahcy is critically involved in hepatic lipid metabolism and liver injury. The significant restoration of Ahcy expression in MASLD mice by IF suggests its potential as a key therapeutic target for MASLD intervention.

Hnf4a regulates transcription of genes essential for bile acid synthesis and lipid metabolism, maintaining lipid homeostasis [44]. Hnf4a knockout mice develop severe hepatomegaly and steatosis [45]. As a master regulator of hepatic lipid metabolism, Hnf4a binding occurs predominantly at active enhancer regions [46]. Our research confirms that Hnf4a drives the transcriptional activation of Ahcy during IF. IF alleviates HFD‐induced Hnf4a suppression, promoting coordinated recruitment of Hnf4a with Brd4 and H3K27ac to Ahcy regulatory regions. This transcriptional complex formation illustrates how SEs amplify transcriptional responses to environmental cues such as fasting, demonstrating that IF enhances Hnf4a expression to activate its bound SE and drive elevated Ahcy expression, thereby contributing to MASLD improvement.

A pivotal finding of this study concerns Ahcy‐mediated reshaping of the hepatic DNA methylation landscape. Although Ahcy deficiency reduces the SAM/SAH ratio, typically associated with reduced methylation, we observed promoter‐specific hypermethylation in lipid metabolism genes and hypomethylation in immune pathways, without global methylation alterations. This methylation patterning in Ahcy deficiency aligns with observations in Ahcy‐deficient patients. Studies of Ahcy deficiency cases have reported hypermethylation in leukocyte DNA [16]. Some patients show elevated global DNA methylation levels and imprinting gene hypermethylation [43], while others maintain normal global methylation despite significantly elevated SAH [43]. These observations from both our work and previous studies highlight the complexity of methylation regulation and indicate that Ahcy‐controlled methylation reprogramming exhibits remarkable locus specificity. The underlying mechanism may involve the relative insensitivity of DNA methyltransferases to SAH inhibition compared to non‐DNA methyltransferases [13]. Upon Ahcy knockout, the resultant SAH accumulation likely suppresses many SAH‐sensitive non‐DNA methyltransferases. This could redirect the available SAM pool toward the more resilient DNA methyltransferases, thereby driving locus‐specific hypermethylation at target genes, while leaving the global DNA methylation landscape largely unaltered. Notably, betaine supplementation, a methyl donor that globally corrects the SAM:SAH imbalance and ameliorates MASLD [47, 48], rescued the metabolic defect caused by Ahcy‐E3 deletion. This suggests that restoring the methyl donor pool can bypass the need for intact Ahcy‐driven epigenetic regulation to maintain hepatic lipid homeostasis.

Among Ahcy targets, Acot12 emerges as a key effector linking methylation changes to metabolic outcomes. Acot12 hydrolyzes acyl‐CoAs to regulate lipogenesis, and its deficiency leads to acetyl‐CoA accumulation, which increases hepatic de novo lipogenesis and cholesterol biosynthesis [34, 35, 49, 50]. The epigenetic silencing of Acot12 following Ahcy knockout provides a direct mechanism for the observed lipid accumulation. Despite SAH accumulation expected to broadly inhibit methyltransferase activity, liver‐specific Ahcy knockout in IF mice significantly increased Dnmt3b enrichment at the Acot12 promoter region without affecting Dnmt1. This suggests that localized Dnmt recruitment can override global methyltransferase suppression, and that methylation status at specific gene loci depends on Dnmt recruitment patterns. Consequently, Ahcy deletion regulates Acot12 promoter methylation through complex mechanisms requiring further investigation. It should be noted that the selection of Acot12 for further mechanistic validation in this study reflects a certain degree of research focus and subjective prioritization. In addition to Acot12, other hypermethylated and downregulated candidate genes, including Agmat, Agxt, Gamt, Apob, Apoc2, Pck1, and Pklr, may also be involved in the regulation of the observed phenotype. However, compared with Acot12, the functional links of these genes to the lipid deposition phenotype appear to be relatively less direct. Therefore, they were not subjected to further mechanistic validation in the present study and still warrant further investigation in future work.

This study has limitations that should be acknowledged. The absence of human sample validation limits the clinical applicability of our findings. Additionally, the precise molecular mechanisms governing the Ahcy‐regulated methylation network remain incompletely elucidated, warranting further research. Furthermore, although 5 µM JQ1‐PA produced the most pronounced inhibition of Ahcy in our experiments and has been commonly used in previous in vitro studies, potential non‐specific or off‐target effects at this concentration cannot be completely excluded.

In summary, we have defined a coherent IF‐Hnf4a‐Ahcy epigenetic pathway through which IF ameliorates MASLD via SE‐driven Ahcy activation. Our work demonstrates how dietary interventions can rewire the epigenome through SE remodeling and metabolite‐mediated methylation changes, positioning the Ahcy pathway as a potential therapeutic target for metabolic disease.

## 4 Methods

### 4.1 Animal Studies

8‐week‐old male C57BL/6 wild‐type mice and Ahcy flox/flox mice were purchased from Guangdong Zhiyuan Biomedical Technology Co., Ltd. and Cyagen Biosciences (Suzhou) Co., Ltd., respectively. After a one‐week acclimation period, all mice were housed in an SPF environment maintained at 23°C–24°C, a 12 h light/12 h dark cycle, and 60% ± 10% relative humidity. The volume for tail vein injection of the AAV virus was 100–200 µL. The injection dose was 2E+11‐ 5E+11 vg per mouse. All animal experiments were approved by the Experimental Animal Management Committee and Ethics Committee of our institution (Approval No. SDYY‐LH‐12‐2404‐010, Supplementary Information 3).

### 4.2 Animal Experiment 1: Effects of IF on HFD‐Induced MASLD Mice

C57BL/6 J mice were fed an HFD for 12 weeks to establish a MASLD mouse model. 8‐week‐old male C57BL/6J wild‐type mice were randomly assigned to four groups: CD (normal ad libitum diet group), iCD (normal IF diet group), HFD (high‐fat ad libitum diet group) (60% fat, D12492, Research Diets, USA), and iHFD (high‐fat IF diet group), n = 8. The IF groups followed an alternate‐day fasting protocol, with fasting or feeding starting daily at 9:00 AM. This involved feeding on one day and fasting on the next, in a cyclical pattern; on fasting days, the mice were transferred to new cages. Daily food intake was recorded, body weight was measured weekly (6‐day intervals), and fasting blood glucose was assessed biweekly. An intraperitoneal glucose tolerance test (ipGTT) and intraperitoneal insulin tolerance test (ipITT) were performed during the final two weeks, respectively. The total intervention period was 12 weeks. At the time of tissue collection, mice in the IF groups were allowed normal access to food until ZT12 (20:00) on their last feeding day, after which food was removed, and they were fasted for 12 h until ZT0 (08:00), when they were euthanized and tissues were collected. Although the control groups had been fed ad libitum throughout the study, on the day of tissue collection they were also subjected to food removal at ZT12 and a 12 h fast until ZT0, and were processed and sampled simultaneously with the intervention groups, ensuring that all animals were compared under the same circadian time point and nutritional status.

### 4.3 Animal Experiment 2: Role of Ahcy in IF‐Mediated Improvement of MASLD

8‐week‐old male Ahcy flox/flox mice were randomly assigned to the Ahcy^fl/fl^+HFD, Ahcy^LKO^+HFD, Ahcy^fl/fl^+iHFD, and Ahcy^LKO^+iHFD group, n = 8. After 8 weeks of dietary intervention, mice received tail vein injections of AAV8‐TBG‐Cre or control virus AAV8‐TBG‐Ctrl to establish liver‐specific Ahcy‐deficient or control groups, followed by 4 weeks of continued dietary model feeding. Parameters were measured as in Animal experiment 1 during the feeding period.

### 4.4 Animal Experiment 3: Effects of SEs on the Expression of Ahcy and IF

8‐week‐old male C57BL/6J wild‐type mice were randomly assigned to the following groups: CD+AAV‐Ctrl, CD+AAV‐shBrd4, HFD+AAV‐Ctrl, HFD+AAV‐shBrd4, iHFD+AAV‐Ctrl, and iHFD+AAV‐shBrd4, n = 10. After 8 weeks of dietary intervention according to the respective models, mice received tail vein injections of AAV8‐shBrd4 or control virus AAV8‐Ctrl to achieve liver‐specific knockdown/non‐knockdown of Brd4, followed by 4 weeks of continued dietary model feeding. Parameters were measured as in Animal experiment 1 during the feeding period.

### 4.5 Animal Experiment 4: Hnf4a Regulates Ahcy at the Transcriptional Level via SEs to Improve MASLD

8‐week‐old male C57BL/6J wild‐type mice were randomly assigned to the following groups: HFD+AAV‐Ctrl+AAV‐shCtrl, HFD+AAV‐Hnf4a+AAV‐shCtrl, HFD+AAV‐Hnf4a+AAV‐shAhcy, iHFD+AAV‐Ctrl+AAV‐shCtrl, iHFD+AAV‐Hnf4a+AAV‐shCtrl, and iHFD+AAV‐Hnf4a+AAV‐shAhcy group, n = 8. After 4 weeks of dietary intervention, liver‐specific overexpression/non‐overexpression of Hnf4a was achieved via tail vein injection of AAV8‐Hnf4a or control virus AAV8‐Ctrl. Animals continued their assigned diets for an additional 4 weeks. Liver‐specific knockdown/non‐knockdown of Ahcy was induced via tail vein injection of AAV8‐shAhcy or control virus AAV8‐shCtrl, followed by continued feeding on the respective dietary model for 4 weeks. Parameters were measured as in Animal experiment 1 during the feeding period.

### 4.6 Animal Experiment 5: Effects of Betaine Supplementation on Ahcy Deficiency‐Aggravated Hepatic Lipid Accumulation

8‐week‐old male C57BL/6J wild‐type mice were randomly assigned to the following groups: HFD+AAV‐shAhcy+NC, HFD+AAV‐shAhcy+Betaine, iHFD+AAV‐shAhcy+NC, and iHFD+AAV‐shAhcy+Betaine, n = 8. After 12 weeks of dietary intervention according to the respective models, liver‐specific knockdown of Ahcy was achieved via tail vein injection of AAV8‐shAhcy. The HFD+AAV‐shAhcy+NC and iHFD+AAV‐shAhcy+NC groups were provided with normal drinking water. The HFD+AAV‐shAhcy+Betaine and iHFD+AAV‐shAhcy+Betaine groups received drinking water supplemented with 1.5% (w/v) betaine (ST2209, Beyotime, China). Animals continued feeding on their respective diets for an additional 8 weeks. Parameters were measured as in Animal Experiment 1 during the feeding period.

### 4.7 Animal Experiment 6: Effects of IF and Ahcy on MASH Mice

8‐week‐old male C57BL/6J wild‐type mice were randomly assigned to the following groups: HFD+CCl4+AAV‐Ctrl, HFD+CCl4+AAV‐shAhcy, iHFD+CCl4+AAV‐Ctrl, and iHFD+CCl4+AAV‐shAhcy, n = 8. After 8 weeks of dietary intervention according to the respective models, liver‐specific overexpression of Hnf4a was achieved via tail vein injection of AAV8‐Hnf4a. Two weeks later, CCl4 was administered via intraperitoneal injection twice weekly. CCl4 was prepared as a 10% solution in olive oil and injected at a dose of 0.5–1 mL/kg until the intervention ended. Animals were maintained on the corresponding dietary model for 2 weeks. Liver‐specific knockdown/non‐knockdown of Ahcy was achieved via tail vein injection of AAV8‐shAhcy or control virus AAV8‐Ctrl. Animals were maintained on their corresponding diets for 4 weeks. Parameters were measured as in Animal experiment 1 during the feeding period. The aforementioned AAV vectors were purchased from Cyagen Biosciences (Suzhou) Co., Ltd.

At the end of all experiments, the mice were anesthetized with 1.25% 2,2,2‐tribromoethanol (30 µl/g) by intraperitoneal injection and then euthanized. The 2,2,2‐Tribromoethanol was purchased from Macklin (catalog number T708333).

### 4.8 ipGTT and ipITT

For ipGTT, mice were given glucose (1 g/kg body weight) by intraperitoneal injection after fasting overnight. The blood glucose level of the tail vein was measured at 0, 15, 30, 60, 90, and 120 min after glucose load. For ipITT, mice were given insulin (0.75 U/kg body weight) by intraperitoneal injection after fasting for 4 h. The blood glucose level in the tail vein was measured at 0, 15, 30, 60, 90, and 120 min after insulin load.

### 4.9 Serum Triglycerides (TG), Total Cholesterol (TC), Aspartate Aminotransferase (AST), Alanine Aminotransferase (ALT), and Hepatic TG, TC, SAH, and SAM Content Detection

TG, TC, AST, and ALT assay kits were purchased from Nanjing Jiancheng Bioengineering Institute. Each parameter was measured according to the respective kit instructions. According to the manufacturer's agreement, the contents of SAH and SAM in liver were detected by ELISA kits (WLCSJZF20904, WLCSJZF28426, Lunchangshuo Biotechnology, China).

### 4.10 HE Staining of Liver Tissue

Mouse liver tissue was fixed in 4% paraformaldehyde solution. After dehydration, clearing, wax immersion, embedding, and sectioning, the tissue sections underwent xylene dewaxing. Sections were stained with hematoxylin and 0.5% eosin solution, then dehydrated and mounted with neutral resin. Stained tissue sections were digitally acquired using a whole‐slide pathological scanner (Servicebio, LG‐S80). All scans were uniformly performed with a 20× objective. The final magnification of section images used for subsequent analysis was 20×.

### 4.11 Oil Red O Staining of Liver Tissue

Fresh liver tissue was rapidly frozen and sectioned into 5–10 µm cryosections, air‐dried at room temperature, and lightly washed with 70% ethanol. Sections were then immersed in Oil Red O staining solution for 10–15 min. After washing with distilled water, the nuclei were stained with hematoxylin for 3–5 min. Excess stain was rinsed with tap water, allowing the nuclei to turn blue. Finally, sections were sealed with glycerol gelatin.

### 4.12 Masson Staining of Liver Tissue

Liver sections were fixed in 4% neutral formaldehyde, embedded in paraffin, sectioned serially at 4 µm, and then dewaxed in water. The nuclei were stained with Weigert hematoxylin. Subsequently, the cytoplasm and muscle fibers were stained with ponceau acid fuchsin solution. After differentiation with 1% phosphomolybdic acid, collagen was stained with aniline blue, followed by a quick wash with 1% glacial acetic acid. The liver parenchyma (red) and fibrotic area (blue) can be clearly distinguished under a light microscope after graded ethanol dehydration, clearing in xylene, and sealing with neutral resin.

### 4.13 Sirius Red Staining of Liver Tissue

The fixed liver tissue was processed into paraffin sections. After dewaxing in water, the sections were stained with Sirius red saturated picric acid solution for approximately 1 h, followed by rinsing with running water to remove floating color. Subsequently, the sections were dehydrated through a graded ethanol series, cleared with xylene, and finally mounted with neutral balsam. In the staining results, collagen fibers appeared red or orange‐red.

### 4.14 Immunohistochemistry

Paraffin‐embedded liver tissue sections were baked at 60°C, dewaxed with xylene, and rehydrated with graded ethanol. Endogenous peroxidase was blocked with 0.3% H_2_O_2_ in methanol. Antigen retrieval was performed at high temperature for 20 min using sodium citrate buffer (pH 6.0). Then, the sections were blocked with 10% goat serum at room temperature for 30 min. The sections were incubated overnight at 4°C with primary antibody. After washing with PBS, the sections were incubated with HRP‐labeled secondary antibody at 37°C for 30 min. DAB was used to develop color, and hematoxylin was used to stain the nucleus. After differentiation with hydrochloric acid ethanol and bluing with ammonia water, the sections were dehydrated, cleared, and sealed with neutral resin.

### 4.15 Cell Culture and Treatment

AML12 (alpha mouse liver‐12) hepatocytes were purchased from the cell bank of Sevier Biotechnology Co., Ltd. These cells were cultured in DMEM/F12 medium supplemented with 10% fetal bovine serum (FBS), 1% penicillin‐streptomycin (P/S), 1% ITS liquid culture supplement, and 40 ng/mL dexamethasone. 293T cells (HEK‐293T) were purchased from Cyagen Biosciences (Suzhou) Co., Ltd. They were cultured in DMEM containing 10% FBS and 1% P/S. All cells were cultured at 37°C in a 5% CO_2_ incubator. siRNA and plasmids were transfected into cells using Lipo2000. siRNA sequences are summarized in Table S1.

### 4.16 RT‐qPCR

Total RNA was extracted from cells or tissues with TRIzol reagent. After measuring RNA concentration and purity, the RNA was reverse transcribed into cDNA using a reverse transcription kit (R222, Vazyme, China). Detection was performed using SYBR qPCR Master Mix (Q71, Vazyme, China) on a real‐time quantitative PCR system. The internal reference gene was β‐actin. Relative expression levels of target genes were calculated using the 2^(‐ΔΔCt) method. Primer sequences are summarized in Table S2.

### 4.17 Western Blotting

Cells or mouse liver tissues were lysed using a mixture containing RIPA, protease inhibitor, and phosphatase inhibitor mixed at a ratio of 100:1:1. The protein concentration of the lysate was quantified using a BCA protein assay kit. The protein supernatant was mixed with 5×loading buffer, and then the mixture was placed in a metal bath at 95–100°C for 10 min. 30 µg of total protein were loaded for SDS‐PAGE, and the separated protein was transferred to the PVDF membrane. The membrane was blocked at room temperature for 1 h, followed by incubation with the corresponding primary antibody (1:1000) overnight at 4°C. After incubation, the membrane was washed with TBST three times. The secondary antibody (1:10,000) was then added and incubated for 1 h at room temperature, after which the membrane was washed again. The multifunctional molecular imaging system (Alliance Q9) was used for imaging. The antibodies used are summarized in Table S3.

### 4.18 Luciferase Reporter Gene Assays

293T cells were seeded into 96‐well plates at an appropriate density. The corresponding luciferase reporter plasmid or siRNA, along with the pRL‐luciferase plasmid, were co‐transfected with Lipo2000 reagent. 6 h after transfection, the medium was replaced with fresh medium. Samples were collected for detection 48 h after transfection. The Dual‐GLO Luciferase Assay System (E2920, Promega, USA) was used to measure luciferase activity. Dual‐Glo Luciferase Reagent and Dual‐Glo Stop & Glo Reagent were used to detect the luminescence of firefly luciferase and Renilla luciferase, respectively. Data were collected by Victor Nivo 3F (Revvity, USA).

### 4.19 Methylation‐Specific PCR (MSP)

A genomic DNA extraction kit (A0624A, TIANGEN, China) was used to extract the DNA of AML12 cells after intervention. The unmethylated cytosine was converted into uracil using a DNA bisulfite transformation kit (G3639, Servicebio, China). Subsequently, PCR amplification was performed using methylated and unmethylated primers, and the products were electrophoresed on agarose gel. Methylated and unmethylated primers of Acot12 are listed in Table S4.

### 4.20 ChIP‐qPCR

Protein bound to intracellular DNA was crosslinked with formaldehyde, and then chromatin was fragmented into short segments by ultrasound. Protein‐DNA complexes were immunoprecipitated with specific antibody and Protein A/G magnetic beads at 4°C. After cross‐linking removal, digestion with proteinase K, and purification, DNA fragments bound to the target protein were obtained, and qPCR was performed using specific primers. The qPCR process and data processing are the same as RT‐qPCR. The ChIP‐qPCR primers are listed in Table S2. The antibodies used are summarized in Table S3.

### 4.21 CRISPR‐Cas9‐Mediated Gene Disruption

Knockout of the E3 region in AML12 cells was mediated by the CRISPR‐Cas9 system. Two specific gRNA sequences, TGGAGAATGGTGGAATCTGG‐TGG and TAATTATATGATCCCCAGCG‐AGG targeting E3, were transferred into cells by electroporation. After electroporation, monoclonal cells were selected and their genotypes confirmed by PCR and sequencing. Homozygous AML12 cells with knockout of E3 (E3‐KO) were obtained.

### 4.22 RNA‐Seq

Total RNA was extracted using Trizol reagent according to the manufacturer's protocol. RNA purity, concentration, and integrity were assessed using a NanoDrop spectrophotometer (Thermo Scientific) and an Agilent Bioanalyzer 2100 (Agilent Technologies, Santa Clara, CA, USA). Sequencing libraries were constructed on the Illumina platform with standard protocols. Sequencing data were filtered using the fastp (https://github.com/OpenGene/fastp) software. Reference genome alignment was performed with HISAT2 (v2.0.5). Differential analysis was carried out using the DESeq2 R package. DEGs were screened based on |log2FC| > 1 and FDR<0.05. Visualization of DEGs was performed using volcano plots and heatmaps. Enrichment analyses for KEGG, GO, and GSEA were conducted using the clusterProfiler R package.

### 4.23 Cut and Run

The experiment was conducted by Guangzhou Epigenomics Technology Co., Ltd. (Guangzhou, China) according to the following protocol. Fresh liver tissue was collected from mice in the CD, iCD, HFD, and iHFD groups (n = 3). Liver tissue was lysed and digested into cells, which were then bound to ConA‐coated magnetic beads. H3K27ac antibody was added, and the mixture was incubated overnight at 4°C. Following antibody incubation, ConA beads were resuspended in a solution containing pA/G‐MNase fusion protein. After pA/G‐MNase incubation, the cell/magnetic bead mixture underwent multiple washes. Calcium ions were added to activate pA/G‐MNase bound to the target protein, enabling cleavage of the DNA bound to the target protein. After cleavage, the reaction stop solution was added, and the mixture was incubated at 37°C for 30 min. The DNA bound to the target protein was obtained by magnetic frame adsorption, and then purified and recovered. The library was established and sequenced by the Illumina platform. At the same time, the corresponding samples were sequenced for transcriptome, and the sequencing process was the same as above.

### 4.24 RRBS

The experiment was conducted by Shenzhen Aisigen Technology Co., Ltd. (Shenzhen, China) according to the following protocol. Fresh liver tissue was collected from two groups of mice: Ahcy^fl/fl^+HFD and Ahcy^LKO^+HFD (n = 3). Genomic DNA was extracted from mouse livers. The concentration of DNA was determined. The integrity and purity of DNA were assessed by agarose gel electrophoresis. The digested DNA fragments underwent end repair and were added with an A tail. Then, 40–220 bp DNA fragments were treated with bisulfite. PCR amplification was carried out to construct the DNA library. After the library passed quality control, the libraries were sequenced using Illumina HiSeq. After obtaining the raw data, the reference genome was compared by bioinformatics analysis. Methylated cytosine (mC) loci were defined, and the methylation level was calculated. Differential methylation regions (DMRs) were identified, and the genes related to DMRs were annotated. GO and KEGG were used for further functional enrichment analysis. At the same time, the corresponding samples were sequenced for the transcriptome, and the sequencing process was the same as above.

### 4.25 Statistical Analysis

The results are represented as mean ± standard error of the mean (SEM). GraphPad Prism 8.0 was used for statistical significance analysis and visualization. Two‐tailed Student's *t*‐test was used for comparison between the two groups, and one‐way analysis of variance (ANOVA) followed by Tukey's multiple comparison was used for comparison between multiple groups. Statistical significance was defined as *p* < 0.05.

## Author Contributions

**Huafeng Chen**: conceptualization, methodology, software, data curation, investigation, validation, visualization, writing – original draft, writing – review and editing. **Xiaojie Deng**: validation, investigation. **Hua Liang**: project administration, writing – review and editing, conceptualization, methodology, supervision, funding acquisition, resources. **Fen Xu**: project administration, writing – review and editing, funding acquisition. **Wenqiang Xie**: validation. **Jie Shen**: project administration, writing – review and editing, funding acquisition. **Shilin Zhang**: data curation, investigation.

## Conflicts of Interest

The authors declare no conflict of interest.

## Supporting information




**Supporting File 1**: advs76826‐sup‐0001‐SuppMat.docx.

**Supporting File 2**: advs76826‐sup‐0002‐FigureS1‐S9.zip.

**Supporting File 3**: advs76826‐sup‐0003‐DataFile.pdf.

## Data Availability

The public datasets used in this paper are sourced from the GEO database (Accession number: GSE118007 and GSE226171) and the 3Dgenome browser (Accession number: Liver_ZT_10_anti‐CTCF_ChIA‐PET and Liver_ZT_22_anti‐CTCF_ChIA‐PET). The additional data that support the findings of this study are available from the corresponding author upon reasonable request.
