# Supplementary material for: Ahcy Acts as an Effector of Hnf4a‐Driven Super‐Enhancer Activation to Alleviate MASLD During Intermittent Fasting
Source: Adv Sci (Weinh). 2026 Jul 27;13(59):e76826. doi: 10.1002/advs.76826 (PMC13403733; doi:10.1002/advs.76826)
Supplement: Supplementary file 1 [file ADVS-13-e76826-s001.pdf]

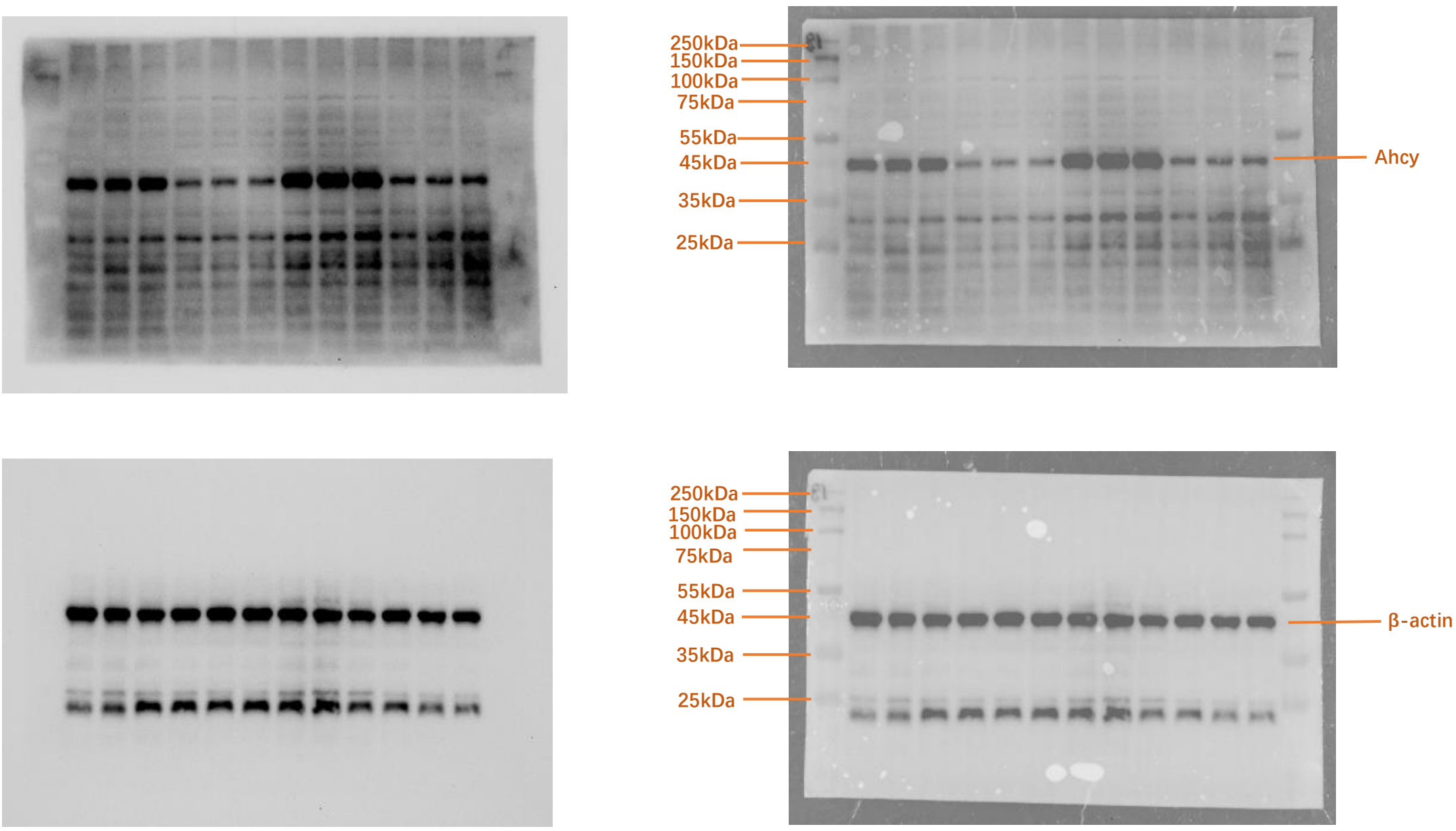

Extended Data Figure 1. The original gels for Western blot in Figure 2K. The left figure is the chemiluminescence image, and the right figure is the merged image.

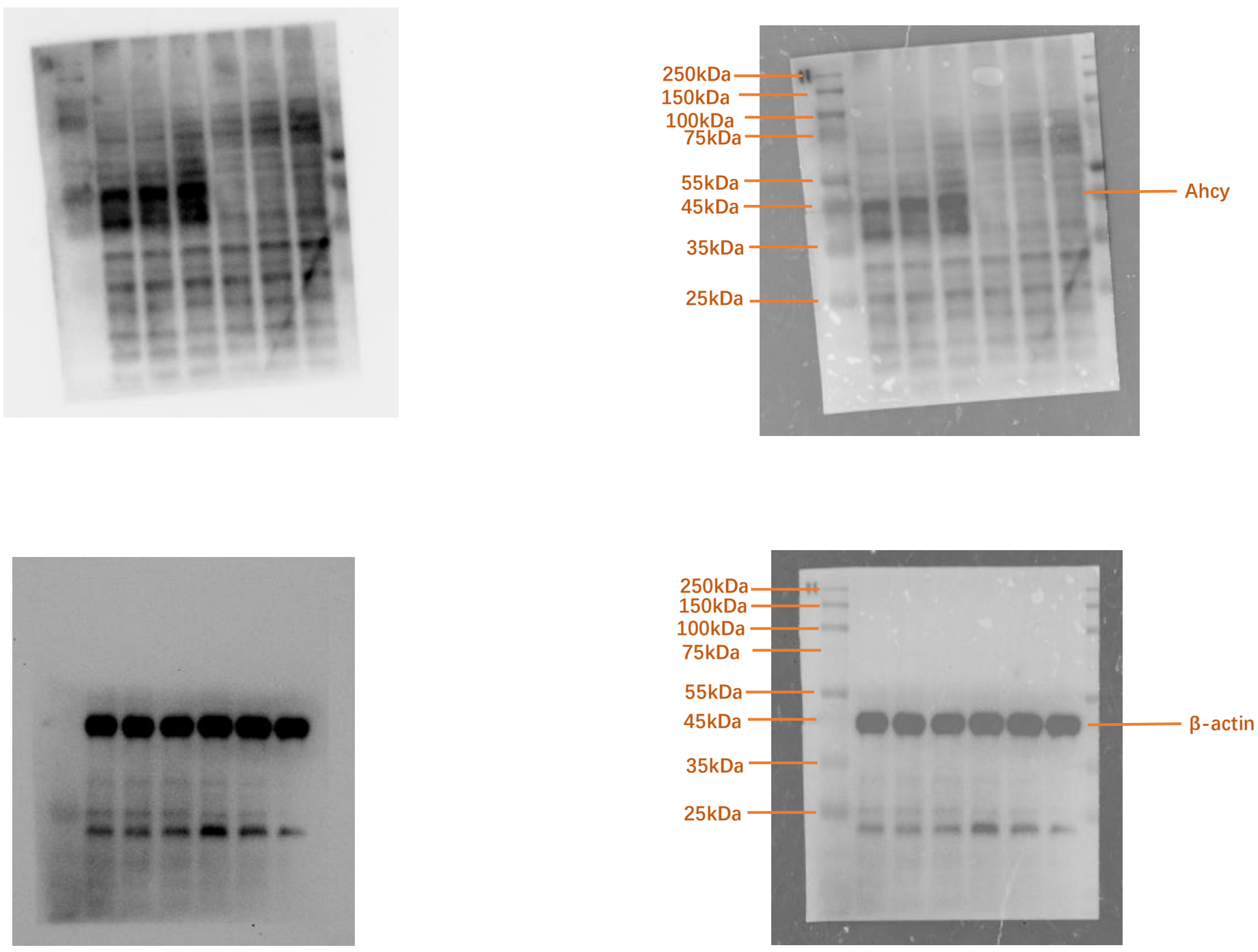

Extended Data Figure 2. The original gels for Western blot in Figure 2P. The left figure is the chemiluminescence image, and the right figure is the merged image.

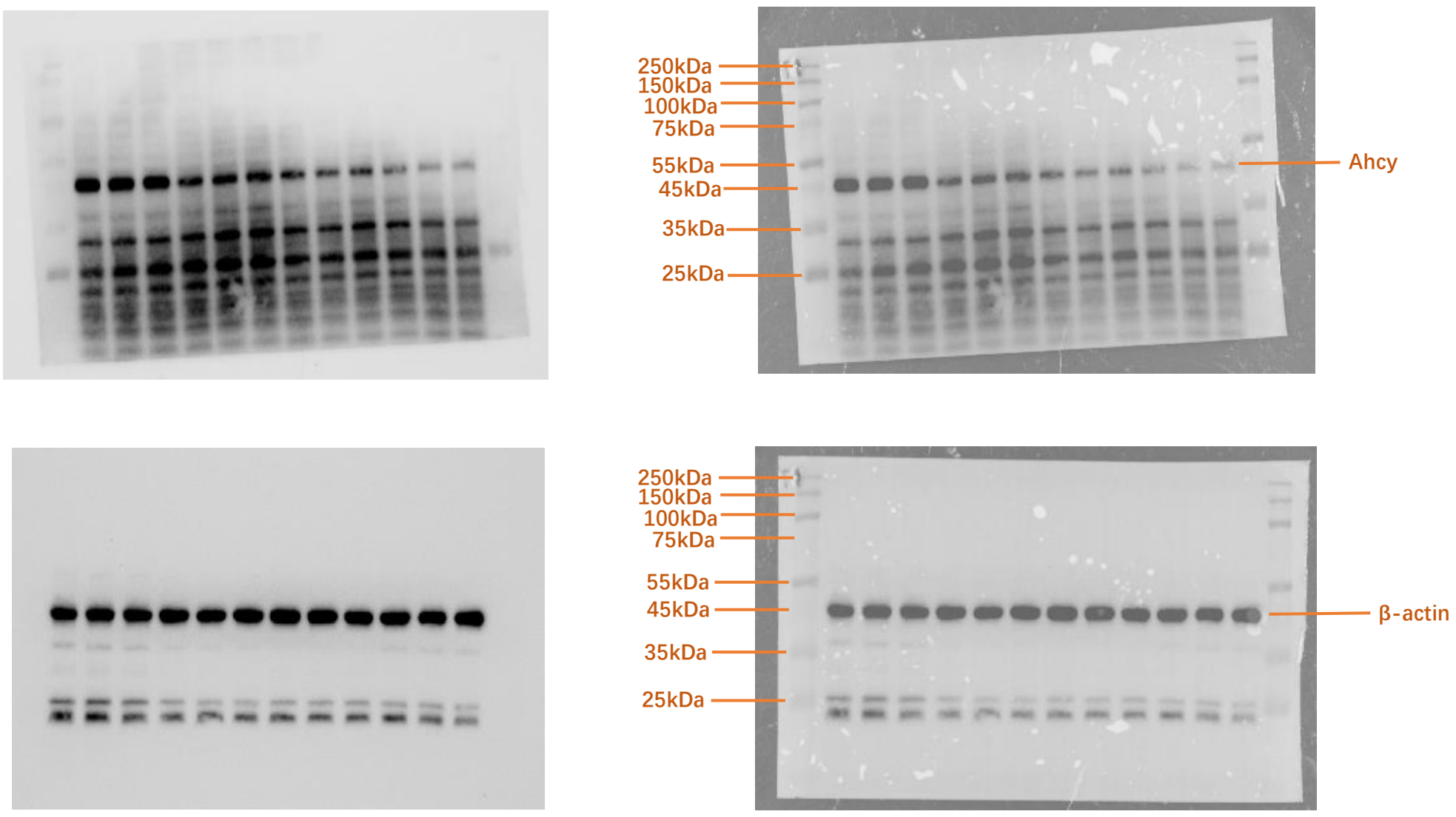

Extended Data Figure 3. The original gels for Western blot in Figure S2J. The left figure is the chemiluminescence image, and the right figure is the merged image.

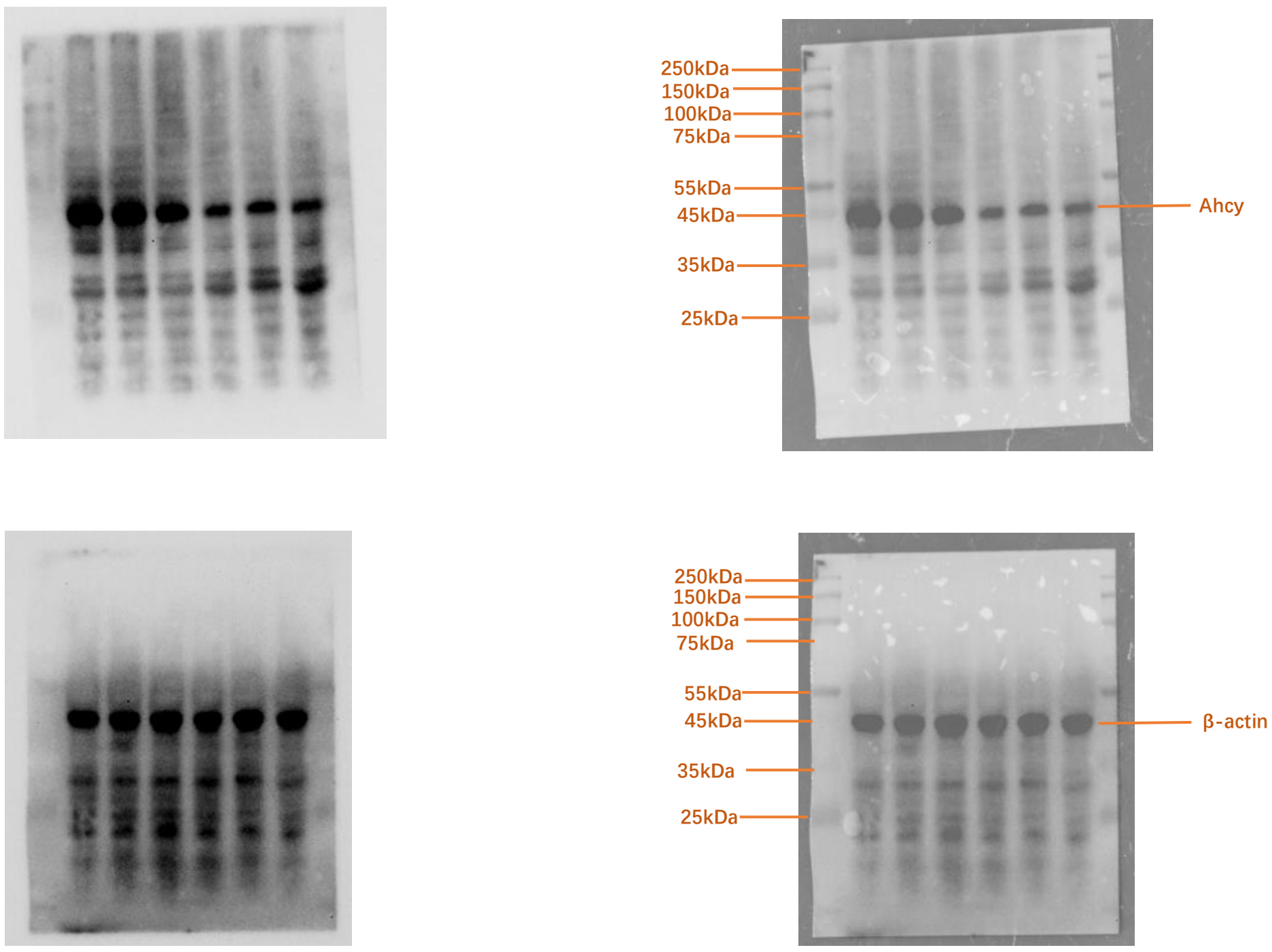

Extended Data Figure 4. The original gels for Western blot in Figure 3D. The left figure is the chemiluminescence image, and the right figure is the merged image.

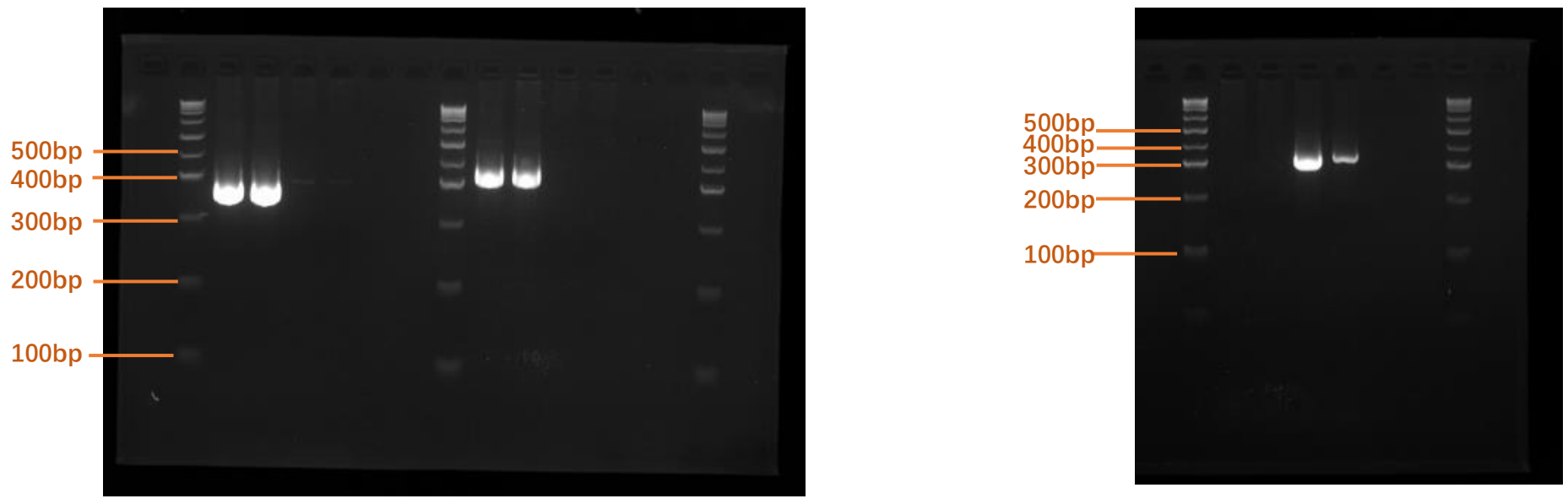

Extended Data Figure 5. The original gels of Figure S3C.

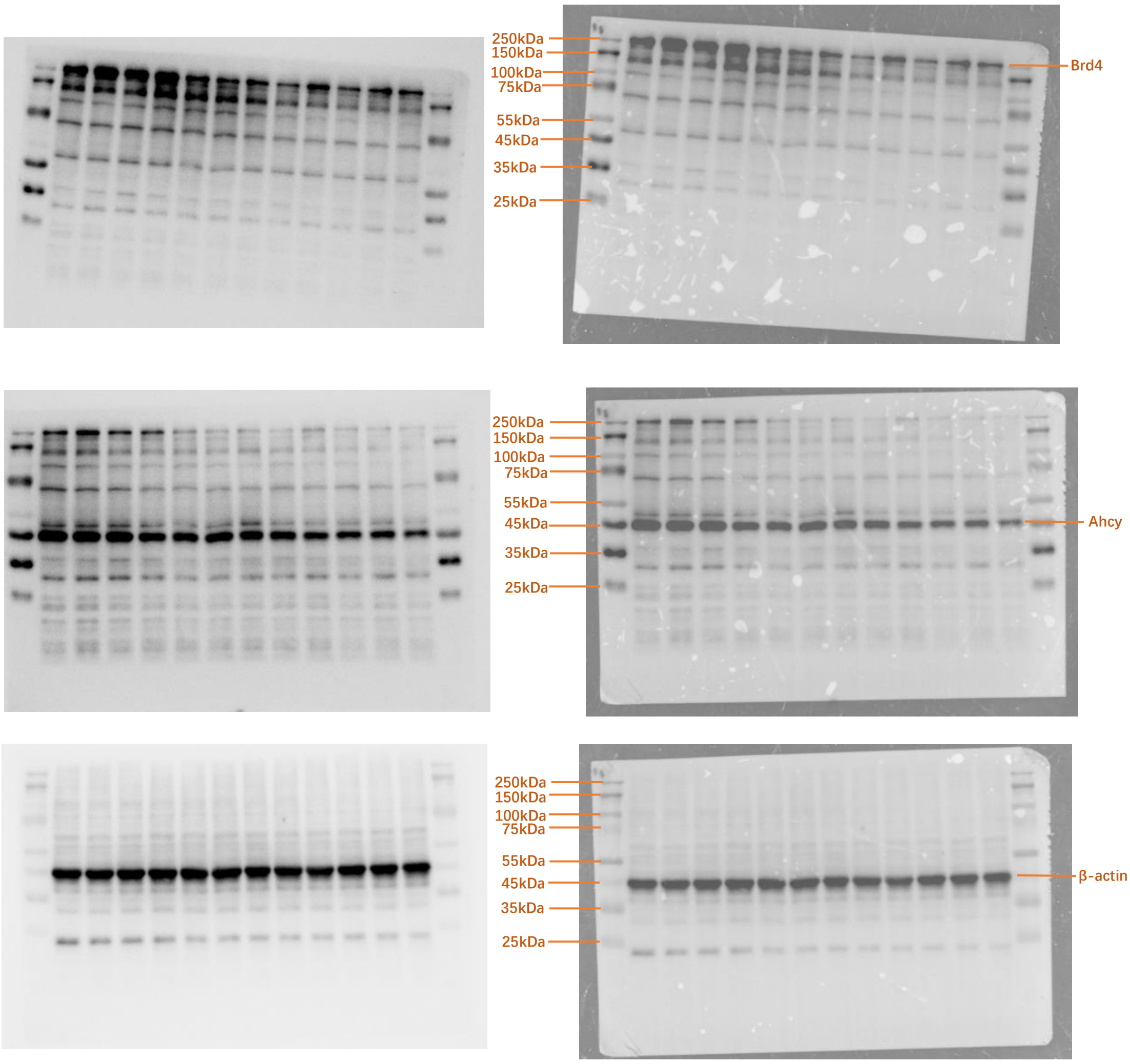

Extended Data Figure 6. The original gels for Western blot in Figure 4A. The left figure is the chemiluminescence image, and the right figure is the merged image.

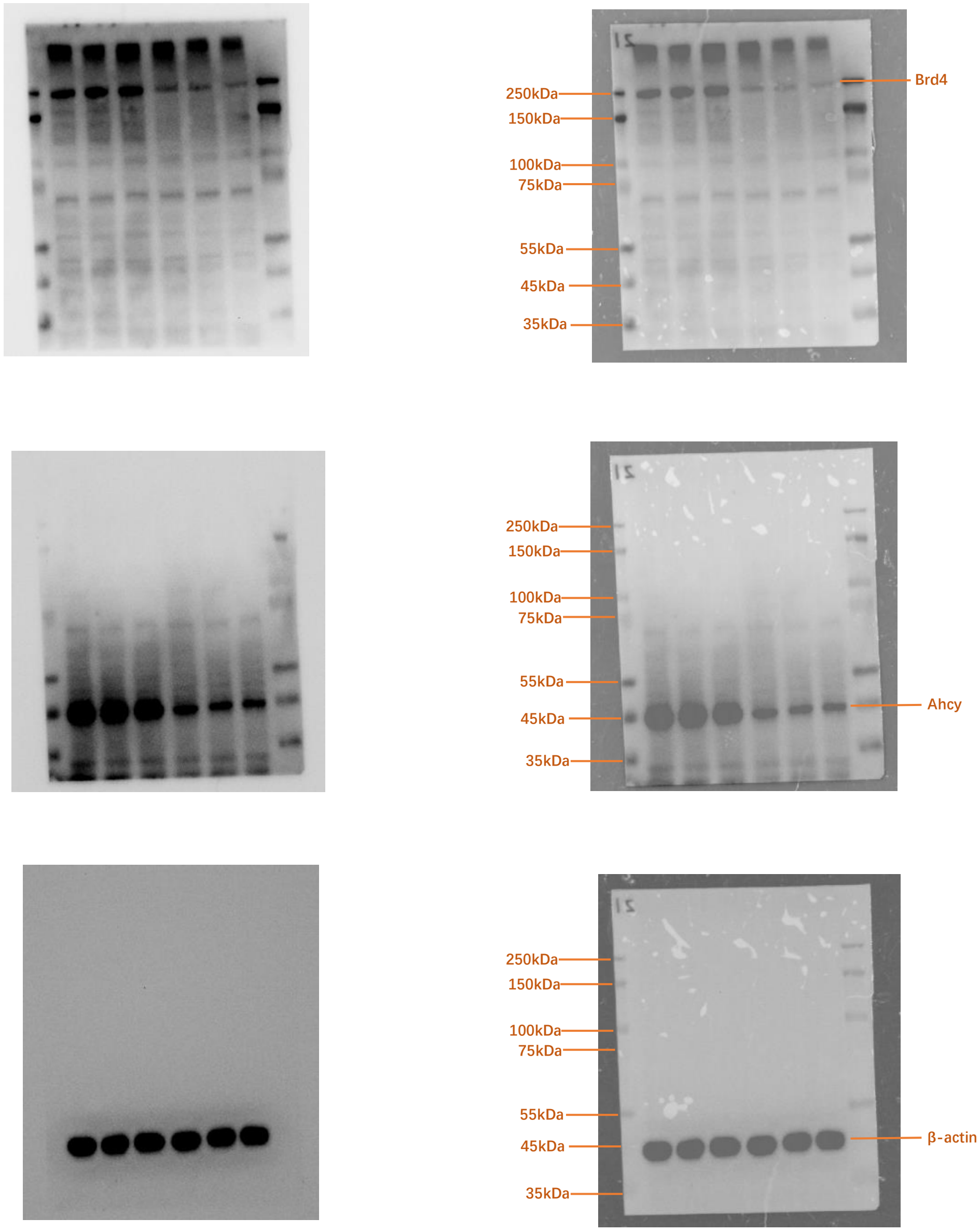

Extended Data Figure 7. The original gels for Western blot in Figure 4D. The left figure is the chemiluminescence image, and the right figure is the merged image.

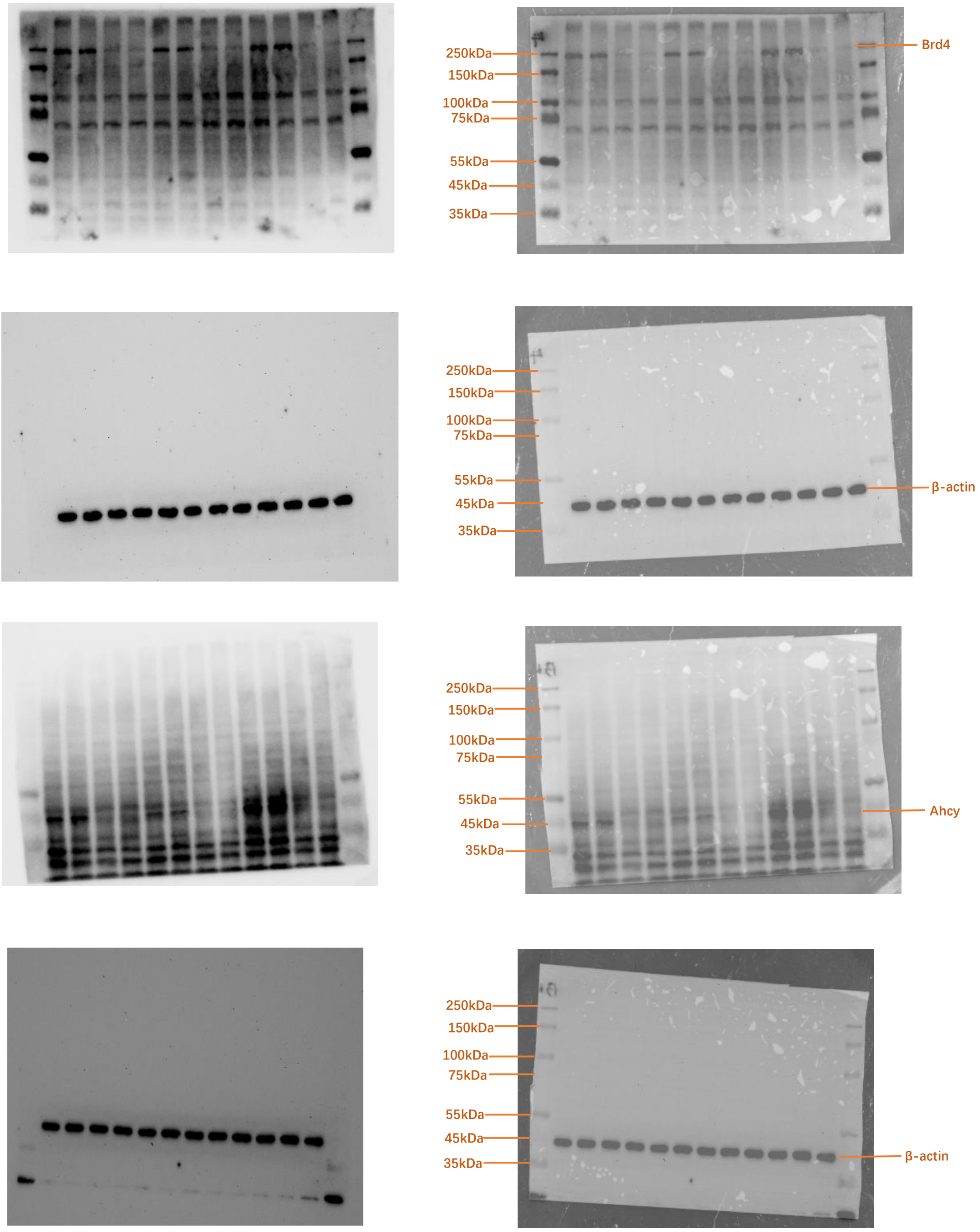

Extended Data Figure 8. The original gels for Western blot in Figure 4P. The left figure is the chemiluminescence image, and the right figure is the merged image.

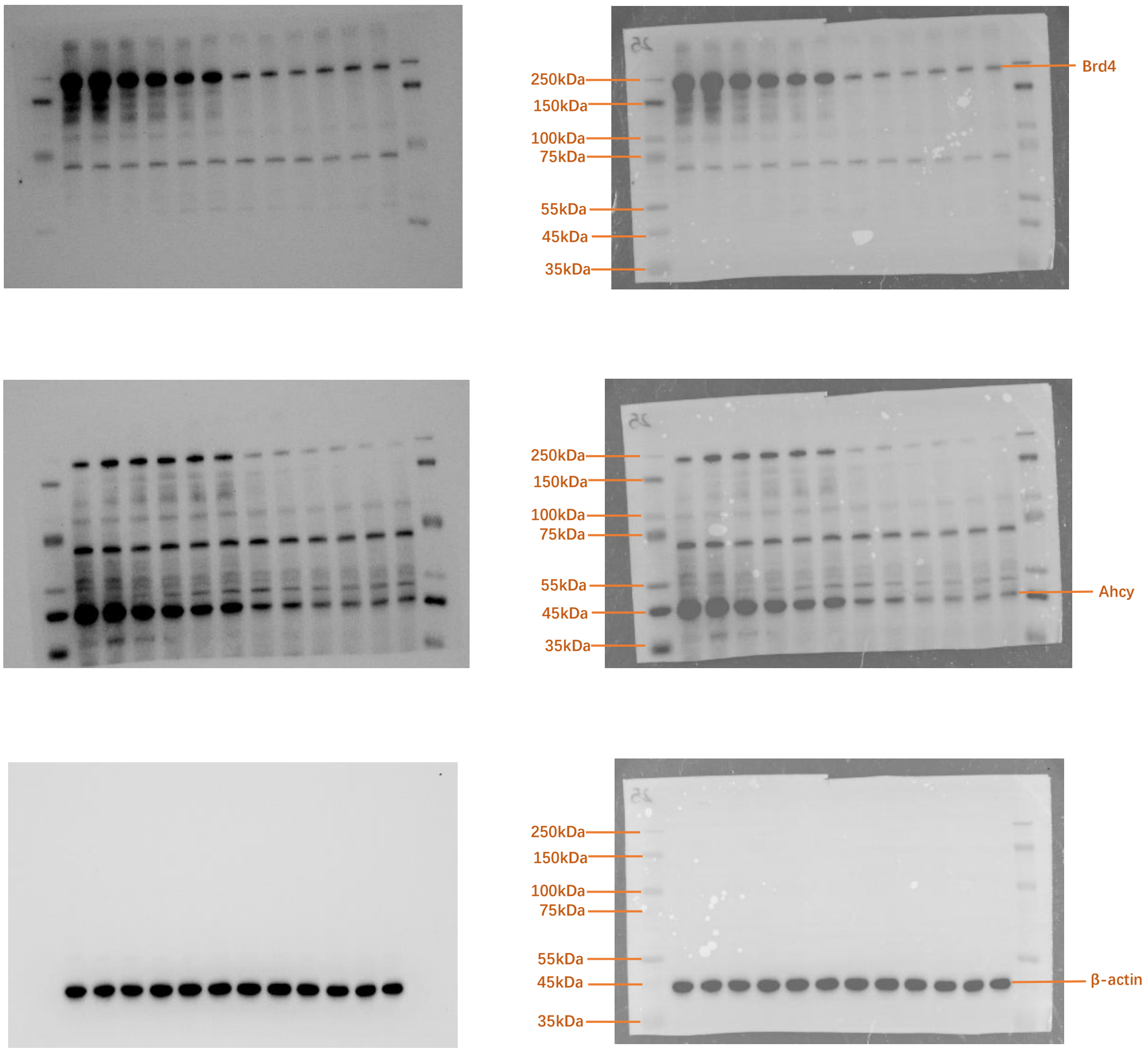

Extended Data Figure 9. The original gels for Western blot in Figure S4A. The left figure is the chemiluminescence image, and the right figure is the merged image.

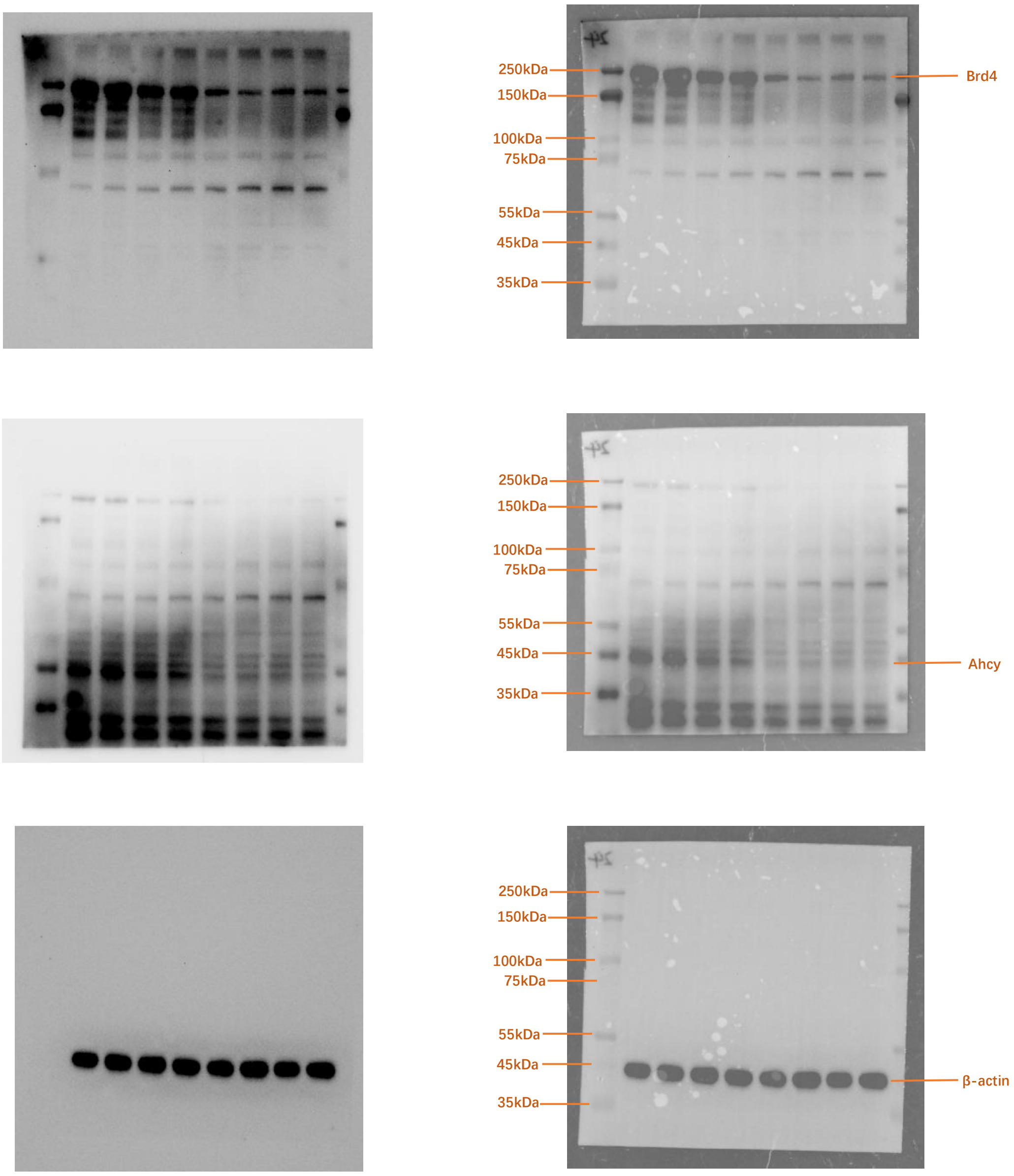

Extended Data Figure 10. The original gels for Western blot in Figure S4C. The left figure is the chemiluminescence image, and the right figure is the merged image.

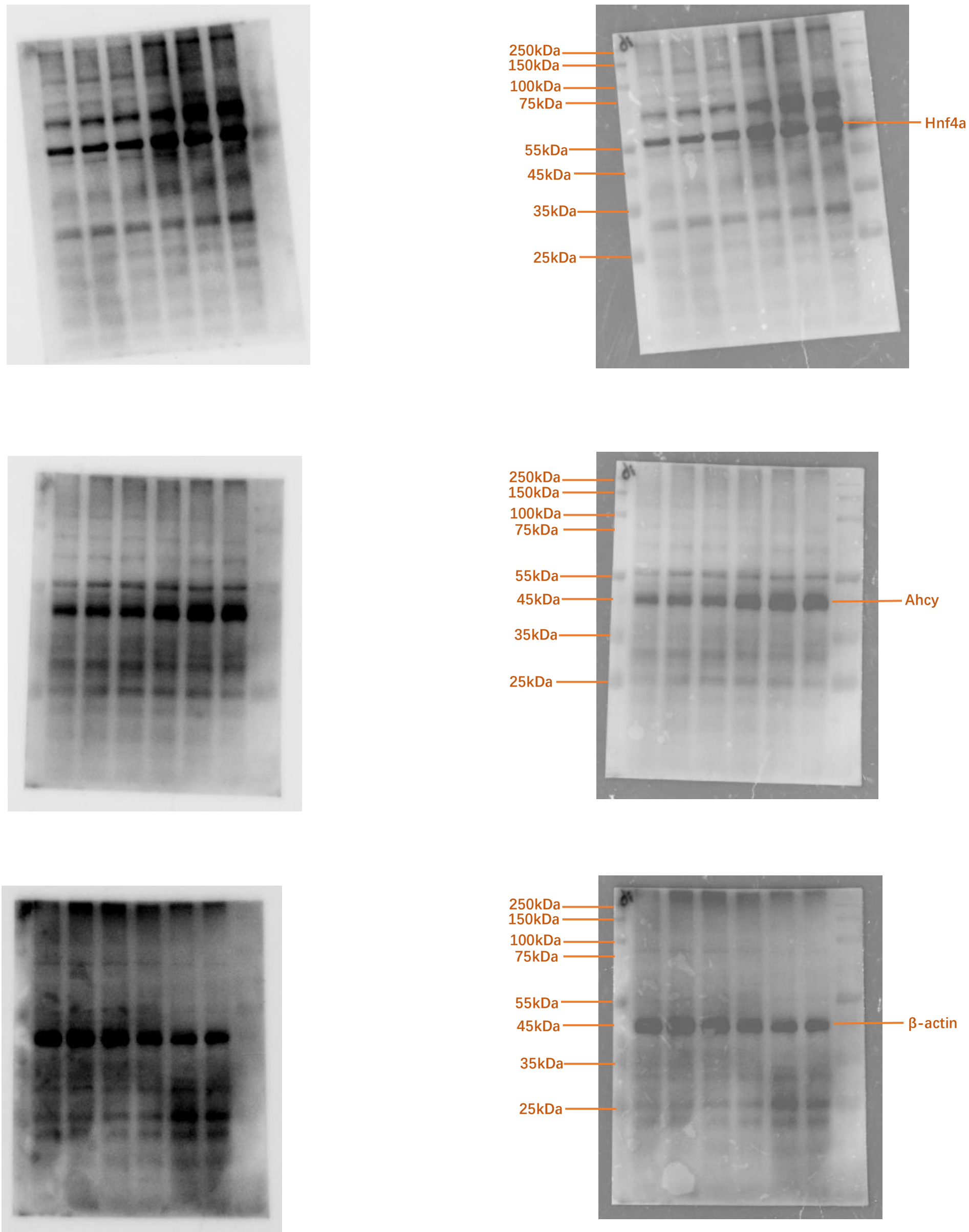

Extended Data Figure 11. The original gels for Western blot in Figure 5C. The left figure is the chemiluminescence image, and the right figure is the merged image.

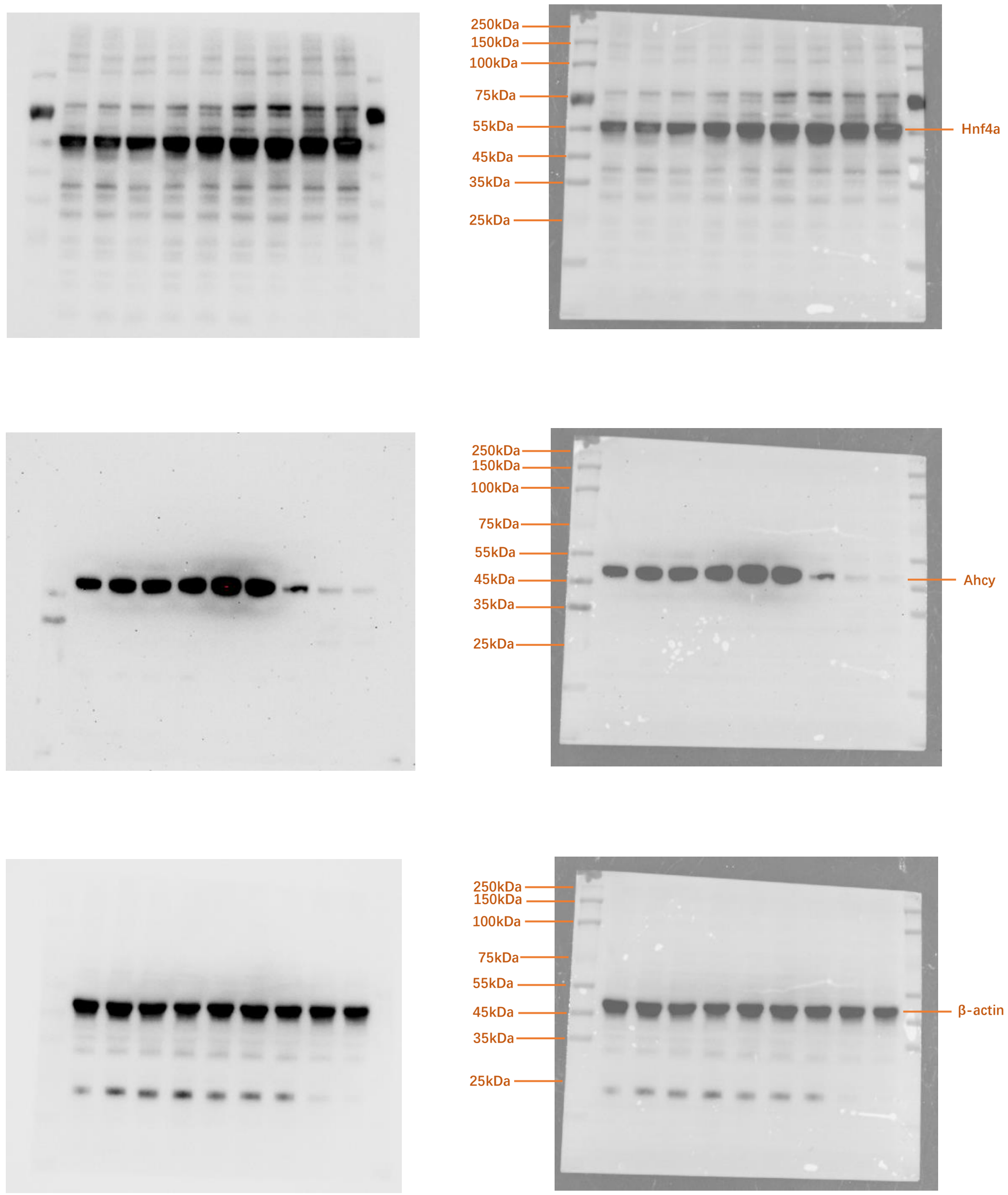

Extended Data Figure 12. The original gels for Western blot in Figure 5O. The left figure is the chemiluminescence image, and the right figure is the merged image.

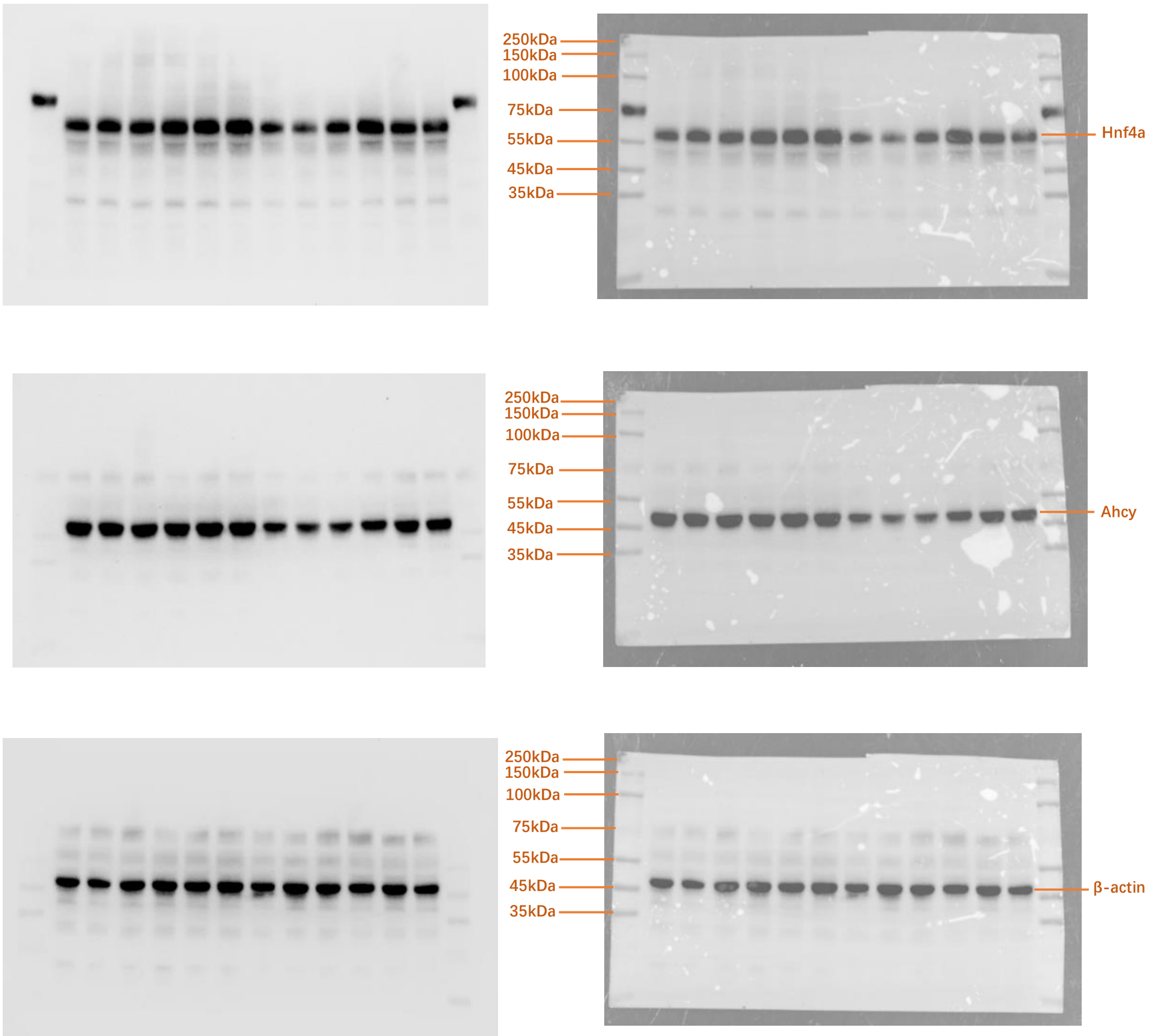

Extended Data Figure 13. The original gels for Western blot in Figure 6B. The left figure is the chemiluminescence image, and the right figure is the merged image.

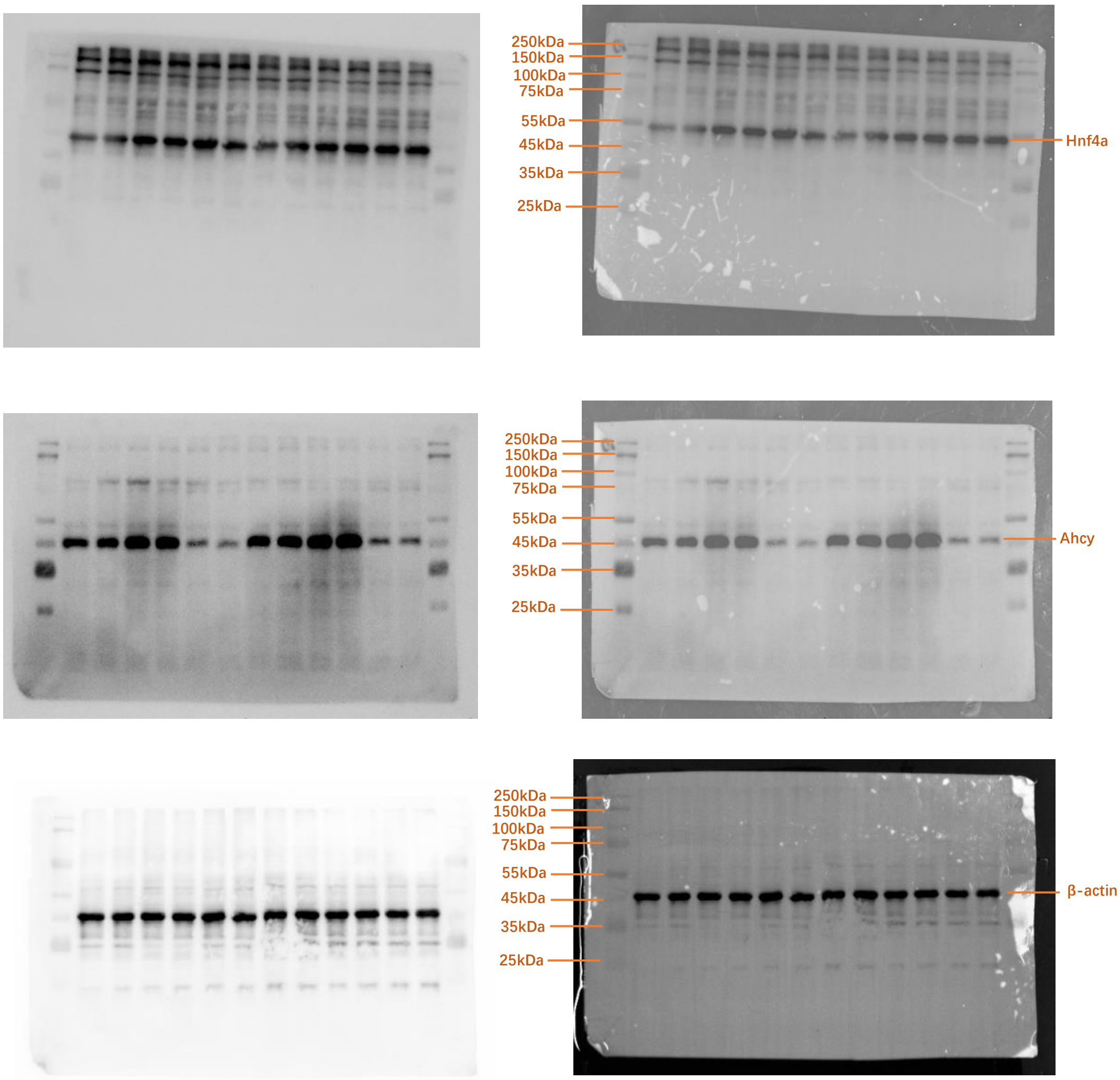

Extended Data Figure 14. The original gels for Western blot in Figure 6J. The left figure is the chemiluminescence image, and the right figure is the merged image.

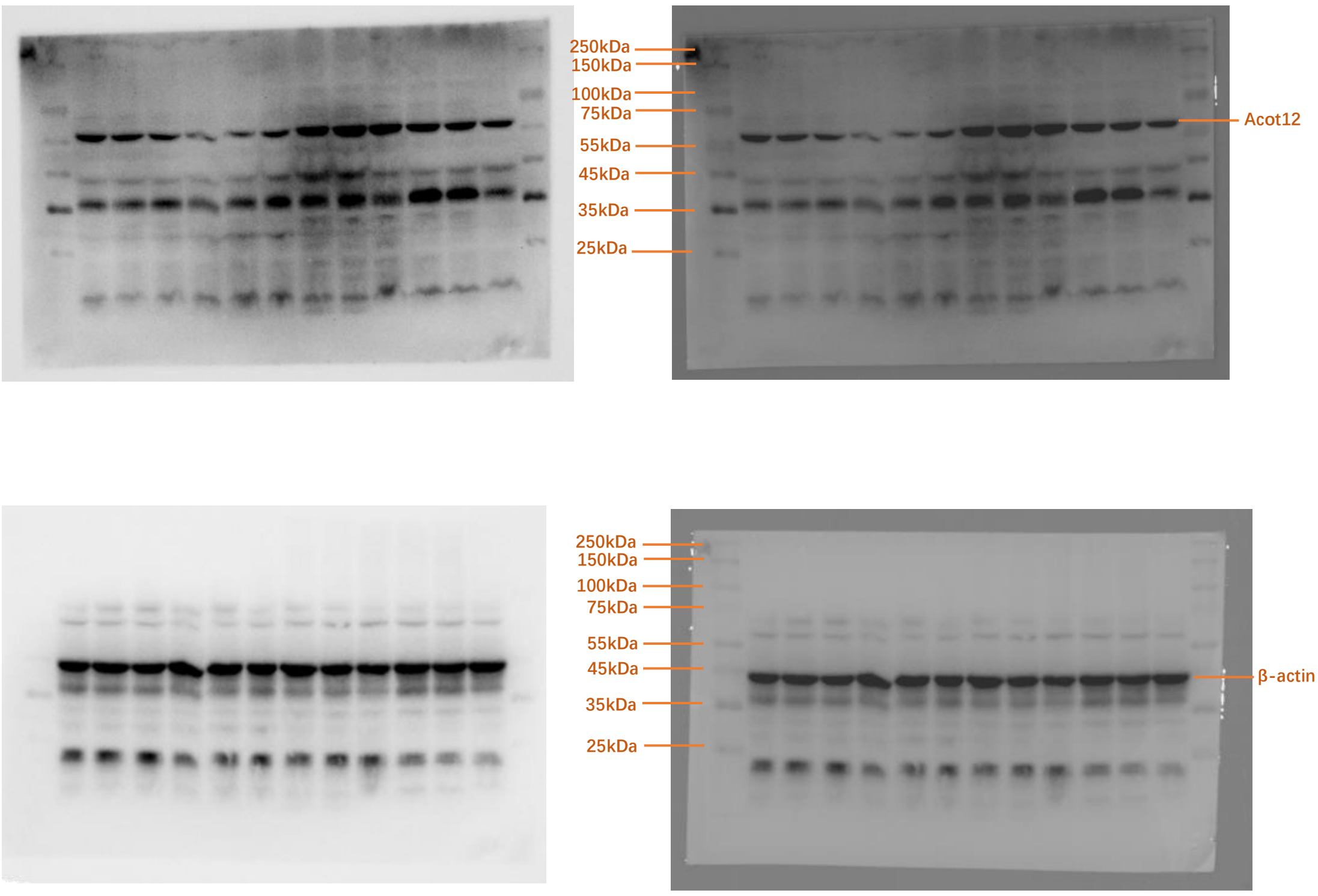

Extended Data Figure 15. The original gels for Western blot in Figure 7F. The left figure is the chemiluminescence image, and the right figure is the merged image.

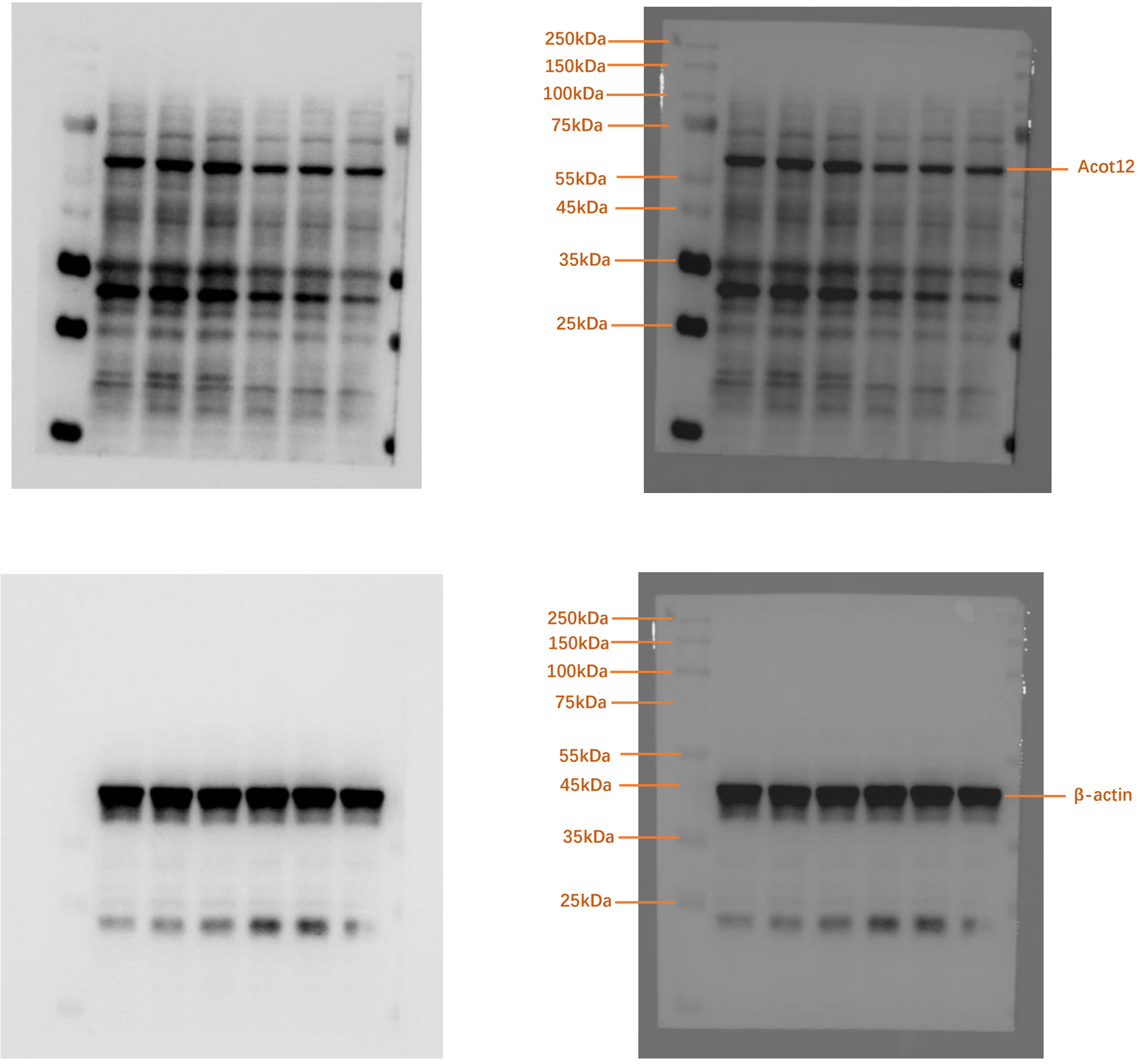

Extended Data Figure 16. The original gels for Western blot in Figure 7H. The left figure is the chemiluminescence image, and the right figure is the merged image.

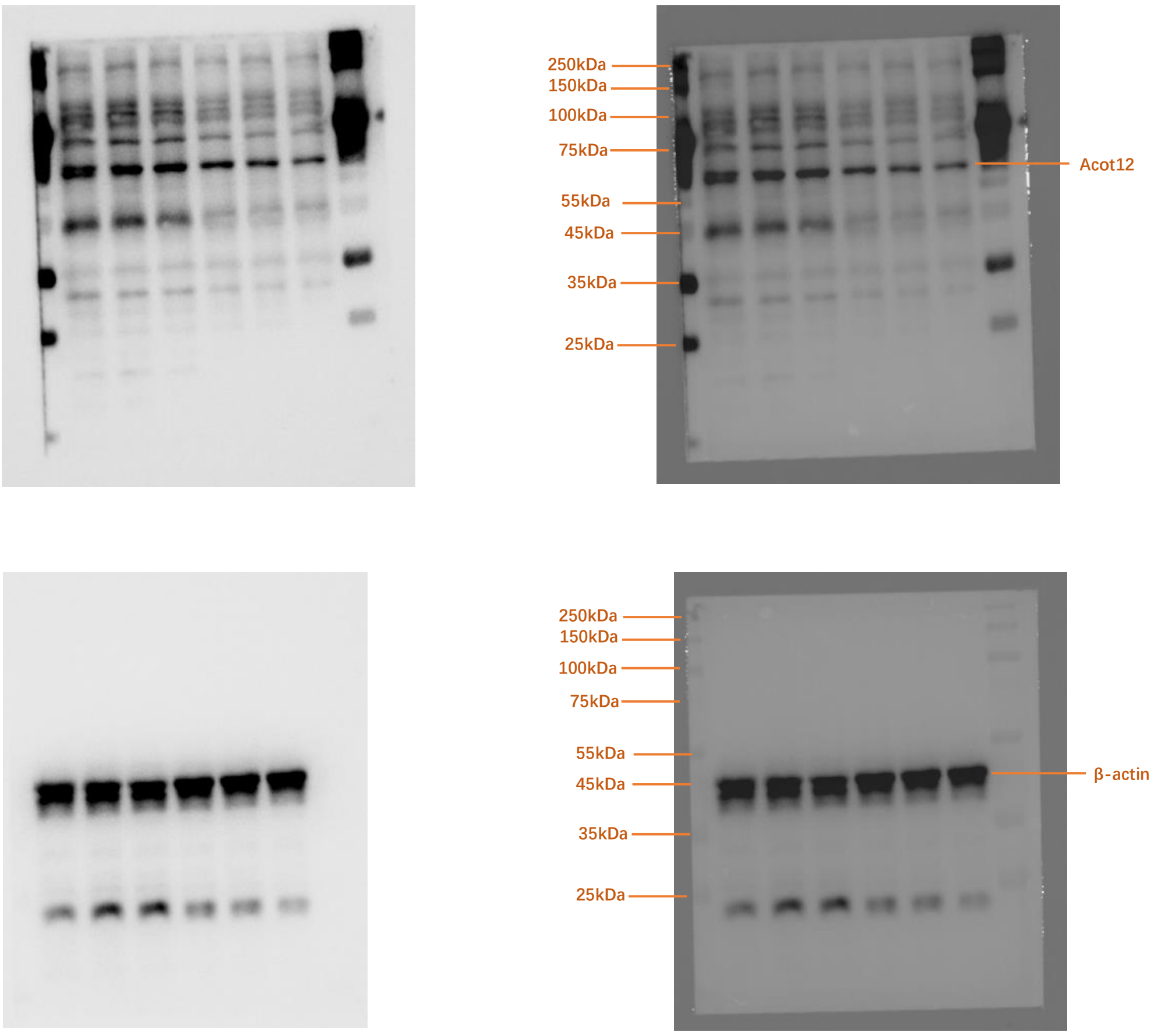

Extended Data Figure 17. The original gels for Western blot in Figure 7I. The left figure is the chemiluminescence image, and the right figure is the merged image.

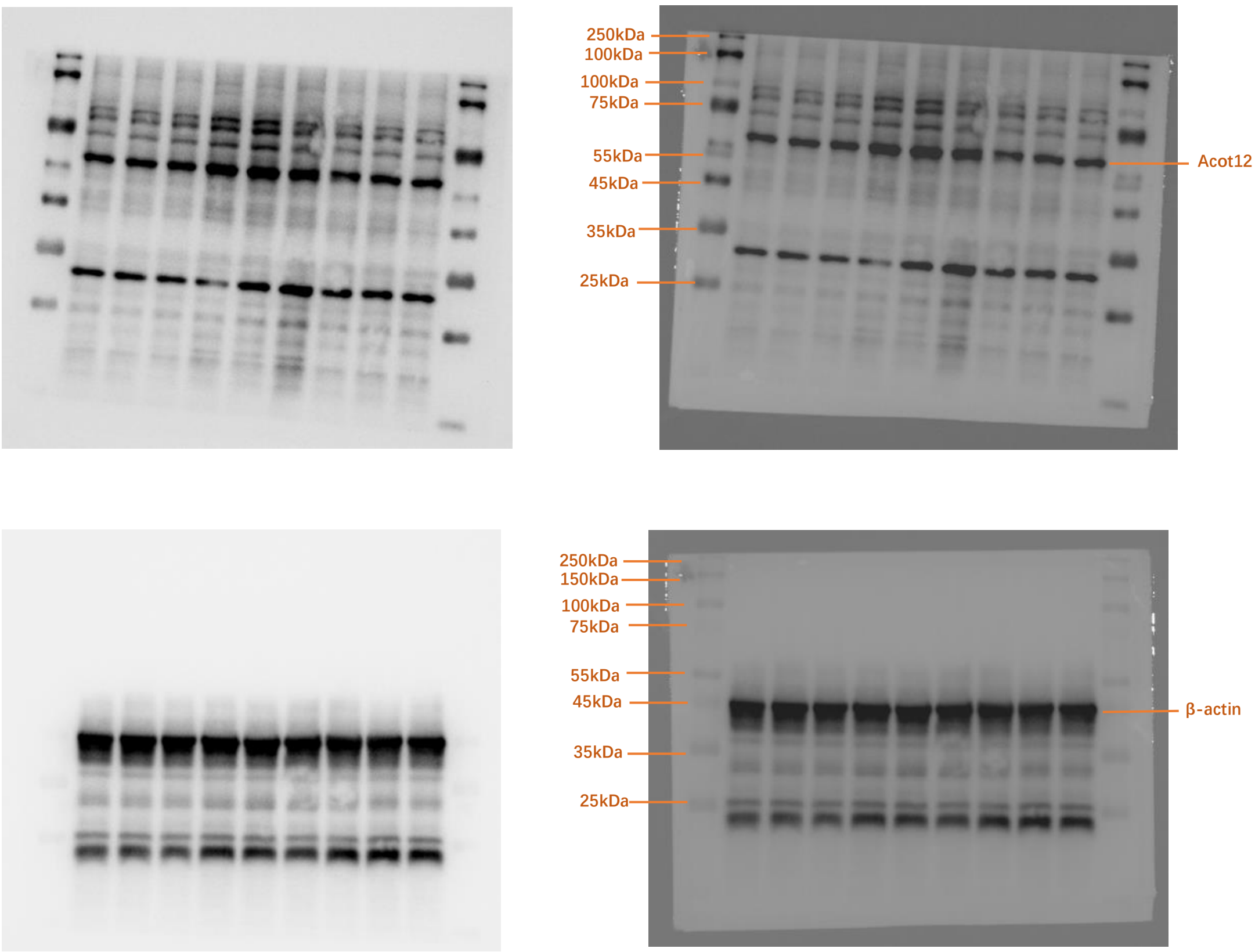

Extended Data Figure 18. The original gels for Western blot in Figure 7J. The left figure is the chemiluminescence image, and the right figure is the merged image.

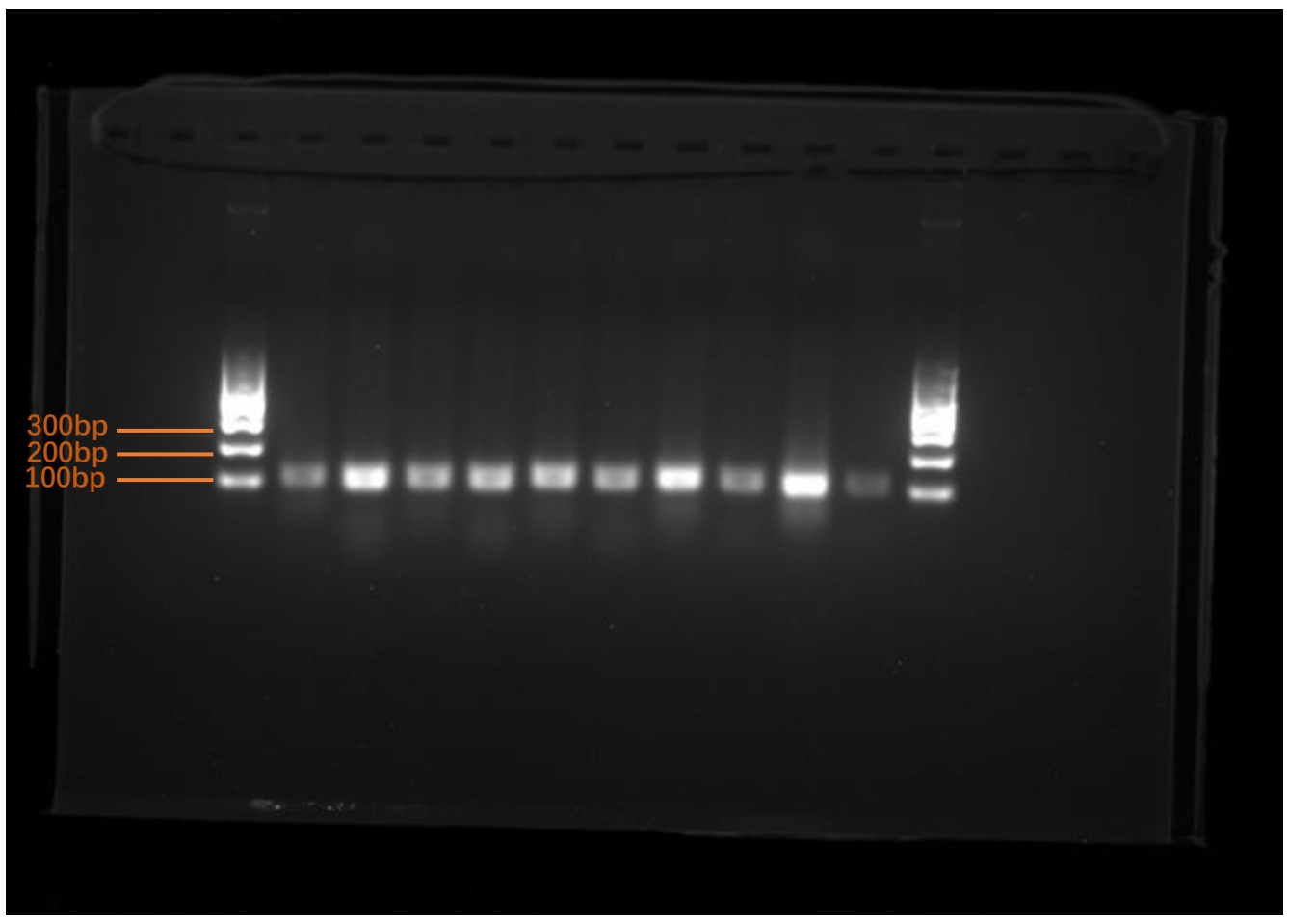

Extended Data Figure 19. The original gels of Figure 7K.

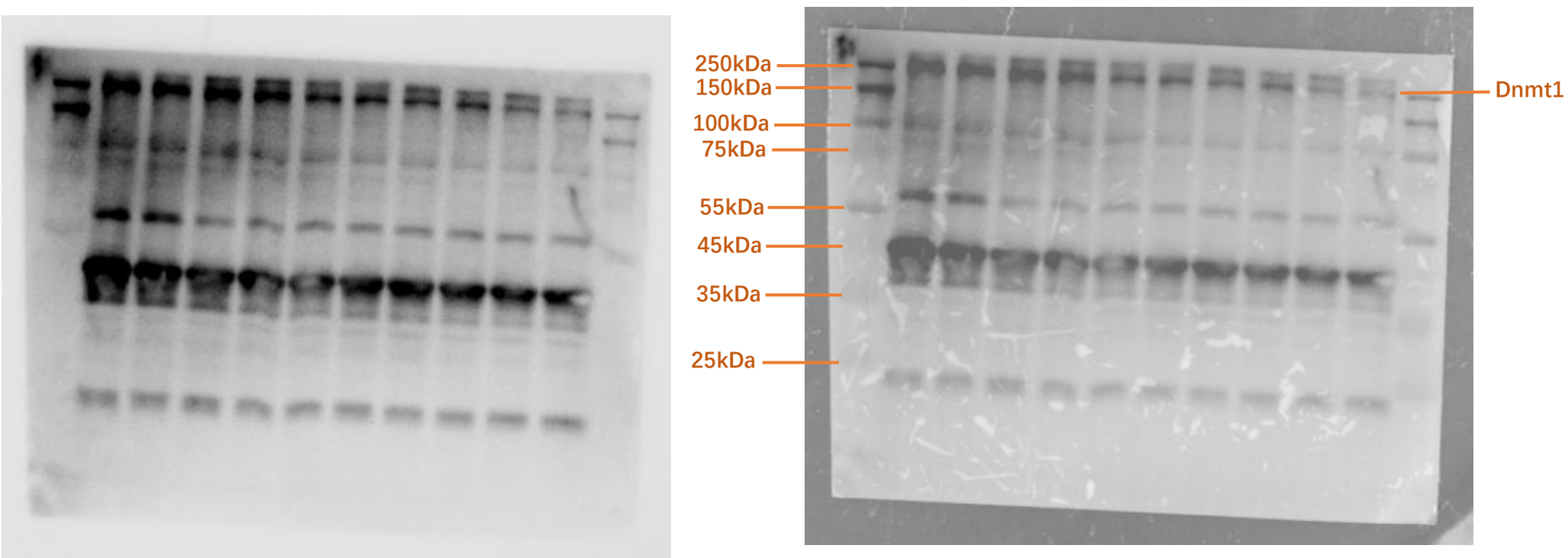
250kDa
150kDa
100kDa
75kDa
55kDa
45kDa
35kDa
25kDa
Dnmt1

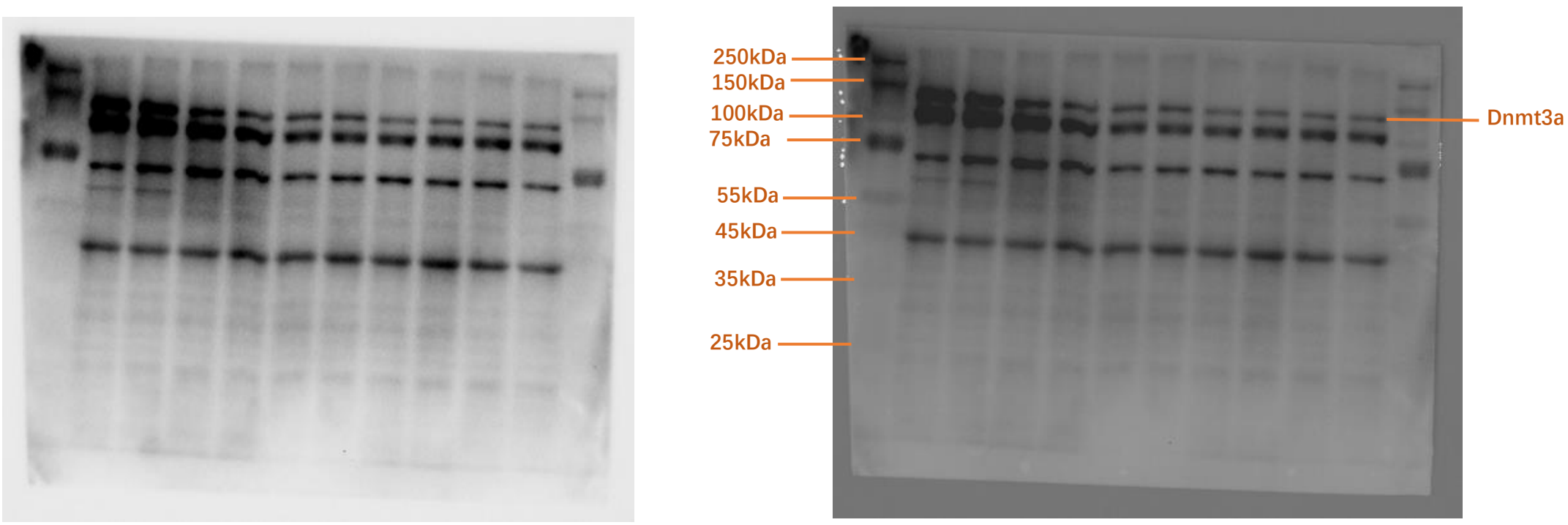
250kDa
150kDa
100kDa
75kDa
55kDa
45kDa
35kDa
25kDa
Dnmt3a

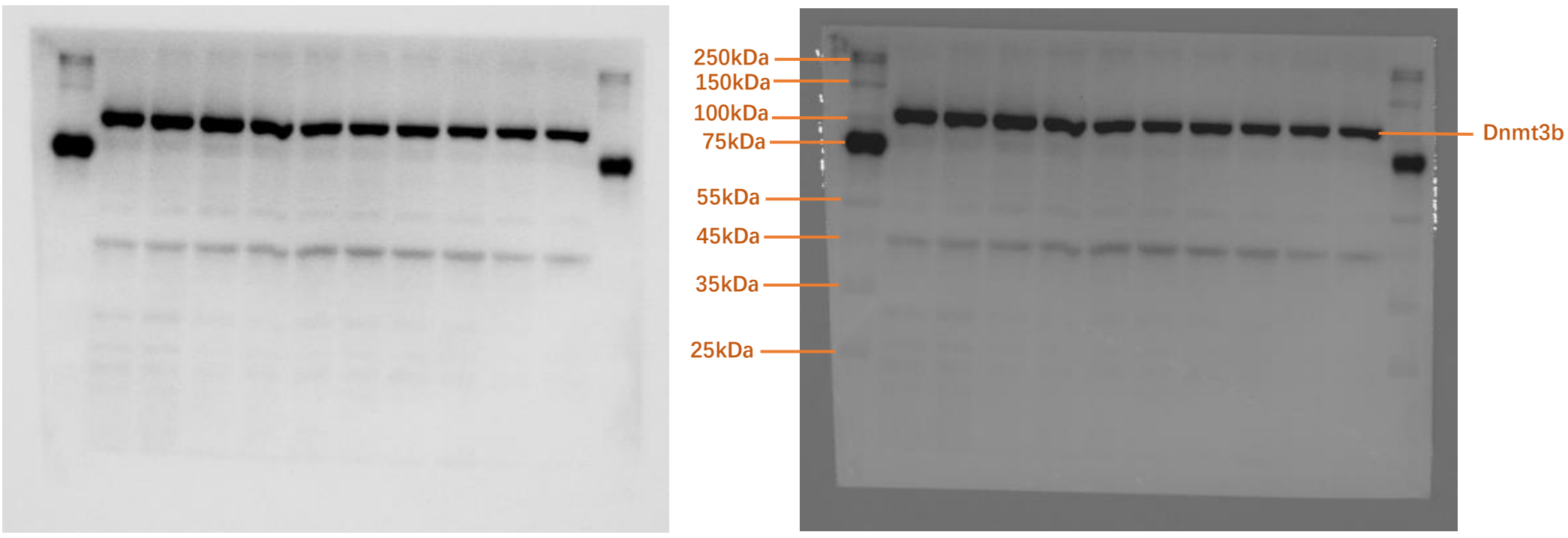
250kDa
150kDa
100kDa
75kDa
55kDa
45kDa
35kDa
25kDa
Dnmt3b

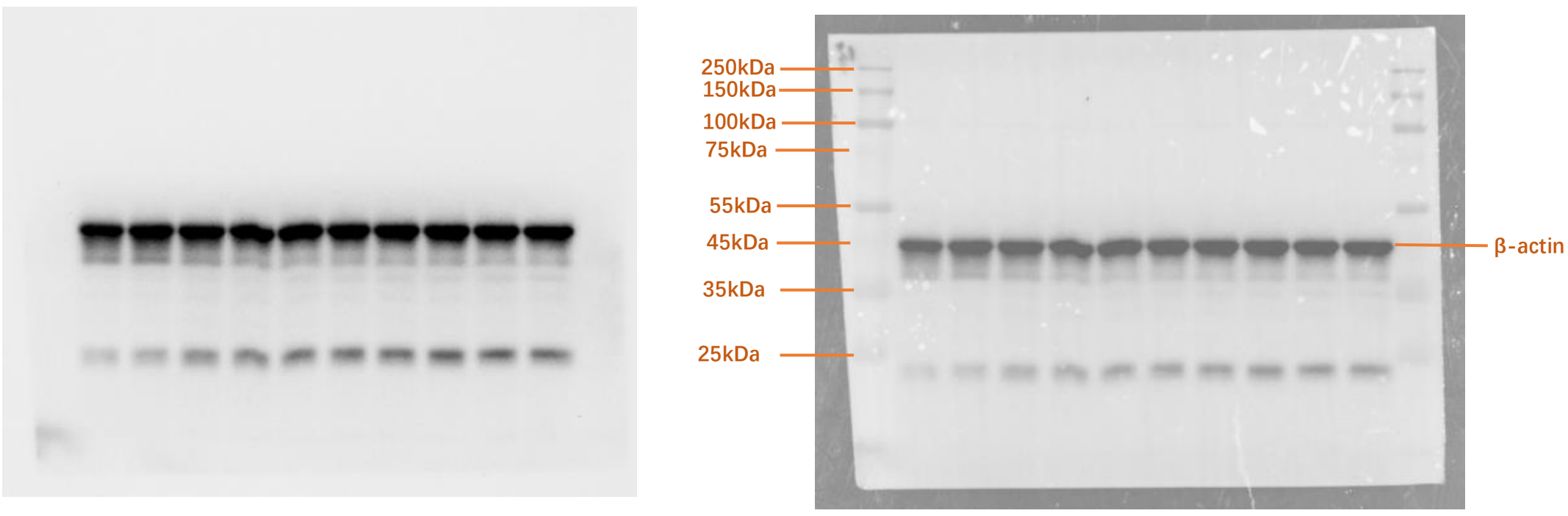
250kDa
150kDa
100kDa
75kDa
55kDa
45kDa
35kDa
25kDa
β-actin

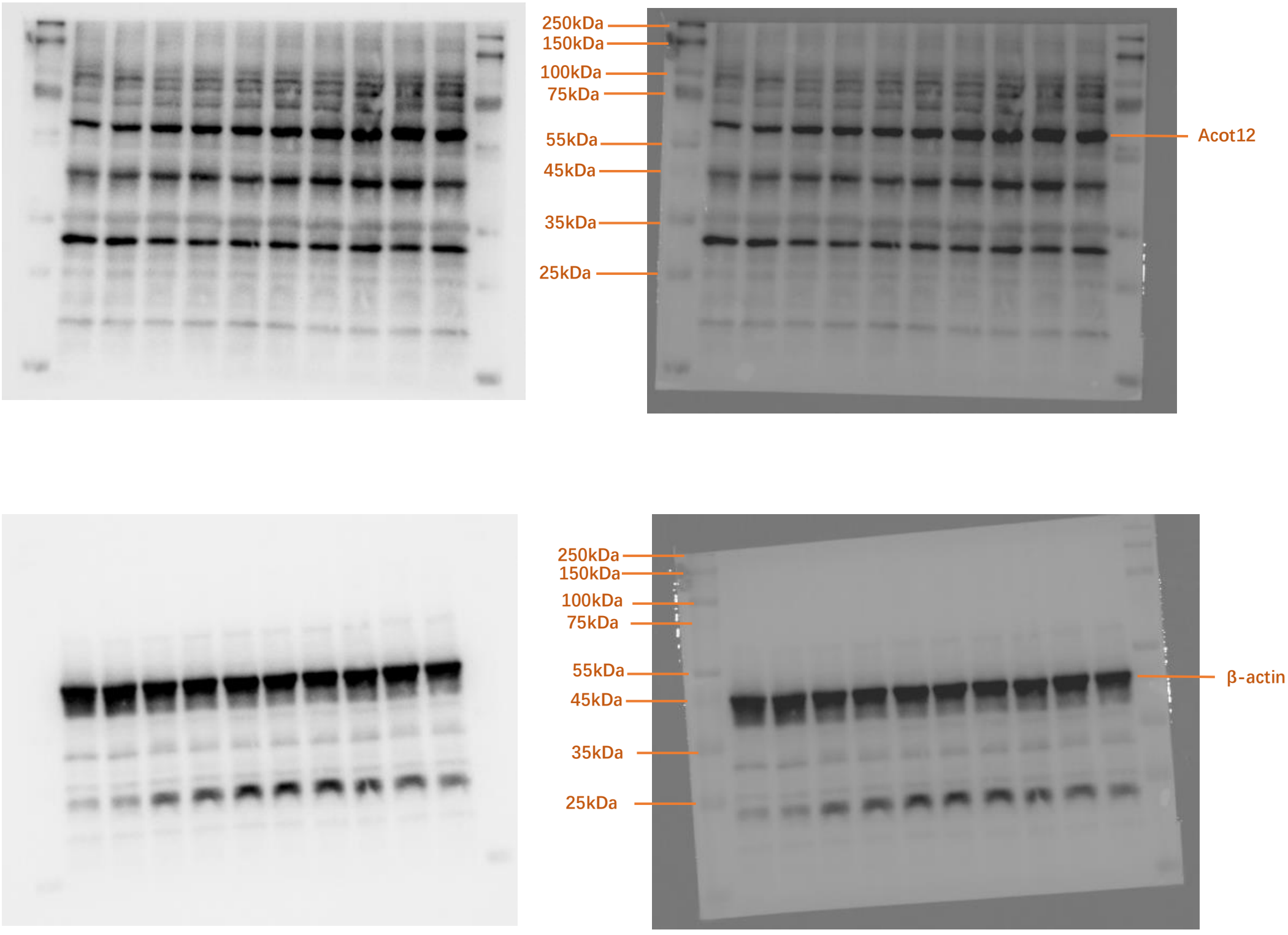

Extended Data Figure 20. The original gels for Western blot in Figure 7L. The left figure is the chemiluminescence image, and the right figure is the merged image.

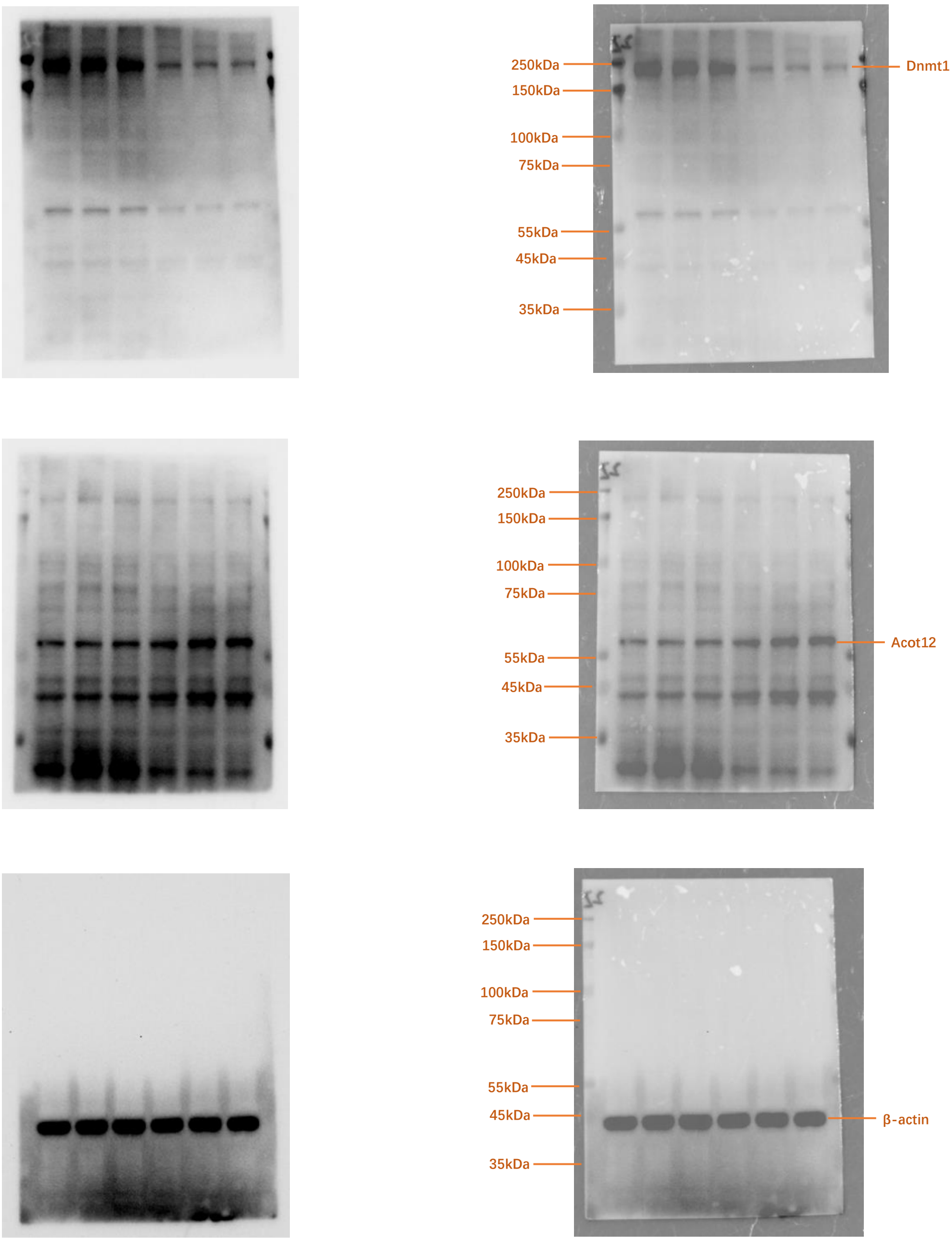

Extended Data Figure 21. The original gels for Western blot in Figure 7M. The left figure is the chemiluminescence image, and the right figure is the merged image.

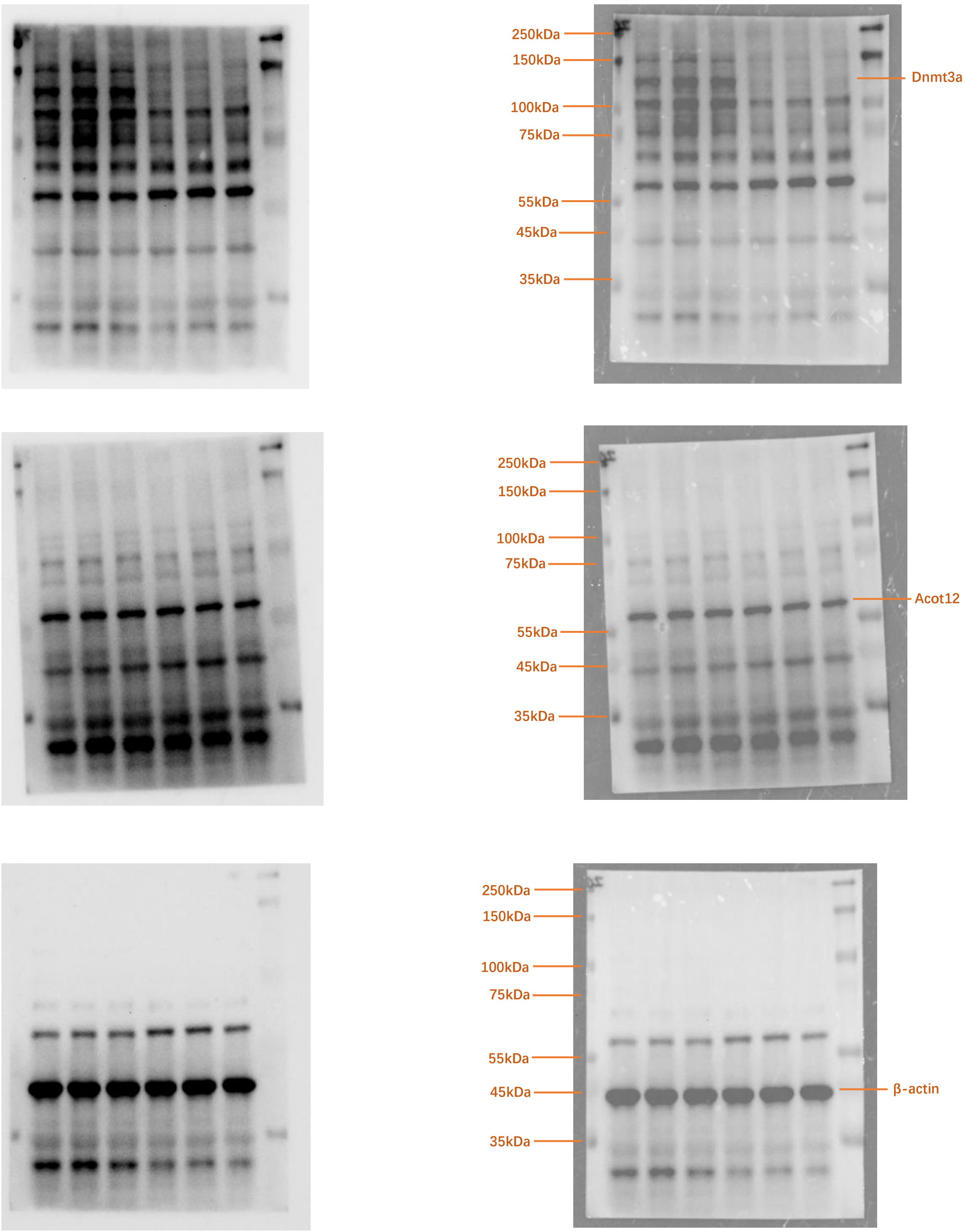

Extended Data Figure 22. The original gels for Western blot in Figure 7N. The left figure is the chemiluminescence image, and the right figure is the merged image.

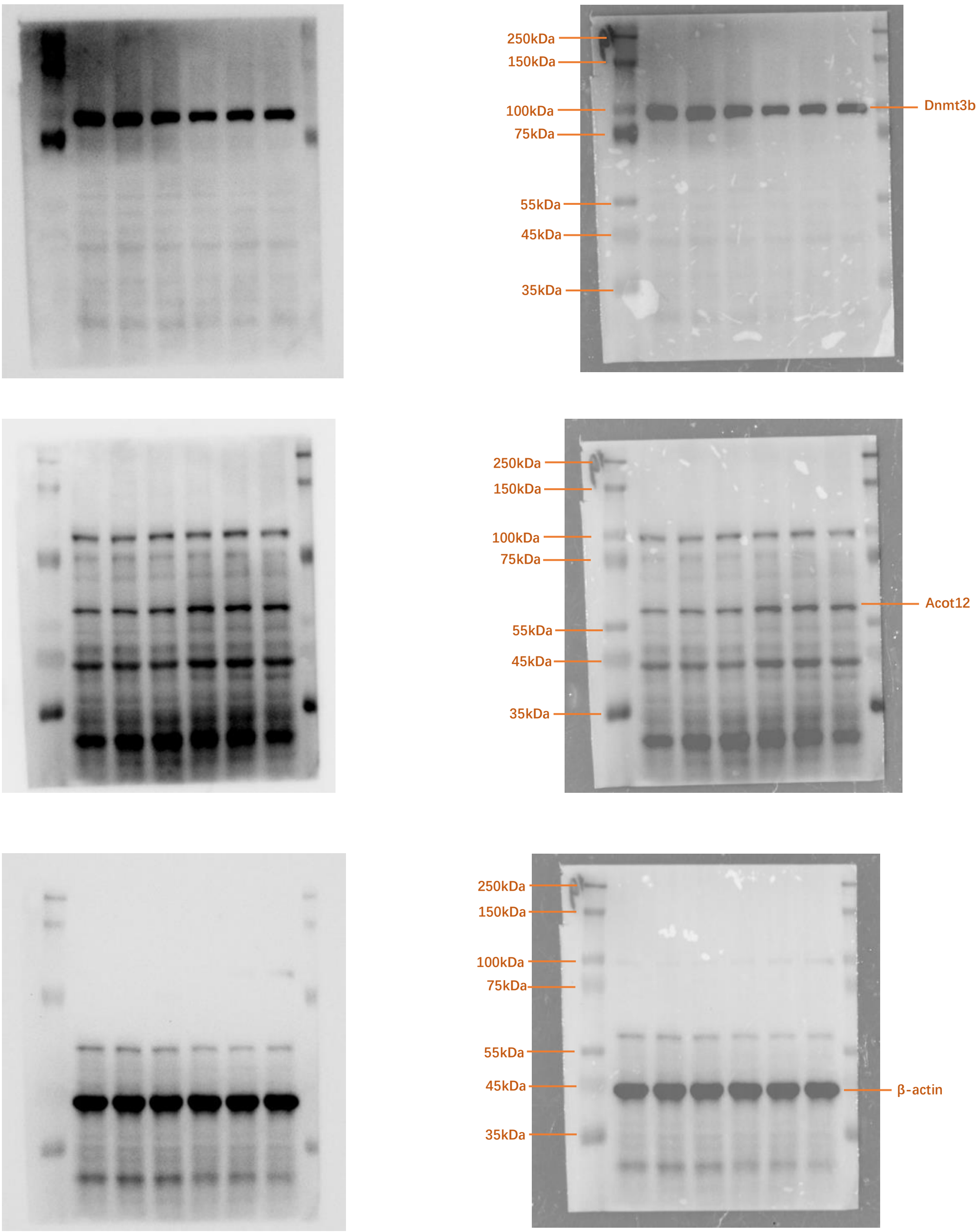

Extended Data Figure 23. The original gels for Western blot in Figure 7O. The left figure is the chemiluminescence image, and the right figure is the merged image.

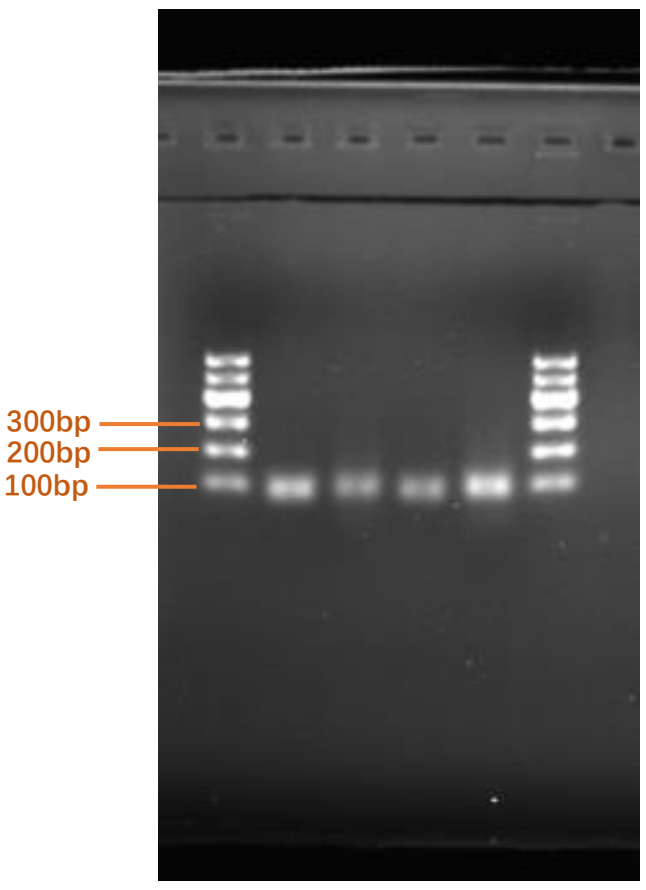

Extended Data Figure 24. The original gels of Figure S6G.

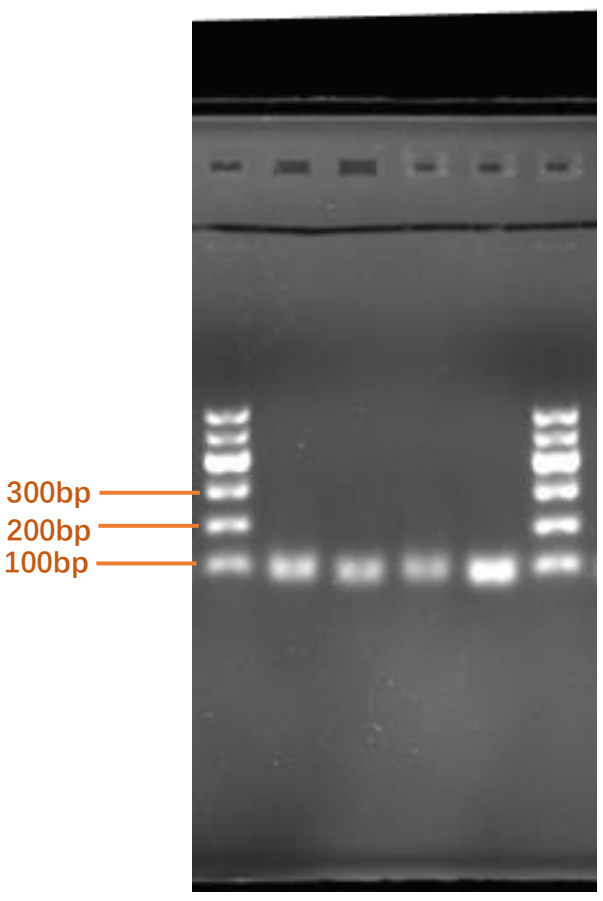

Extended Data Figure 25. The original gels of Figure S6H.

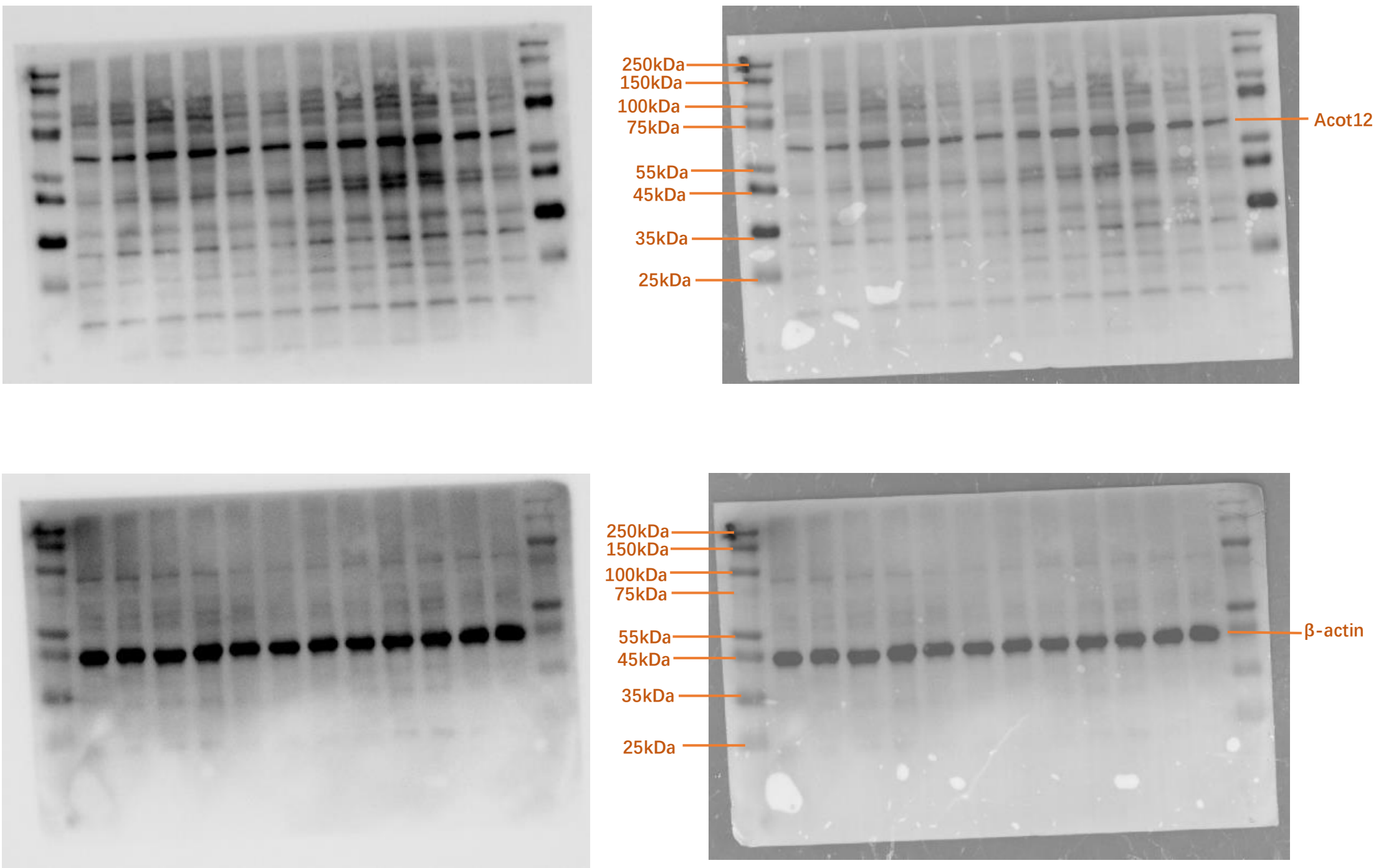

Extended Data Figure 26. The original gels for Western blot in Figure S6I. The left figure is the chemiluminescence image, and the right figure is the merged image.

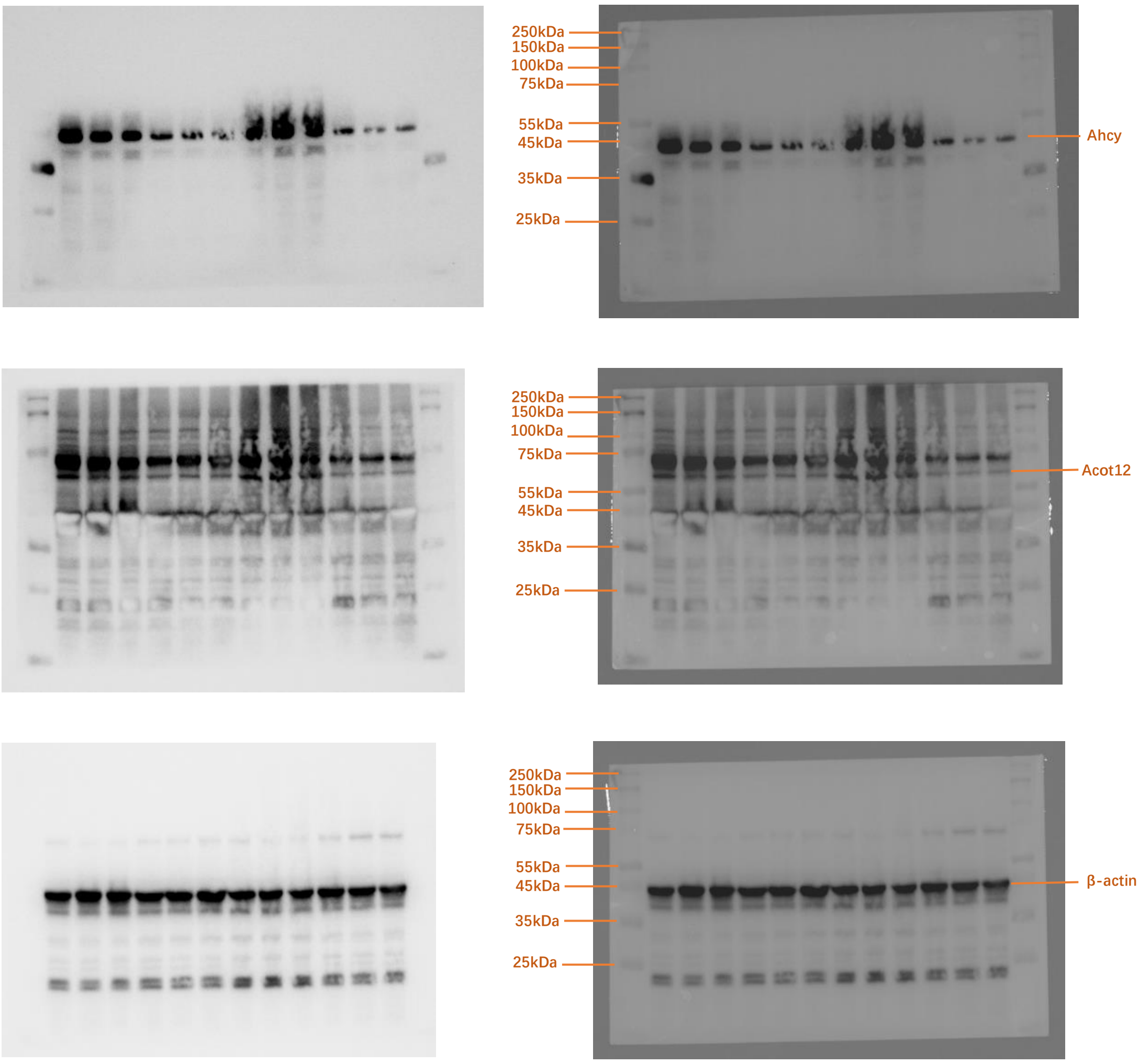

Extended Data Figure 27. The original gels for Western blot in Figure 8L. The left figure is the chemiluminescence image, and the right figure is the merged image.
