# Supplementary material for: Ahcy Acts as an Effector of Hnf4a‐Driven Super‐Enhancer Activation to Alleviate MASLD During Intermittent Fasting
Source: Adv Sci (Weinh). 2026 Jul 27;13(59):e76826. doi: 10.1002/advs.76826 (PMC13403733; doi:10.1002/advs.76826)
Supplement: Supplementary file 2 [file ADVS-13-e76826-s002.docx]

**Ahcy acts as an effector of Hnf4a-driven super-enhancer activation to alleviate MASLD during intermittent fasting**

Huafeng Chen^1^, Shilin Zhang^1^, Xiaojie Deng^4^, Wenqiang Xie^1^, Fen Xu^3^*****, Jie Shen^1^*****, Hua Liang^1, 2^*****

^1^Department of Endocrinology and Metabolism, The Eighth Affiliated Hospital, Southern Medical University (The First People's Hospital of Shunde, Foshan), Foshan City, Guangdong Province, China

^2^Medical Research Center, The Eighth Affiliated Hospital, Southern Medical University (The First People's Hospital of Shunde, Foshan), Foshan City, Guangdong Province, China

^3^Department of Endocrinology and Metabolism, The third Affiliated Hospital, Sun Yat-sen University, Guangzhou City, Guangdong Province, China

^4^Department of Endocrinology and Metabolism, Guangzhou First People's Hospital, Guangzhou City, Guangdong Province, China

*** Corresponding author:**
Hua Liang

Department of Endocrinology and Metabolism, The Eighth Affiliated Hospital, Southern Medical University (The First People's Hospital of Shunde, Foshan), Foshan City, Guangdong Province, China
E-mail addresses: [lhlianghua@smu.edu.cn](mailto:lhlianghua@smu.edu.cn)

Jie Shen

Department of Endocrinology and Metabolism, The Eighth Affiliated Hospital, Southern Medical University (The First People's Hospital of Shunde, Foshan), Foshan City, Guangdong Province, China
E-mail addresses: shenjiedr@163.com

Fen Xu

Department of Endocrinology and Metabolism, The Third Affiliated Hospital, Sun Yat-sen University, Guangzhou City, Guangdong Province, China

E-mail addresses: xufen3@mail.sysu.edu.cn

**This PDF file includes:** Supplementary Figure S1 to S9, Supplementary Table S1 to S7, and Supplementary Methods

**Supplementary Figure S1. IF improves metabolic disorder induced by high-fat diet in MASLD mice. IF may act by regulating fatty acid metabolism and inflammatory response pathways through SEs.**

A. Representative images of mice and livers in CD, iCD, HFD, and iHFD groups. B. Liver weight and liver-to-body weight ratio. C. Weight gain of mice. CD vs HFD, **p*＜0.05, ***p*＜0.01, ****p*＜0.001. HFD vs iHFD, #*p*＜0.05, ##*p*＜0.01, ###*p*＜0.001. CD vs iCD, & *p*＜0.05, && *p*＜0.01. D. Cumulative food intake. E. Fasting blood glucose. CD vs HFD, **p*＜0.05, ***p*＜0.01, ****p*＜0.001. HFD vs iHFD, #*p*＜0.05, ##*p*＜0.01, ###*p*＜0.001. F. ipGTT and ipITT curves and area under the curve (AUC) for each group of mice. G. Heatmap of ChIP-seq signal intensity around H3K27ac peak centers. H. GO-Cellular Component (CC) and GO-Biological Process (MF),analysis results for target genes regulated by SEs. I. KEGG enrichment analysis of DEGs in the transcriptome of HFD vs CD, and iHFD vs HFD groups. J. GO enrichment analysis of DEGs in the transcriptome of HFD vs CD, and iHFD vs HFD groups. **p* < 0.05, ***p* < 0.01, ****p* < 0.001.

**Supplementary Figure S2. Liver-specific Ahcy knockout exacerbates hepatic lesions in MASLD mice and reverses the beneficial effects induced by IF.**

A. Representative images of mice and livers in Ahcy^fl/fl^+HFD, Ahcy^LKO^+HFD, Ahcyfl/fl+iHFD and AhcyLKO+iHFD groups. B. Liver weightt and liver-to-body weight ratio. C. Weight gain of mice. Ahcy^fl/fl^+HFD vs Ahcy^fl/fl^+iHFD, **p*＜0.05, ***p*＜0.01, ****p*＜0.001. Ahcy^LKO^+HFD vs Ahcy^LKO^+iHFD, #*p*＜0.05, ##*p*＜0.01, ###*p*＜0.001. D. Cumulative food intake. E. Fasting blood glucose. Ahcy^fl/fl^+ HFD vs Ahcy^fl/fl^+ iHFD, **p*＜0.05, ***p*＜0.01, ****p*＜0.001. Ahcy^LKO^+HFD vs Ahcy^LKO^+iHFD, #*p*＜0.05, ##*p*＜0.01, ###*p*＜0.001. F. ipGTT and ipITT curves and area under the curve (AUC) for each group of mice. G. Serum TG and TC levels. H. KEGG enrichment analysis of DEGs in the transcriptome of Ahcy^LKO^+HFD vs Ahcy^fl/fl^+HFD, and Ahcy^LKO^+iHFD vs Ahcy^fl/fl^+iHFD groups. I. GO enrichment analysis of DEGs in the transcriptome of Ahcy^LKO^+HFD vs Ahcy^fl/fl^+HFD, and Ahcy^LKO^+iHFD vs Ahcy^fl/fl^+iHFD groups. J. The protein expression and quantitative analysis of Ahcy after intervention with siAhcy in AML12 cells. K. The mRNA expression of Ahcy after intervention with siAhcy in AML12 cells. L. TC and TG content in AML12 cells after siAhcy transfection. M. Oil Red O and BODIPY staining of AML12 cells after transfection with siAhcy.**p* < 0.05, ***p* < 0.01, ****p* < 0.001.

**Supplementary Figure S3. Ahcy is a gene driven by the SE. The primary active component E3 of the Ahcy-SE was deleted in AML12 cells using the CRISPR-Cas9 system.**

A. ChIP-seq revealed prominent H3K27ac peaks in the Ahcy-SE region of livers from CD- and HFD-fed mice (data from GSE226171). B. The topologically associated domain (TAD) region encompassing Ahcy in mouse liver was predicted based on Hi-C data (<https://3dgenome.fsm.northwestern.edu/vis?datasets=487%2C486>). C. gRNA binding sites and PCR identification results of CRISPR/CAS9 gene editing technology in AML12 cells. D. Sequencing results of E3 knockout fragments in AML12 cells. **p*＜0.05, ***p*＜0.01, ****p*＜0.001.

**Supplementary Figure S4. Brd4 inhibition downregulates the expression of Ahcy. In vivo inhibition of Brd4 accelerates MASLD progression and weakens the beneficial effect of IF on the disease. Transcriptome analysis reveals that inflammatory response and fatty acid metabolic pathways were significantly enriched after inhibiting Brd4.**

A. The protein expression of Brd4 and Ahcy after 72h of JQ1-PA treatment. B. The mRNA expression of Brd4 and Ahcy after 72h of JQ1-PA (5uM) treatment. C. The protein expression of Brd4 and Ahcy after 48h of siBrd4 treatment. D. The mRNA expression of Brd4 and Ahcy after 48h of siBrd4 (50nM) treatment. E. Representative images of mice and livers in CD+AAV-Ctrl, CD+AAV-shBrd4, HFD+AAV-Ctrl, HFD+AAV-shBrd4, iHFD+AAV-Ctrl, and iHFD+AAV-shBrd4 groups. F. Liver weightt and liver-to-body weight ratio. G. Weight gain of mice. CD+AAV-Ctrl vs HFD+AAV-Ctrl, **p*＜0.05, ****p*＜0.001. CD+AAV- shBrd4 vs HFD+AAV- shBrd4, #*p*＜0.01, ###*p*＜0.001. HFD+AAV-Ctrl vs iHFD+AAV-Ctrl, &*p*＜0.05, &&&*p*＜0.001. HFD+AAV- shBrd4 vs. iHFD+AAV- shBrd4, $*p*＜0.05, $$$*p*＜0.001. H. Cumulative food intake. I. Fasting blood glucose. HFD+AAV-Ctrl vs HFD+AAV-shBrd4, ***p*＜0.01. iHFD+AAV-Ctrl vs iHFD+AAV-shBrd4, ##*p*＜0.01. J. ipGTT and ipITT curves and area under the curve (AUC) for each group of mice. K. Serum TG and TC levels. L. KEGG enrichment analysis of DEGs in the transcriptome of CD+AAV-shBrd4 vs CD+AAV-Ctrl, HFD+AAV-shBrd4 vs HFD+AAV-Ctrl and iHFD+AAV-shBrd4 vs iHFD+AAV-Ctrl groups. M. GO enrichment analysis of DEGs in the transcriptome of CD+AAV-shBrd4 vs CD+AAV-Ctrl, HFD+AAV-shBrd4 vs HFD+AAV-Ctrl and iHFD+AAV-shBrd4 vs iHFD+AAV-Ctrl groups. **p*＜0.05, ***p*＜0.01, ****p*＜0.001.

**Supplementary Figure S5. Hnf4a activates Ahcy transcription by binding to the Ahcy-E3 and promoter. Overexpression of Hnf4a in vivo promotes Ahcy expression, alleviates hepatic lesions and metabolic disorders in MASLD mice. Knockdown of Ahcy reverses the mitigating effects of Hnf4a.**

A. ChIP-seq revealed significant Hnf4a enrichment in the Ahcy-E3 and promoter region of mouse liver (data from GSE118007). B. Schematic diagram of the spatial binding pattern of Hnf4a protein to the Ahcy promoter and E3. C. Representative images of mice and livers in HFD+AAV-Ctrl+AAV-shCtrl, HFD+AAV-Hnf4a+AAV-shCtrl, HFD+AAV-Hnf4a+AAV-shAhcy, iHFD+AAV-Ctrl+AAV-shCtrl, iHFD+AAV-Hnf4a+AAV-shCtrl, and iHFD+AAV-Hnf4a+AAV-shAhcy groups. D. Liver weightt and liver-to-body weight ratio. E. Weight gain of mice. HFD+AAV-Hnf4a+AAV-shAhcy vs HFD+AAV-Hnf4a+AAV-shCtrl, #*p*＜0.05. F. Cumulative food intake. G. Fasting blood glucose. HFD+AAV-Hnf4a+AAV-shCtrl vs HFD+AAV-Ctrl+AAV-shCtrl, ****p*＜0.001. HFD+AAV-Hnf4a+AAV-shAhcy vs HFD+AAV-Hnf4a+AAV-shCtrl, ###*p*＜0.001. iHFD+AAV-Hnf4a+AAV-shAhcy vs iHFD+AAV-Hnf4a+AAV-shCtrl, &&&*p*＜0.001. H. ipGTT and ipITT curves and area under the curve (AUC) for each group of mice. I. Serum TG and TC levels. **p*＜0.05, ***p*＜0.01, ****p*＜0.001.

**Supplementary Figure S6. DMR-annotated genes are enriched in fatty acid metabolism and transcriptional regulation pathways after hepatic Ahcy knockout. After Ahcy inhibition, the methylation level of the Acot12 promoter increases, while Acot12 expression decreases.**

A. DMRs distribution ratio map in anchored regions. B. Violin plot of average methylation levels in DMRs. C. Circos diagram showing the significance of DMRs distribution across the genome. From outside to inside in the figure: A scatter plot of hyper-DMR distribution, where points closer to the outer edge indicate higher significance; A sequence GC content heatmap, where darker colors denote higher GC content; A sequence gene density heatmap, where darker colors indicate higher gene content; A scatter plot of hypo-DMR distribution, where points closer to the inner edge indicate higher significance. D. KEGG enrichment analysis of genes associated with DMRs anchoring. E. GO enrichment analysis of genes associated with DMRs anchoring. F. Distribution of CpG islands of DMRs of Acot12 promoter. G. Methylation level of DMR of Acot12 promoter in the stable Ahcy-knockdown AML12 cells. H. Effect of Ahcy knockdown by siAhcy on the DMR methylation level of Acot12 in AML12 cells. I. After overexpressing Hnf4a and knocking down Ahcy in the liver, the protein expression and quantitative analysis of Acot12 in each group of mice. J. After overexpressing Hnf4a and knocking down Ahcy in the liver, the mRNA expression of Acot12 in each group of mice. **p*＜0.05, ***p*＜0.01, ****p*＜0.001.

**Supplementary Figure S7.** **Knocking down Ahcy reduces Acot12 expression.**

A. Quantitative analysis of protein expression of Figure 7F. B. Quantitative analysis of protein expression of Figure 7H. C. Quantitative analysis of protein expression of Figure 7I. D. Quantitative analysis of protein expression of Figure 7J. E. Quantitative analysis of protein expression of Figure 7L. F. Quantitative analysis of protein expression of Figure 7M. G. Quantitative analysis of protein expression of Figure 7N. H. Quantitative analysis of protein expression of Figure 7O. **p*＜0.05, ***p*＜0.01, ****p*＜0.001.

**Supplementary Figure S8.** **Betaine supplementation alleviates the aggravated hepatic lipid accumulation caused by Ahcy deficiency in MASLD.**

A. Mouse Intervention Protocol. 8-week-old male C57BL/6J mice (n=8) were fed a high-fat diet to induce MASLD. The HFD+AAV-shAhcy+NC and HFD+AAV-shAhcy+Betaine group had ad libitum access to food and water. The iHFD+AAV-shAhcy+NC and iHFD+AAV-shAhcy+Betaine group underwent IF. After 12 weeks, all groups received tail vein injections of AAV8- shAhcy. The HFD+AAV-shAhcy+NC and iHFD+AAV-shAhcy+NC group were provided with normal drinking water. The HFD+AAV-shAhcy+Betaine and iHFD+AAV-shAhcy+Betaine group received drinking water supplemented with 1.5% (w/v) betaine. All groups continued feeding on their respective diets for an additional 8 weeks. B. Representative images of mice and livers. C. Liver weightt and liver-to-body weight ratio. D. Weight gain of mice. HFD+AAV-shAhcy+NC vs HFD+AAV-shAhcy+Betaine, **p* < 0.05, ***p* < 0.01. iHFD+AAV-shAhcy+NC vs iHFD+AAV-shAhcy+Betaine, #*p*＜0.05, ##*p*＜0.01. E. Fasting blood glucose. HFD+AAV-shAhcy+NC vs. HFD+AAV-shAhcy+Betaine, ***p*＜0.01, ****p*＜0.001. F. ipGTT and ipITT curves and area under the curve (AUC) for each group of mice. G. Liver tissue TG and TC levels in each group. H. Serum TG and TC levels. I. Serum ALT and AST levels. J. The mRNA expression levels of hepatic fat formation indicators Srebf1, Fasn, Scd1 and inflammatory indicators Il6, Tnfa, Il1b. K. Upper figure: Oil Red O staining of liver tissues and quantitative analysis of lipid droplets (bar graph). Lower figure: HE staining of liver tissues (20×). **p*＜0.05, ***p*＜0.01, ****p*＜0.001.

**Supplementary Figure S9. The molecular mechanism diagram of IF improving MASLD through SE-driven Ahcy.** IF increases the expression of Hnf4a, which drives the high expression of Ahcy by binding to Ahcy promoter and E3 region. Ahcy alters the levels of SAM and SAH, and regulates the function of methyltransferases, reducing the DNA methylation of the Acot12 promoter, thereby enhancing its expression and improving MASLD.

**Supplementary Table S1. Sequences of siRNA.**

| Target gene | siRNA name | Sequence (5' -> 3') |
| --- | --- | --- |
| Brd4 (mouse) | Sense | CCUCUCCGCUUAUGAUACATT |
|  | Antisense | UGUAUCAUAAGCGGAGAGGTT |
| Ahcy (mouse) | Sense | GCAAGUUUGACAACCUCUATT |
|  | Antisense | UAGAGGUUGUCAAACUUGCTT |
| Brd4 (human) | Sense | GGAAAGAGGAAGUGGAAGATT |
|  | Antisense | UCUUCCACUUCCUCUUUCCTT |
| Dnmt1 (mouse) | Sense | GGAAAGAGAUGGCUUAACATT |
|  | Antisense | UGUUAAGCCAUCUCUUUCCTT |
| Dnmt3a (mouse) | Sense | GCAGAACAAGCAGAUGAUUTT |
|  | Antisense | AAUCAUCUGCUUGUUCUGCTT |
| Dnmt3b (mouse) | Sense | GCAAUGAUCUCUCUAACGUTT |
|  | Antisense | ACGUUAGAGAGAUCAUUGCTT |

**Supplementary Table S2. Primers for qRT-PCR or** **ChIP-qPCR.**

| Genes | Primers | Sequences (5' -> 3') | Application |
| --- | --- | --- | --- |
| Ahcy (mouse) | Forward | ATGTCAACGATTCTGTCACCAAGAG | qRT-PCR |
|  | Reverse | TCACCATAGCCTGCCACCAC |  |
| Brd4 (mouse) | Forward | CAAGATGCCTGATGAGCCTGAAG | qRT-PCR |
|  | Reverse | GAACTGTCGCTGTCGGAAGAAC |  |
| β-actin (mouse) | Forward | ACTGCCGCATCCTCTTCCTC | qRT-PCR |
|  | Reverse | AACCGCTCGTTGCCAATAGTG |  |
| Hnf4a (mouse) | Forward | CGGAGGGTCTGCCAGTGATG | qRT-PCR |
|  | Reverse | TGTCCATTGCTGAGGTGAGAGG |  |
| Brd4 (human) | Forward | TCGTGGTGGTGAAGGAGGAGAAG | qRT-PCR |
|  | Reverse | GGCGGTGCGTTCTGCTCTG |  |
| β-actin (human) | Forward | ATCGTGCGTGACATTAAGGAGAAG | qRT-PCR |
|  | Reverse | AGGAAGGAAGGCTGGAAGAGTG |  |
| Il6 (mouse) | Forward | CTTCTTGGGACTGATGCTGGTGAC | qRT-PCR |
|  | Reverse | AGGTCTGTTGGGAGTGGTATCCTC |  |
| Tnfa (mouse) | Forward | CTGAACTTCGGGGTGATCGG | qRT-PCR |
|  | Reverse | GGCTTGTCACTCGAATTTTGAGA |  |
| Il1b (mouse) | Forward | GAAATGCCACCTTTTGACAGTG | qRT-PCR |
|  | Reverse | TGGATGCTCTCATCAGGACAG |  |
| Scd1 (mouse) | Forward | AGCCTGTTCGTTAGCACCTTCTTG | qRT-PCR |
|  | Reverse | GCACCCAGGGAAACCAGGATATTC |  |
| Fasn (mouse) | Forward | TGCCCGAGTCAGAGAACCTACAG | qRT-PCR |
|  | Reverse | TCCATAGAGCCCAGCCTTCCATC |  |
| Srebf1 (mouse) | Forward | TGACCCGGCTATTCCGTGA | qRT-PCR |
|  | Reverse | CTGGGCTGAGCAATACAGTTC |  |
| Acot12 (mouse) | Forward | GTTTGAGGACACAGCGAGAAT | qRT-PCR |
|  | Reverse | GAAGCAGGACAGGTTTTAAGTGA |  |
| Col1a1 (mouse) | Forward | CGCCATCAAGGTCTACTGC | qRT-PCR |
|  | Reverse | ACGGGAATCCATCGGTCA |  |
| Tgfb1 (mouse) | Forward | ACTGGAGTTGTACGGCAGTG | qRT-PCR |
|  | Reverse | GGGGCTGATCCCGTTGATT |  |
| a-SMA (mouse) | Forward | CCATGTATGTGGCTATTCAGG | qRT-PCR |
|  | Reverse | AAGCGTTCGTTTCCAATGGTG |  |
| Ahcy-Promoter (mouse) | Forward | GGCACAGTCCTCAGTTTTGG | ChIP-qPCR |
|  | Reverse | CTGCTCCTCCTCCTATGGTG |  |
| Ahcy-E3 (mouse) | Forward | AGGAGATGGGACTGGACTCT | ChIP-qPCR |
|  | Reverse | AGAGGAAGGCTGAGGTAGGA |  |
| Acot12-Promoter (mouse) | Forward | TCTCCGGAGAGCATGCAATC | ChIP-qPCR |
|  | Reverse | TCTCTCGGAGCAGAACAGGA |  |

**Supplementary Table S3. Antibodies used for Western bolt and ChIP-qPCR.**

| Antibodies | Company | Cat No. | Application |
| --- | --- | --- | --- |
| Ahcy | abcam | ab317034 | WB |
| Hnf4a | abcam | ab181604 | WB, ChIP-qPCR |
| Brd4 | proteintech | 28486-1-AP | WB |
| Acot12 | Servicebio | GB111565 | WB |
| Dnmt1 | cellsignal | 5032 | WB, ChIP-qPCR |
| Dnmt3a | zenbio | R381093 | WB |
| Dnmt3b | proteintech | 26971-1-AP | WB, ChIP-qPCR |
| Brd4 | abcam | ab243862 | ChIP-qPCR |
| H3K27ac | proteintech | 82902-1-RR | ChIP-qPCR |

**Supplementary Table S4. Primers for MSP.**

| Genes | Primers | Sequences |
| --- | --- | --- |
| Acot12 (mouse) | Left M primer | TTATTTTTTAAGTATTTGGTAGCGG |
|  | Right M primer | CTAACTAAAAAAACCCCAAACG |
|  | Left U primer | TTTTTATTTTTTAAGTATTTGGTAGTGG |
|  | Right U primer | CTAACTAAAAAAACCCCAAACACC |

**Supplementary Table S5. Binding sites of TFs to Ahcy-E3 predicted by JASPAR (top10).**

| Matrix ID | Name | Score | Relative score | Sequence ID | Start | End | Strand | Predicted sequence |
| --- | --- | --- | --- | --- | --- | --- | --- | --- |
| MA1494.1 | MA1494.1.HNF4A | 16.54334 | 0.903254 | NC_000068.7:155080506-155082876 | 690 | 704 | - | AGGTACAAAGGGCAA |
| MA1494.2 | MA1494.2.HNF4A | 16.45029 | 0.905769 | NC_000068.7:155080506-155082876 | 691 | 704 | - | AGGTACAAAGGGCA |
| MA0114.2 | MA0114.2.HNF4A | 14.16443 | 0.923413 | NC_000068.7:155080506-155082876 | 690 | 704 | + | TTGCCCTTTGTACCT |
| MA0114.5 | MA0114.5.HNF4A | 12.92228 | 0.92685 | NC_000068.7:155080506-155082876 | 916 | 924 | - | CAGAGTCCA |
| MA0114.4 | MA0114.4.HNF4A | 12.86579 | 0.903488 | NC_000068.7:155080506-155082876 | 914 | 926 | - | GGCAGAGTCCAGT |
| MA0512.1 | MA0512.1.Rxra | 12.64104 | 0.946411 | NC_000068.7:155080506-155082876 | 689 | 699 | - | CAAAGGGCAAG |
| MA0148.3 | MA0148.3.FOXA1 | 12.20439 | 0.927636 | NC_000068.7:155080506-155082876 | 1295 | 1309 | - | AGTTTATTTACCCTG |
| MA0098.2 | MA0098.2.Ets1 | 12.12634 | 0.889365 | NC_000068.7:155080506-155082876 | 1797 | 1811 | + | ACCTCTTCCTCTTCA |
| MA0512.1 | MA0512.1.Rxra | 11.87108 | 0.935321 | NC_000068.7:155080506-155082876 | 914 | 924 | - | CAGAGTCCAGT |
| MA0148.2 | MA0148.2.FOXA1 | 11.30201 | 0.913075 | NC_000068.7:155080506-155082876 | 1295 | 1305 | - | TATTTACCCTG |

**Supplementary Table S6. Binding sites of TFs to Ahcy-promoter predicted by JASPAR (top10).**

| Matrix ID | Name | Score | Relative score | Sequence ID | Start | End | Strand | Predicted sequence |
| --- | --- | --- | --- | --- | --- | --- | --- | --- |
| MA1494.1 | MA1494.1.HNF4A | 18.54341 | 0.927551 | NC_000068.7:c155076497-155074398 | 1871 | 1885 | + | GGGTCCAAAGGCCAC |
| MA1494.2 | MA1494.2.HNF4A | 18.14299 | 0.926606 | NC_000068.7:c155076497-155074398 | 1871 | 1884 | + | GGGTCCAAAGGCCA |
| MA0114.2 | MA0114.2.HNF4A | 17.72782 | 0.970845 | NC_000068.7:c155076497-155074398 | 1871 | 1885 | - | GTGGCCTTTGGACCC |
| MA0512.1 | MA0512.1.Rxra | 15.26621 | 0.984224 | NC_000068.7:c155076497-155074398 | 1794 | 1804 | - | CAGAGGTCAAA |
| MA0512.1 | MA0512.1.Rxra | 13.00963 | 0.95172 | NC_000068.7:c155076497-155074398 | 938 | 948 | + | CACAGGTCAGG |
| MA0114.4 | MA0114.4.HNF4A | 12.99582 | 0.906078 | NC_000068.7:c155076497-155074398 | 1874 | 1886 | + | TCCAAAGGCCACA |
| MA0148.2 | MA0148.2.FOXA1 | 12.73026 | 0.937575 | NC_000068.7:c155076497-155074398 | 1475 | 1485 | - | TGTTTGCCTTT |
| MA0148.1 | MA0148.1.FOXA1 | 12.71143 | 0.937002 | NC_000068.7:c155076497-155074398 | 1475 | 1485 | - | TGTTTGCCTTT |
| MA0512.1 | MA0512.1.Rxra | 12.46461 | 0.94387 | NC_000068.7:c155076497-155074398 | 1876 | 1886 | + | CAAAGGCCACA |
| MA0512.1 | MA0512.1.Rxra | 12.26563 | 0.941004 | NC_000068.7:c155076497-155074398 | 1093 | 1103 | + | CTGAGGTCATG |

**Supplementary Table S7. List of abbreviations.**

| **Abbreviations** | **Full names** |
| --- | --- |
| IF | Intermittent fasting |
| MASLD | Metabolic dysfunction-associated steatotic liver disease |
| HFD | High-fat diet |
| Ahcy | S-adenosylhomocysteine hydrolas |
| SE | Super-enhancer |
| Brd4 | Bromodomain containing 4 |
| Hnf4a | Hepatic nuclear factor 4a |
| SAM | S-adenosylmethionine |
| SAH | S-adenosylhomocysteine |
| MASH | Metabolic dysfunction-associated steatohepatitis |
| PCK1 | Phosphoenolpyruvate carboxykinase 1 |
| CYP8B1 | Cytochrome P450 family 8 subfamily b polypeptide 1 |
| Srebf1 | Sterol regulatory element binding transcription factor 1 |
| Fasn | Fatty acid synthase |
| Scd1 | Stearoyl-Coenzyme A desaturase 1 |
| Il6 | Interleukin 6 |
| Tnfa | Tumor necrosis factor a |
| Il1b | Interleukin 1 beta |
| DEGs | Differentially expressed genes |
| KEGG | Kyoto Encyclopedia of Genes and Genomes |
| GO | Gene Ontology |
| GSEA | Gene Set Enrichment Analysis |
| BET | Bromine domain and terminal outer domain |
| TF | Transcription factor |
| DMR | Differential methylation regions |
| Acot12 | Acyl-CoA thioesterase 12 |
| MSP | Methylation-specific PCR |
| Dnmt | DNA methyltransferase |
| TG | Triglycerides |
| TC | Total cholesterol |
| AST | Aspartate Aminotransferase |
| ALT | Alanine Aminotransferase |

**Supplementary Methods**

**Extraction of primary cells from mouse liver**

Mice were anesthetized with 1.25% 2,2,2-tribromoethanol (30 μL/g) by intraperitoneal injection. After anesthesia, the mice were routinely restrained and placed in a biosafety cabinet. The abdomen was disinfected with 75% alcohol, and a cross-shaped incision was made to open the abdominal cavity, fully exposing the portal vein and inferior vena cava. A cannula needle was inserted into the portal vein to inject the pre-perfusion solution, and the inferior vena cava was cut to allow blood drainage. Then, the enzyme digestion perfusion solution (post-perfusion solution, 10-12 mL/min) was used for perfusion. After the perfusion, the liver was immersed in a 10cm culture dish containing digestion medium. The cells flowed out when the liver capsule was uncovered, and the liver capsule and connective tissue were removed. The liver tissue was then minced rapidly and gently pipetted several times with a 10 mL pipette to ensure thorough mixing. Complete culture medium was added to terminate digestion. The obtained cell suspension was filtered through a 70-75μm sieve. The suspension was centrifuged at 50g for 5 minutes at 4℃, and the supernatant was discarded. After resuspending in 20ml of culture medium, centrifugation was repeated under the same conditions, and the supernatant was discarded. Then, 20ml of culture medium was added for resuspension, followed by centrifugation at 200g for 5 minutes at 4℃. After resuspending in 20ml of culture medium, centrifugation was repeated under the same conditions, and the supernatant was discarded. The precipitate contained the liver cells.

**Cell oil red O staining**

After the intervention under the corresponding conditions, the culture solution was discarded and the cells were washed with PBS for 3 times. After being fixed with 4% paraformaldehyde for 10-15 min, the cells were washed with PBS for 2-3 times. The cells were stained with freshly filtered Oil Red O staining solution for 10-20 minutes, and wash with PBS twice. The cell nucleus was stained with hematoxylin for 3-5 minutes, washed with tap water to remove excess stain and to develop a blue coloration. The appropriate amount of PBS was added to the cells to uniformly cover them. Then, the cells were observed and photographed under a microscope.

**Cell BODIPY staining**

Cells in the logarithmic growth phase with healthy status were prepared as a cell suspension and inoculated into confocal dishes. The intervention was carried out according to the experimental purpose. After the intervention, the supernatant was discarded, and cells were washed with PBS for 3 times. Cells were fixed with 4% paraformaldehyde at room temperature for 10-15 min, followed by 2-3 washes with PBS. Fluorescent dye BODIPY 558/568 at a concentration of 5 µmol/L was added to the dish and incubated in the dark at 37°C for 15 to 30 minutes. After incubation, the cells were washed 3 times with PBS for 5 minutes each under dark conditions. 500 µL of DAPI solution at a concentration of 1 µg/mL was added to each dish to stain the nuclei, and cells were incubated in the dark for 5 minutes. The dish was placed on a shaker and washed 2 times with PBS for 5 minutes each in the dark. The liquid in the dish was removed, and an appropriate amount of fluorescent anti-quenching agent was added to cover the surface of the dish. Observations and photographs were taken under a confocal fluorescence microscope.

**Molecular Docking**

The Hnf4a protein structure was obtained from AlphaFoldDB via the UniProt database (https://www.uniprot.org/). The tertiary structures of the Ahcy promoter and E3 were modeled using the 3dRNA/DNA web server (http://biophy.hust.edu.cn/new/). Docking of the Hnf4a protein with the corresponding DNA was performed via the HDOCK website (http://hdock.phys.hust.edu.cn/). Analysis and visualization of interactions between amino acid residues and nucleotides within the protein-DNA complex were performed using PyMOL software.
