# Supplementary figures and images for: Ahcy Acts as an Effector of Hnf4a‐Driven Super‐Enhancer Activation to Alleviate MASLD During Intermittent Fasting
Source: Adv Sci (Weinh). 2026 Jul 27:e76826. Online ahead of print. doi: 10.1002/advs.76826 (PMC13403733; doi:10.1002/advs.76826)

A

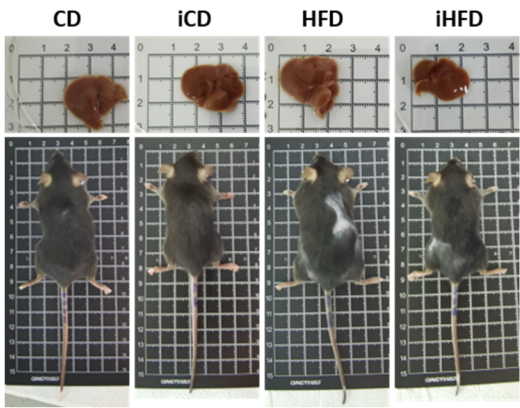

B

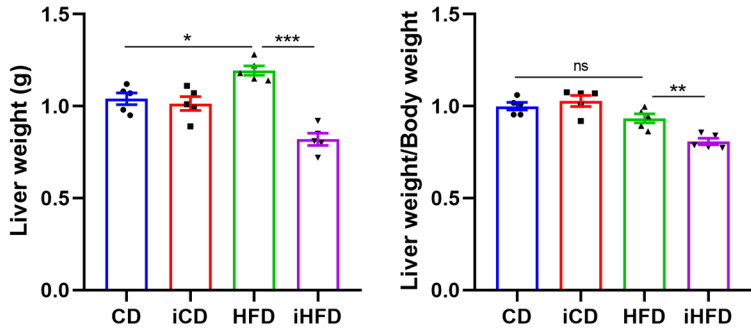

C

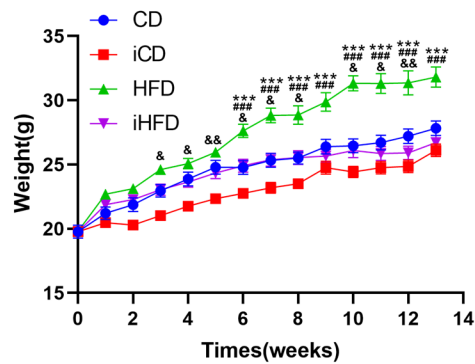

D

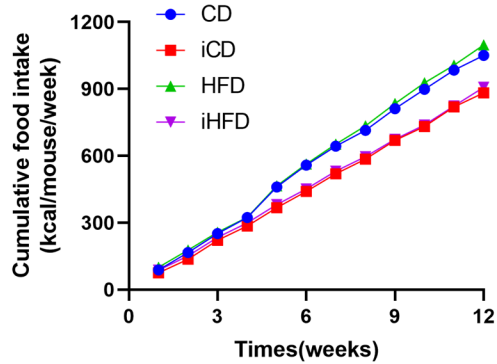

E

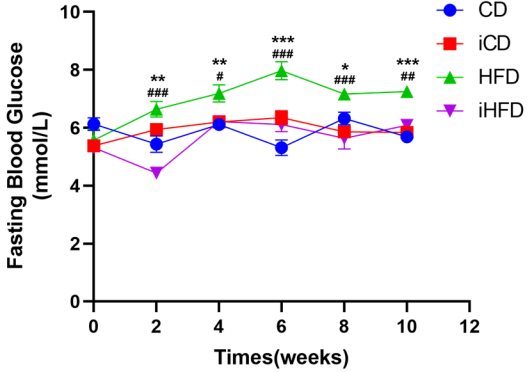

F

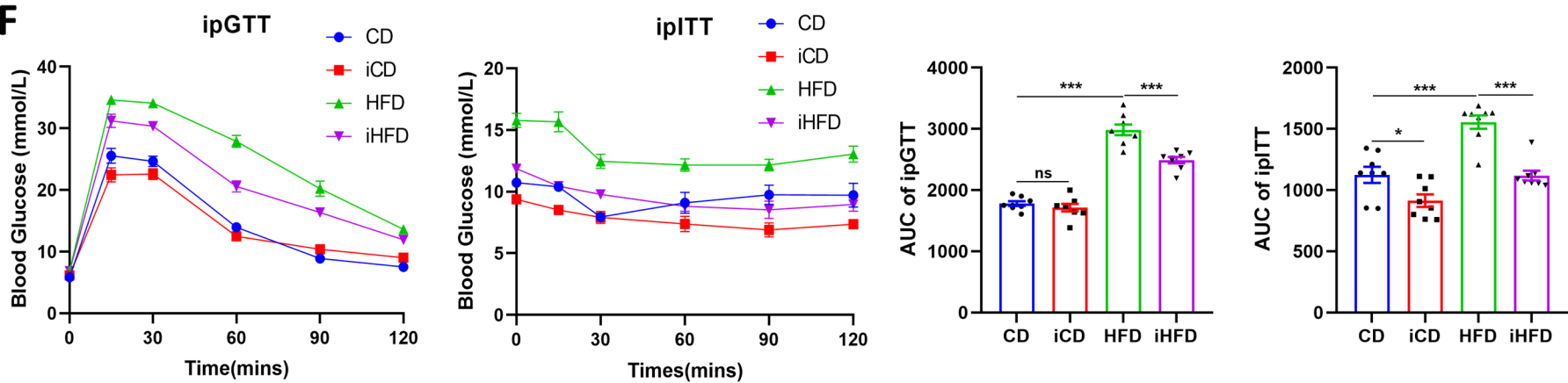

G

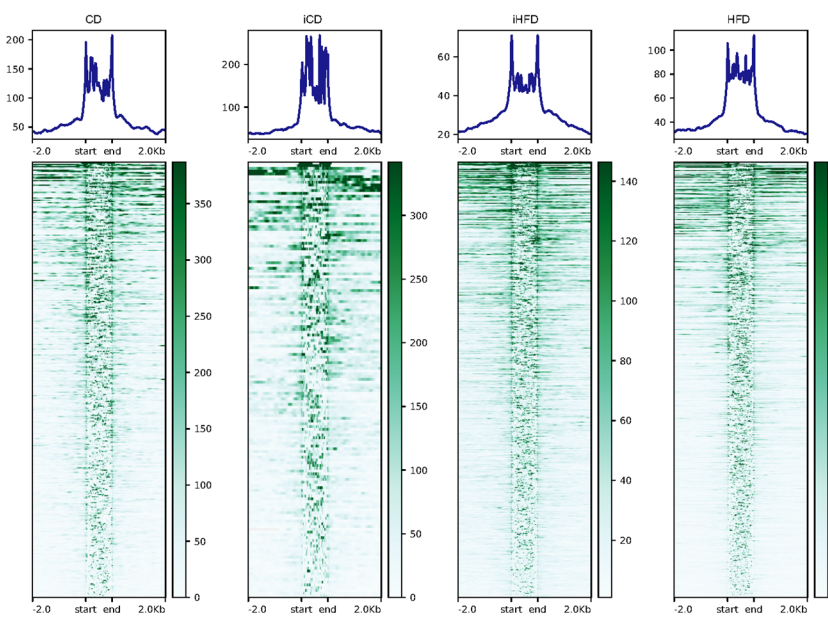

H

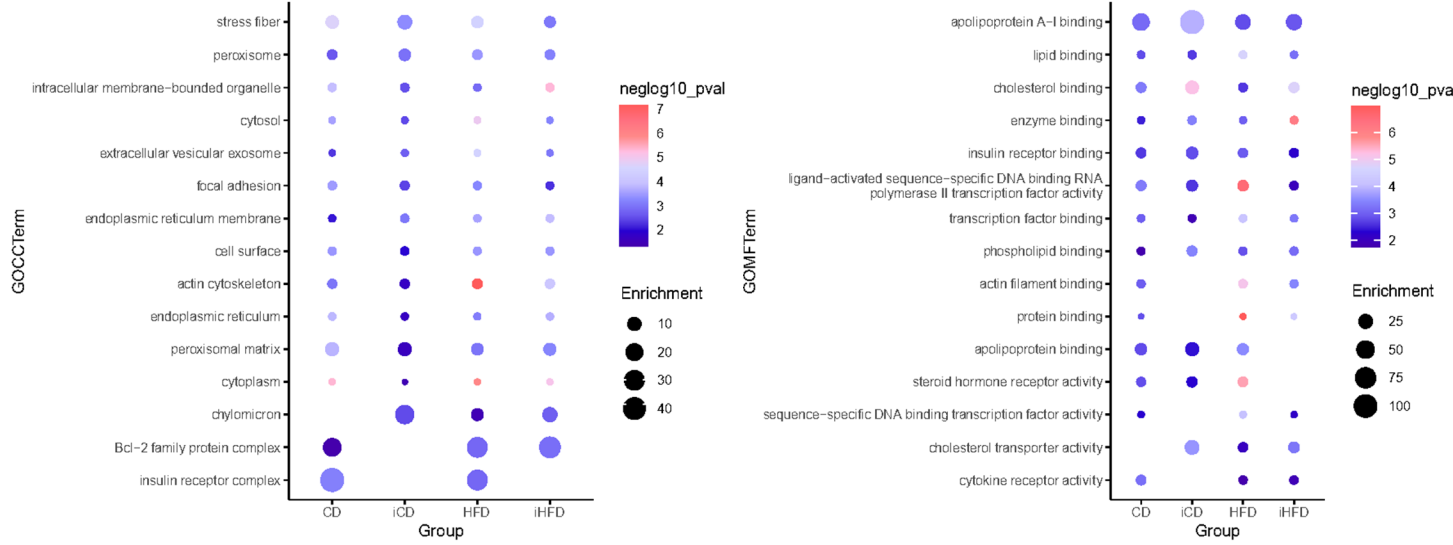

I

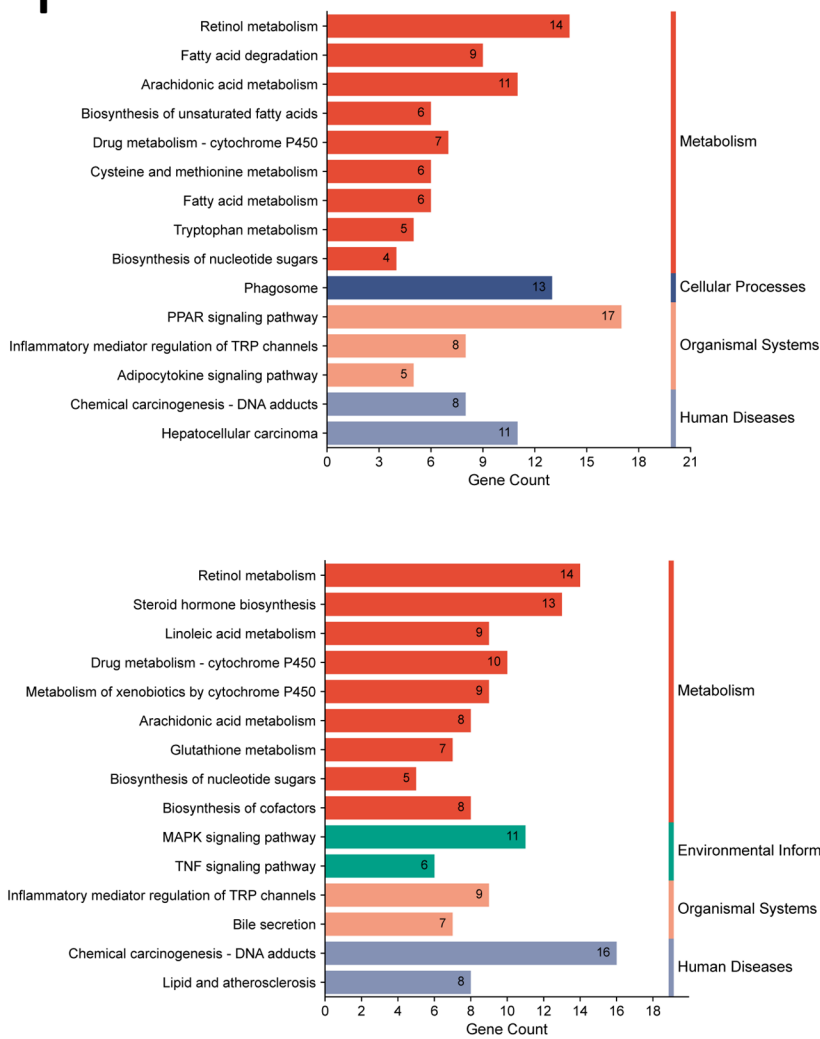

J

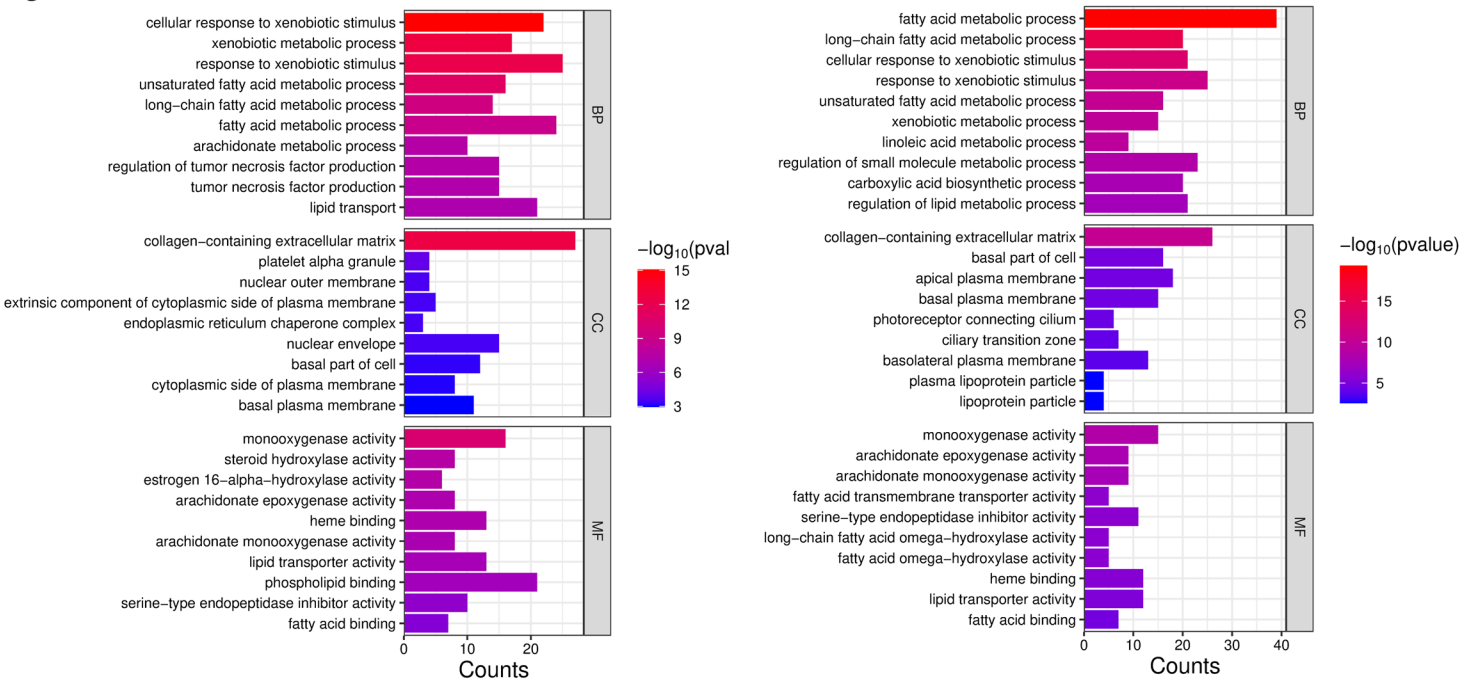

Supplement: Supplementary file 2 — Supporting File 2: advs76826‐sup‐0002‐FigureS1‐S9.zip. [file ADVS-9999-e76826-s001.zip › Supplementary Figure S1.PDF]

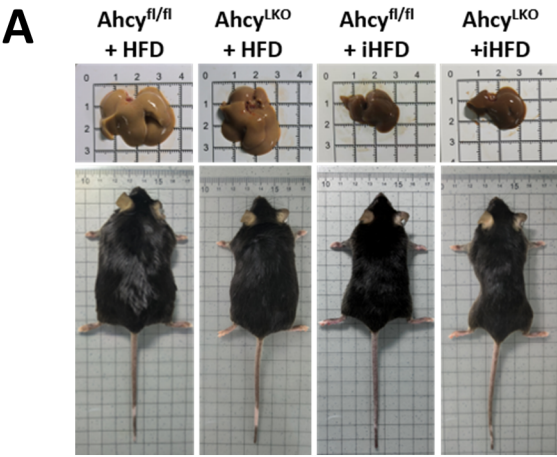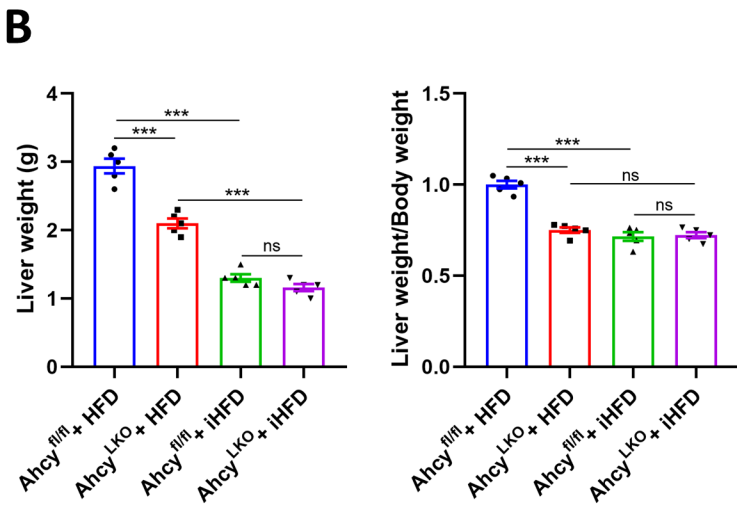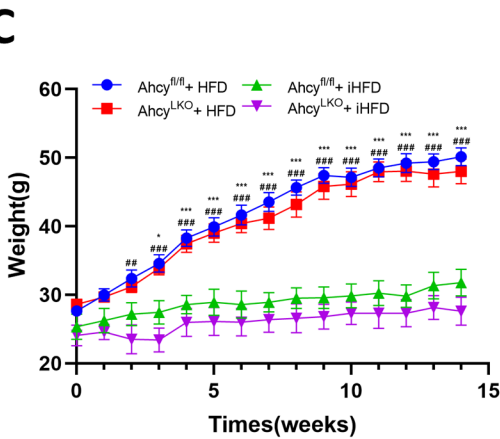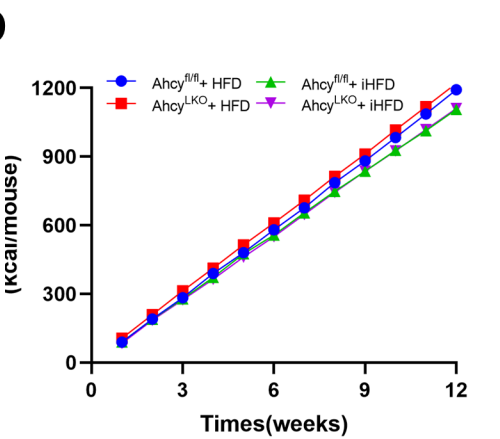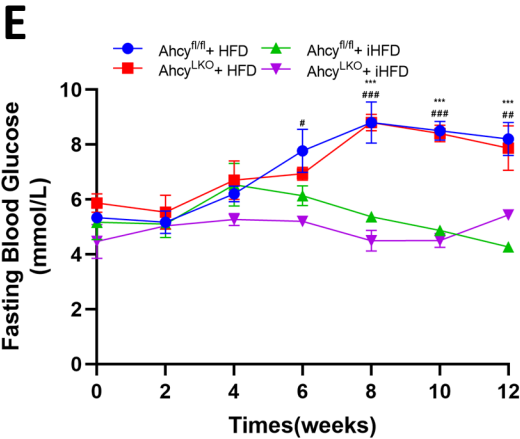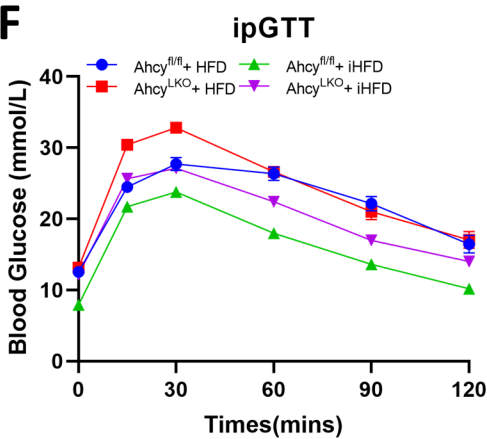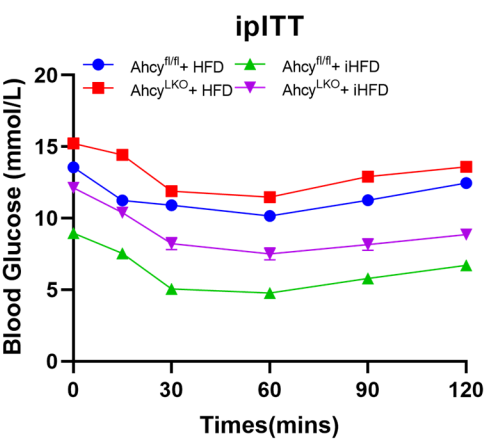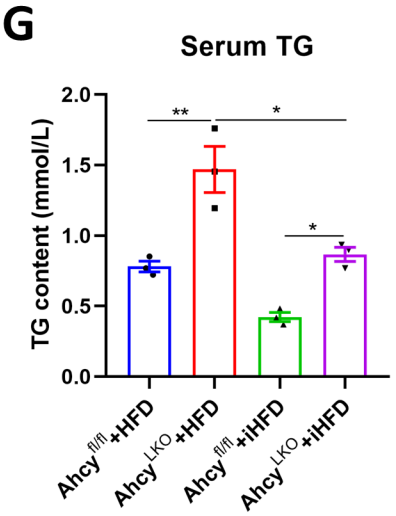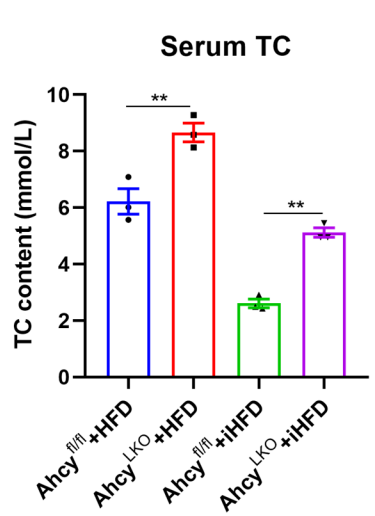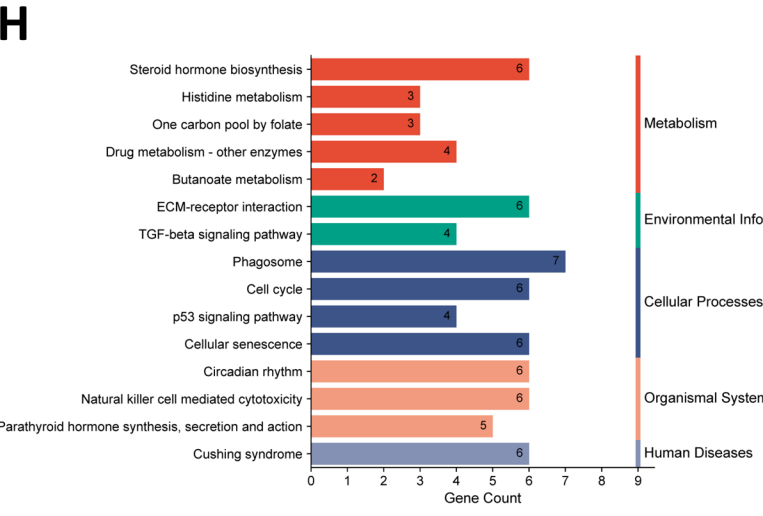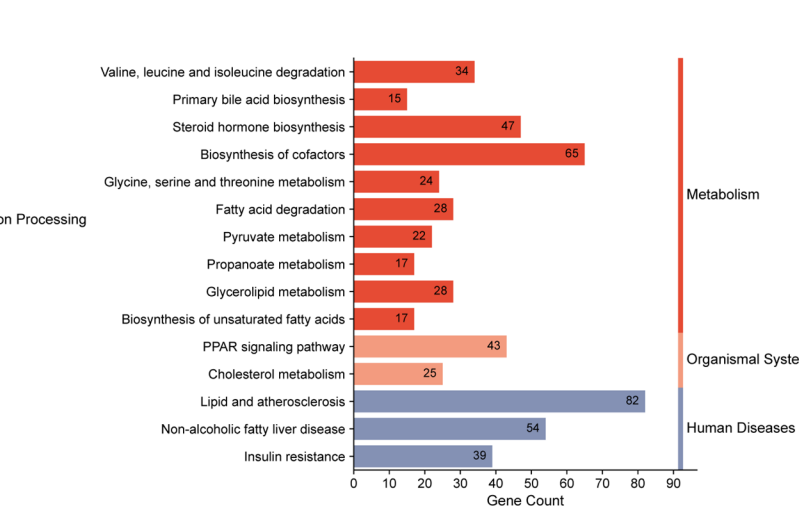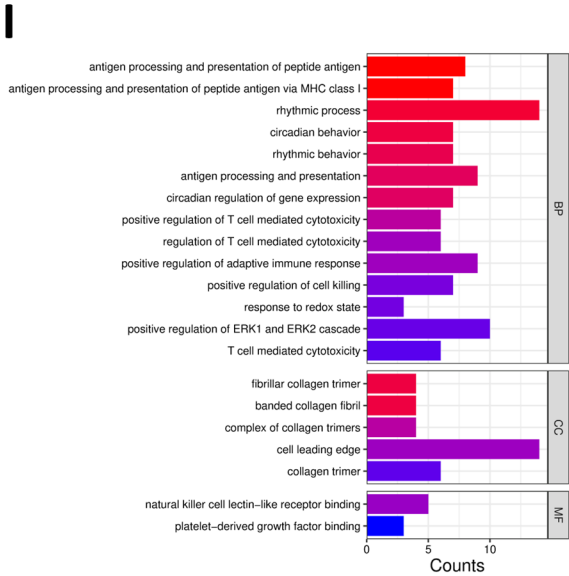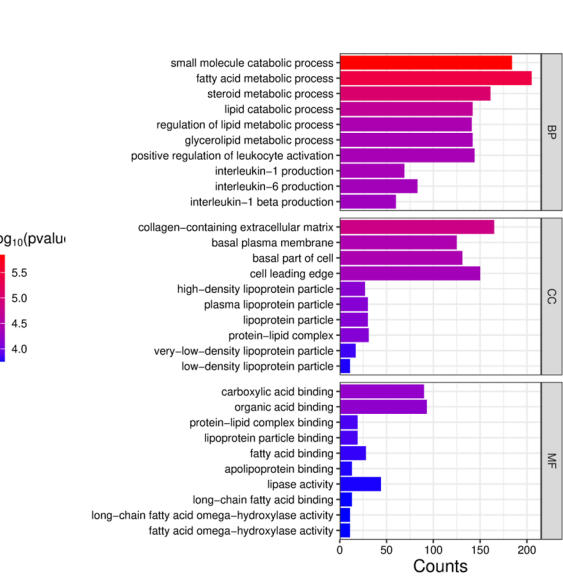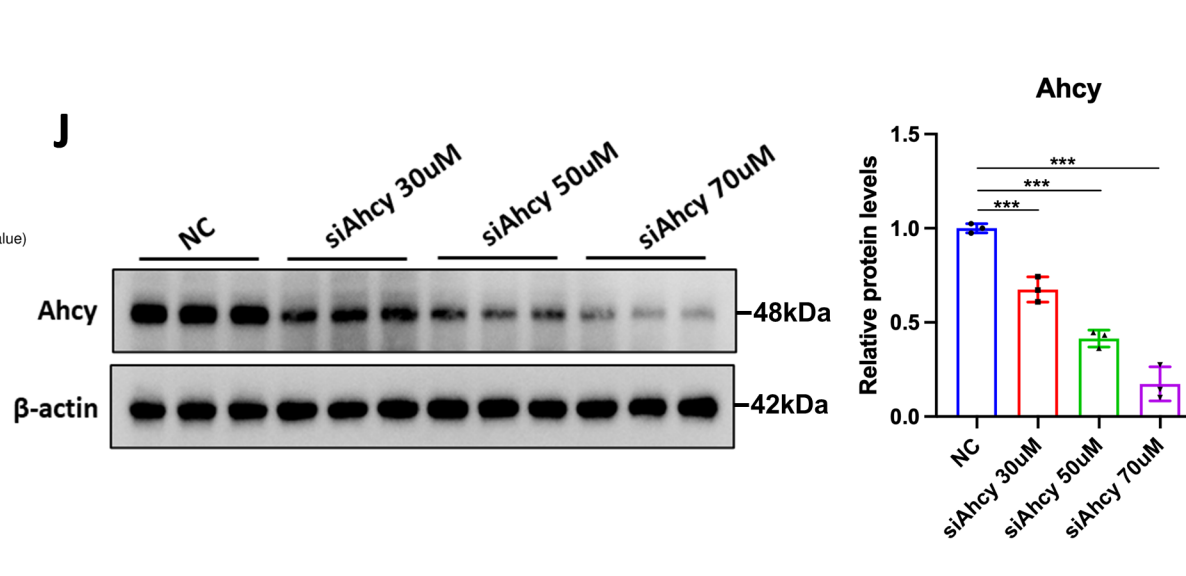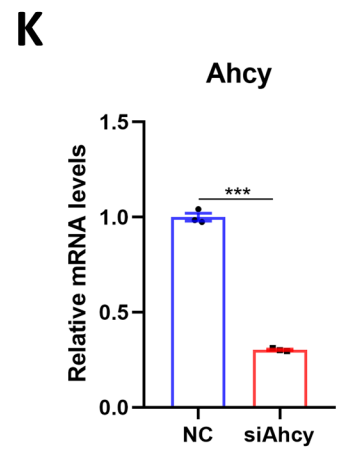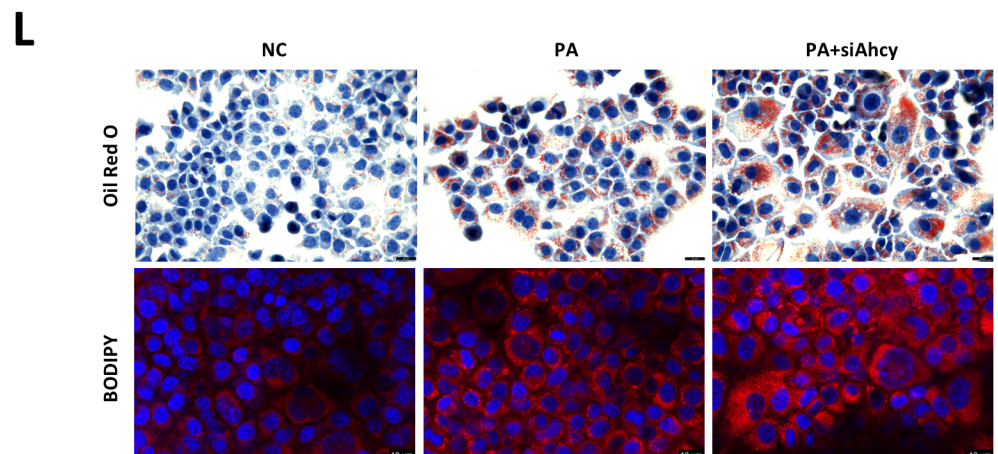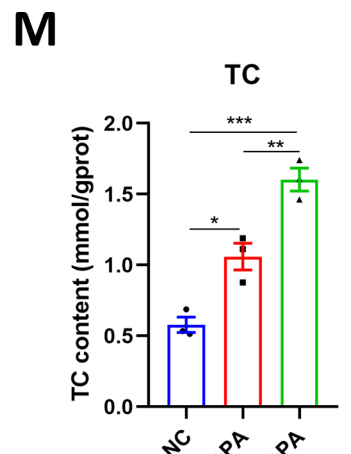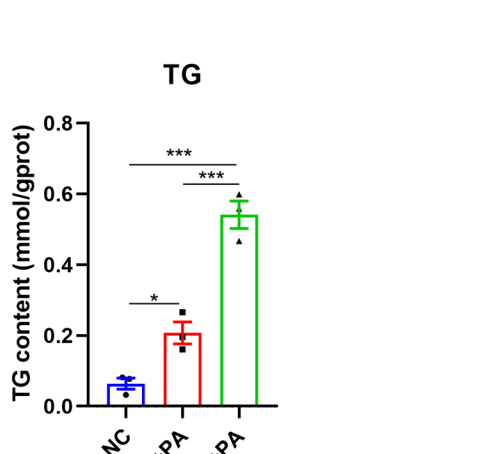

Supplement: Supplementary file 2 — Supporting File 2: advs76826‐sup‐0002‐FigureS1‐S9.zip. [file ADVS-9999-e76826-s001.zip › Supplementary Figure S2.PDF]

**A**

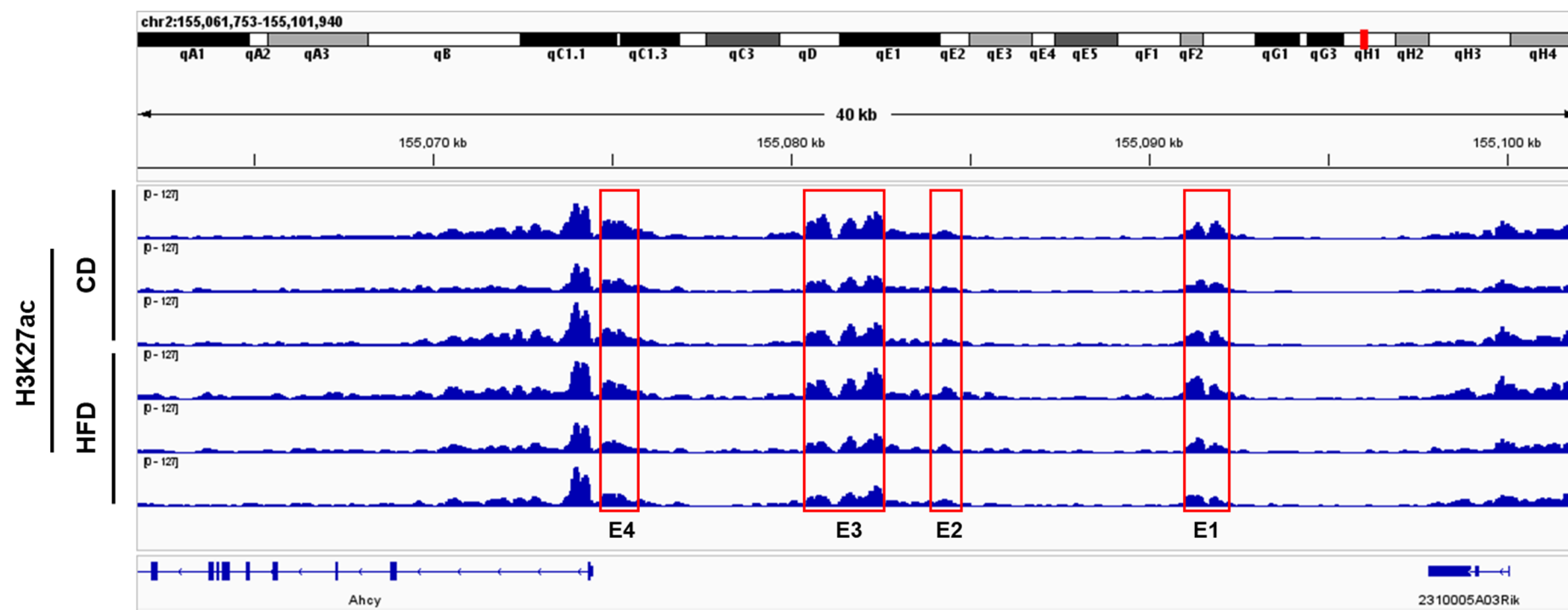

**B**

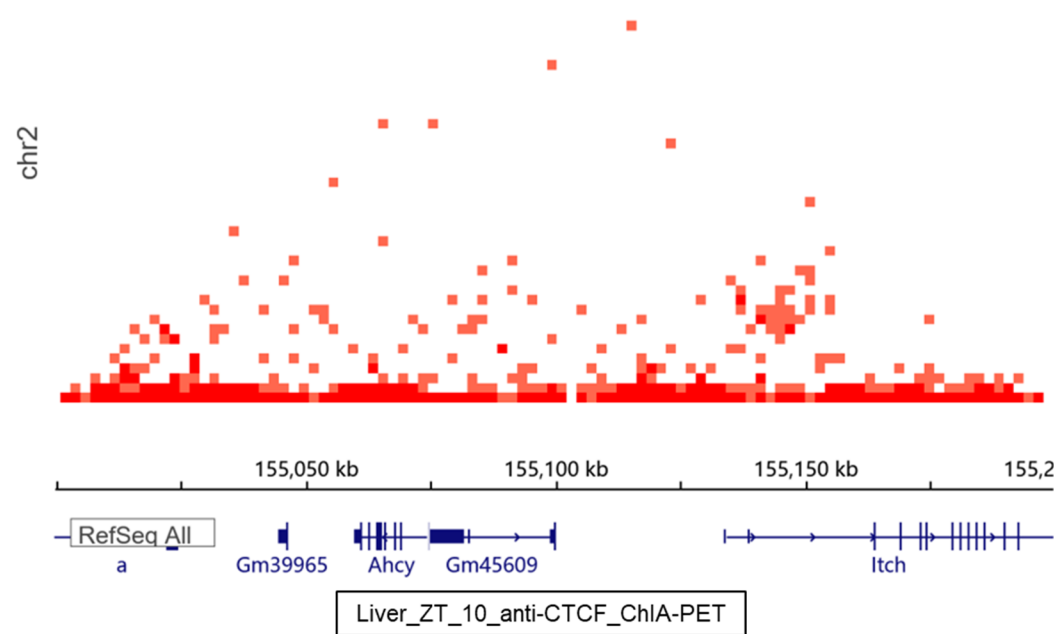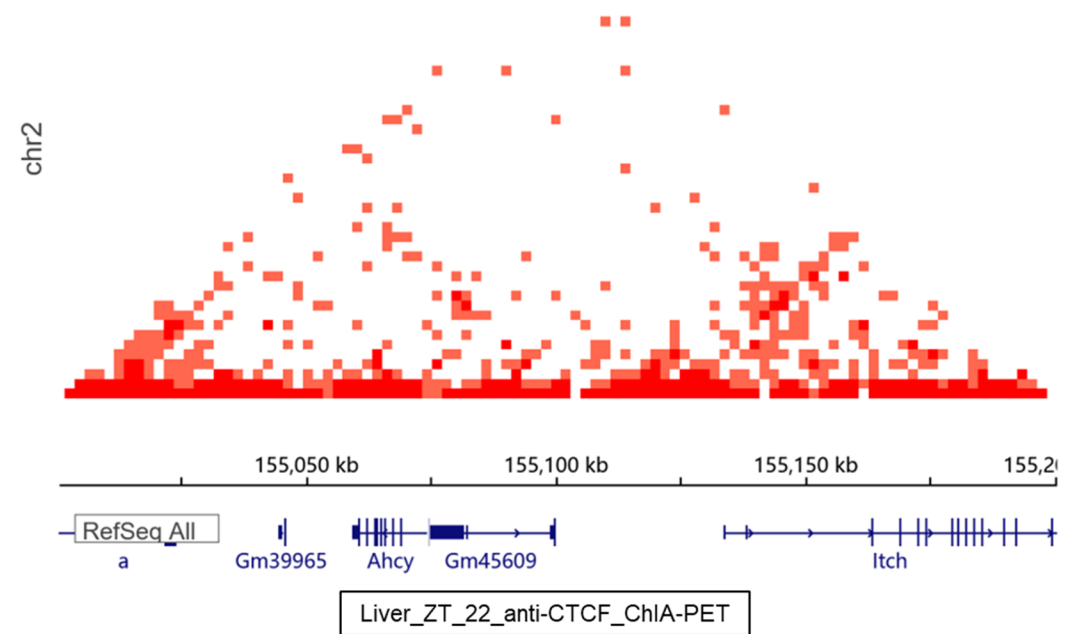

**C Wildtype allele**

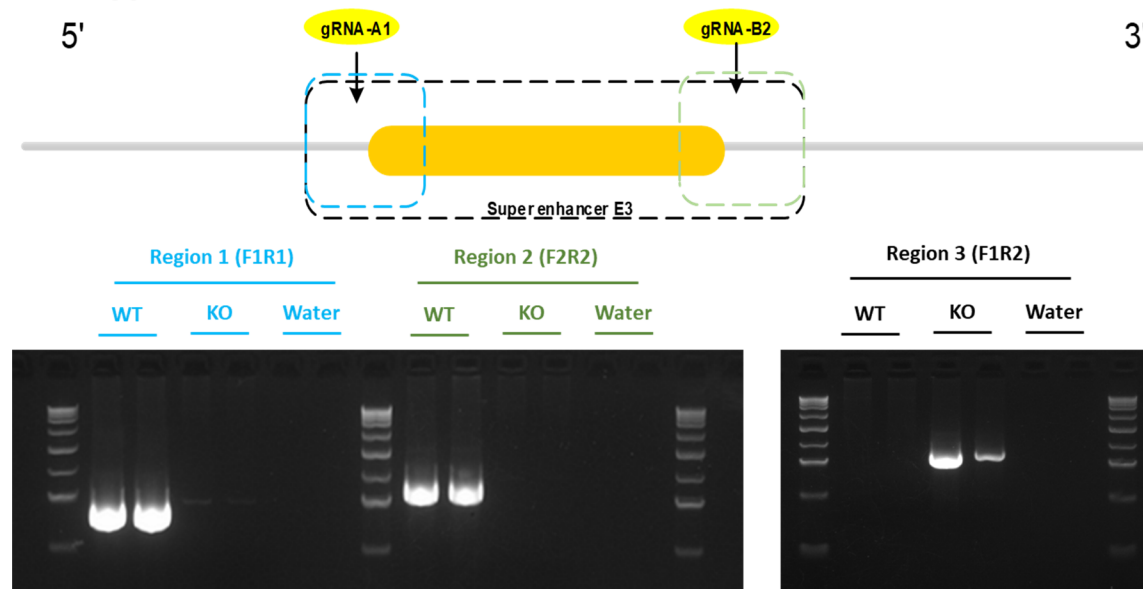

**D**

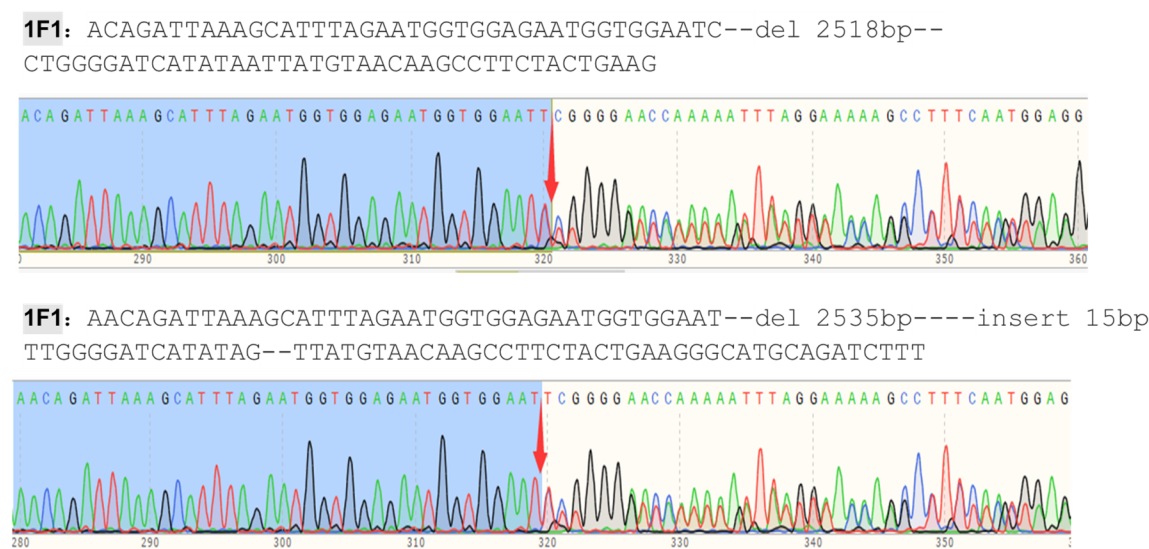

Supplement: Supplementary file 2 — Supporting File 2: advs76826‐sup‐0002‐FigureS1‐S9.zip. [file ADVS-9999-e76826-s001.zip › Supplementary Figure S3.PDF]

A

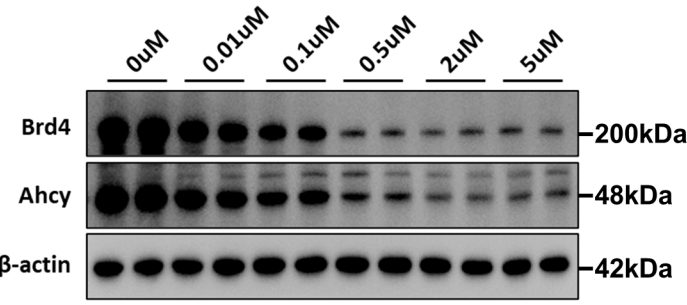

B

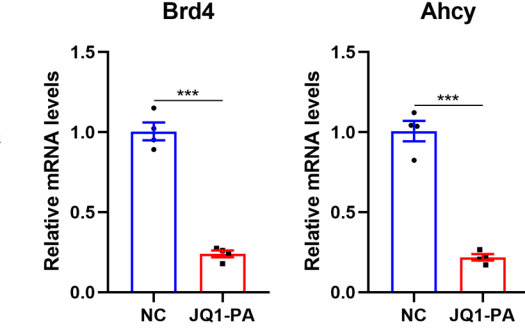

C

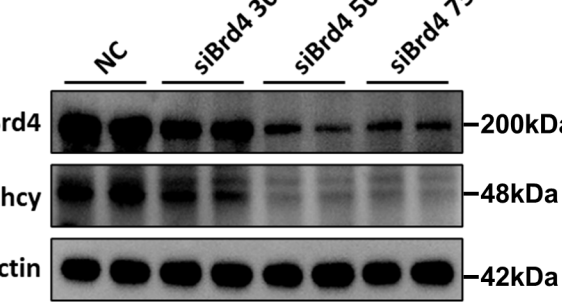

D

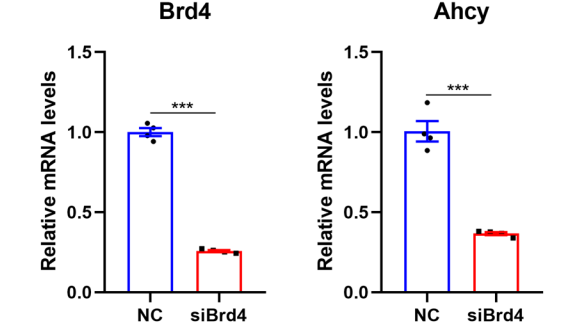

E

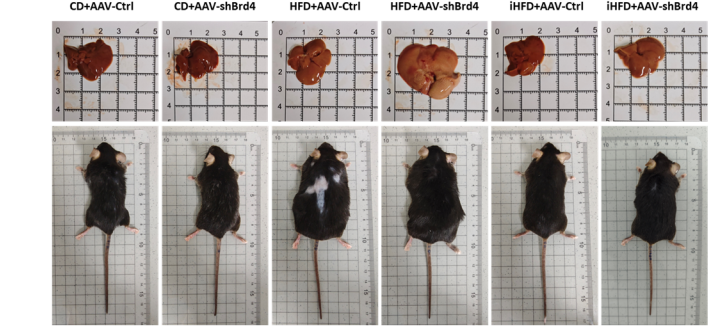

F

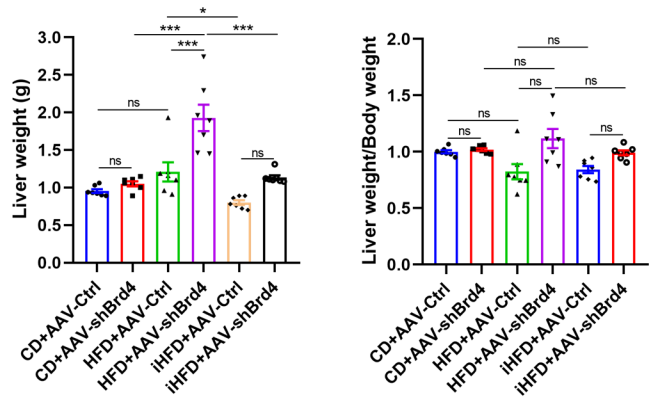

G

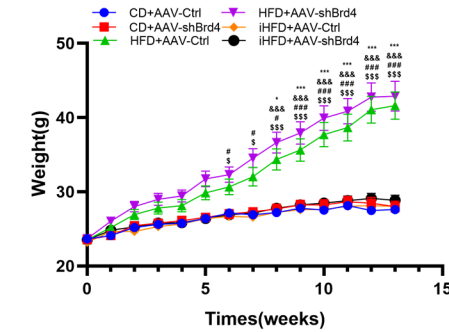

H

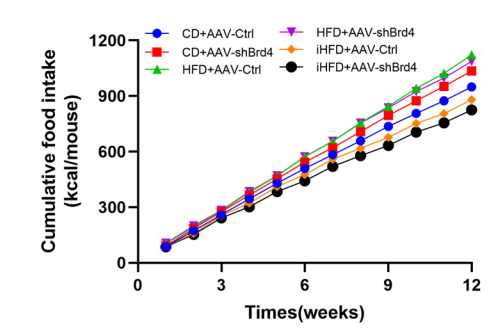

I

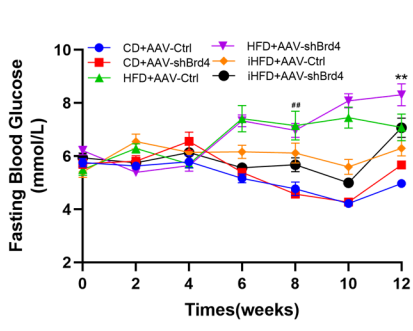

J

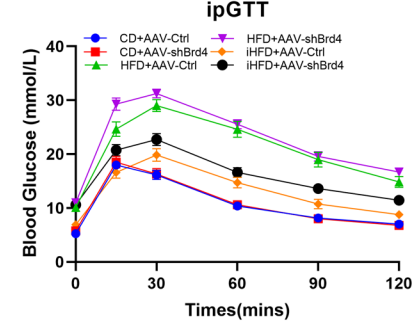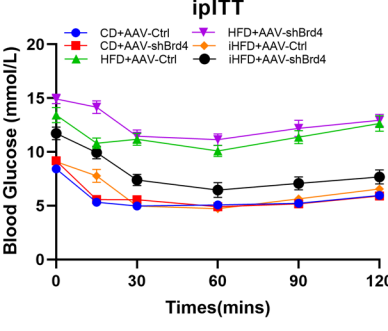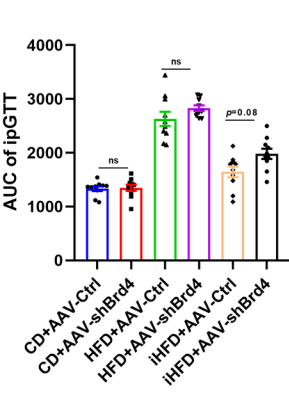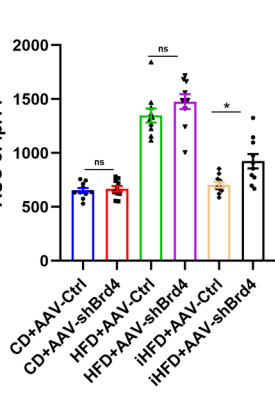

K

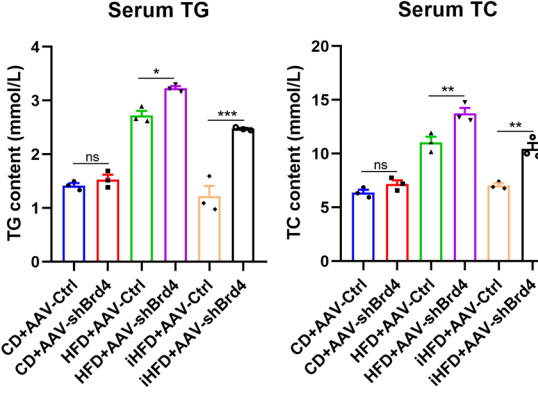

L

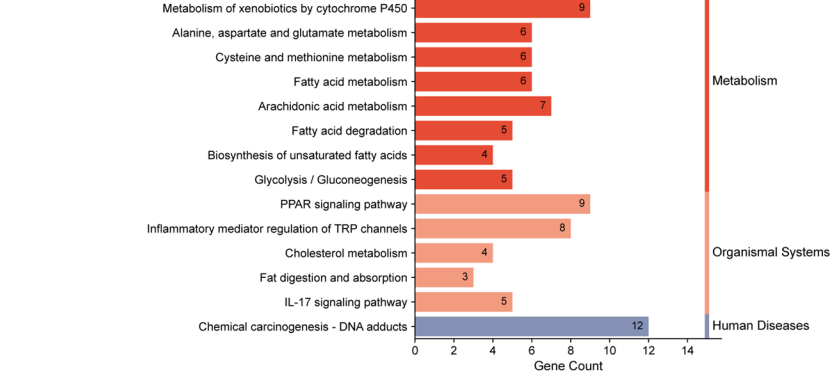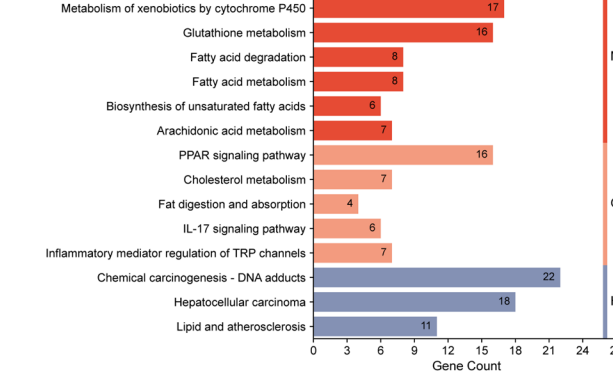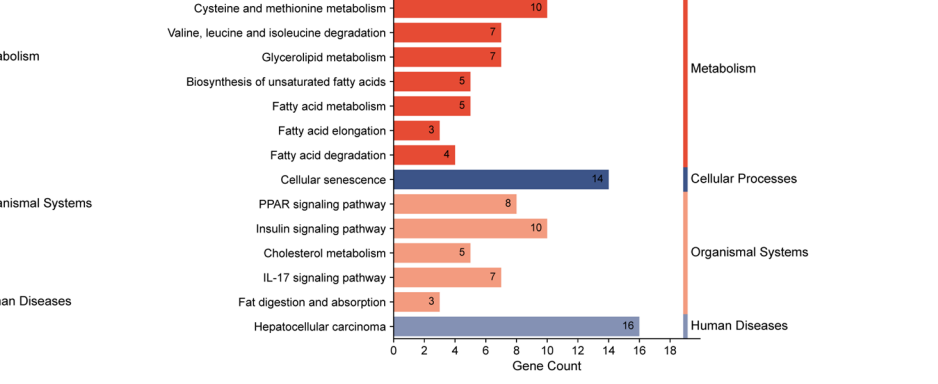

M

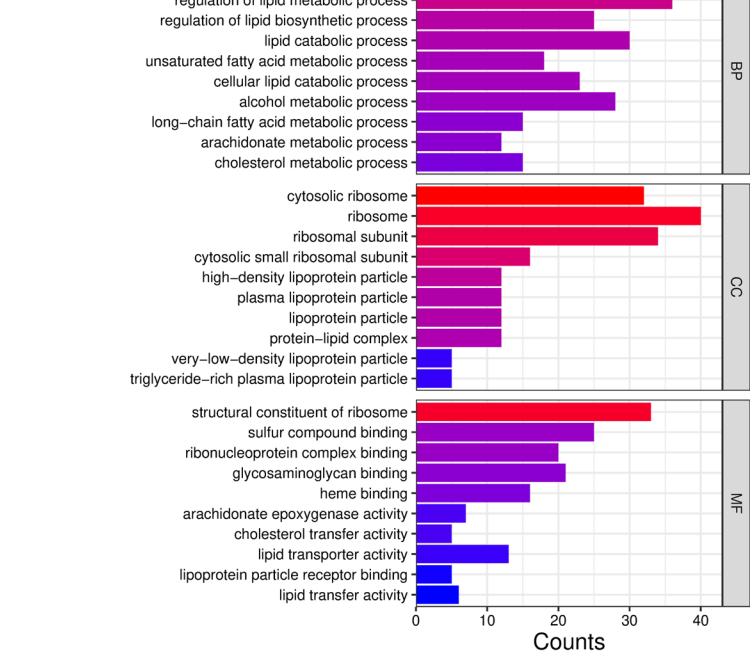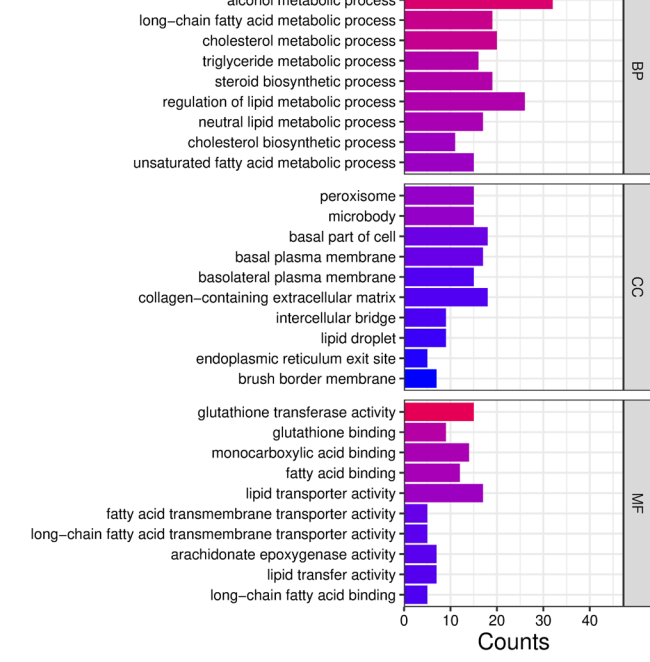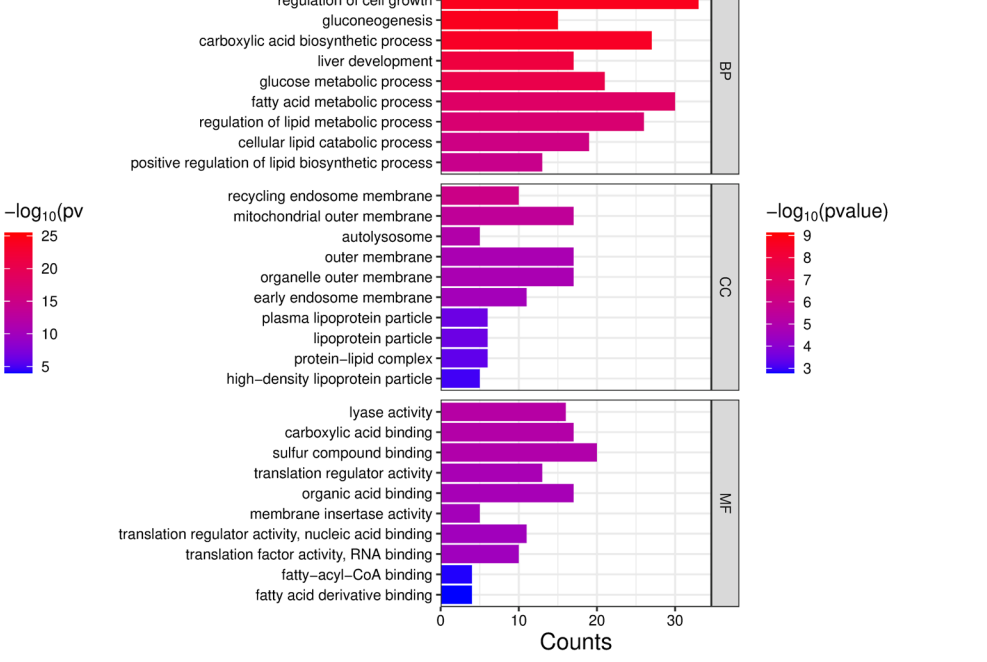

Supplement: Supplementary file 2 — Supporting File 2: advs76826‐sup‐0002‐FigureS1‐S9.zip. [file ADVS-9999-e76826-s001.zip › Supplementary Figure S4.PDF]

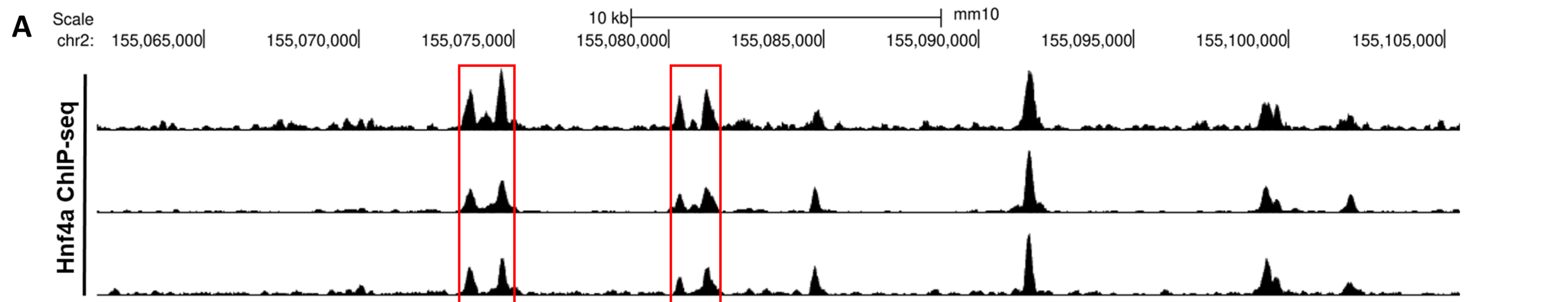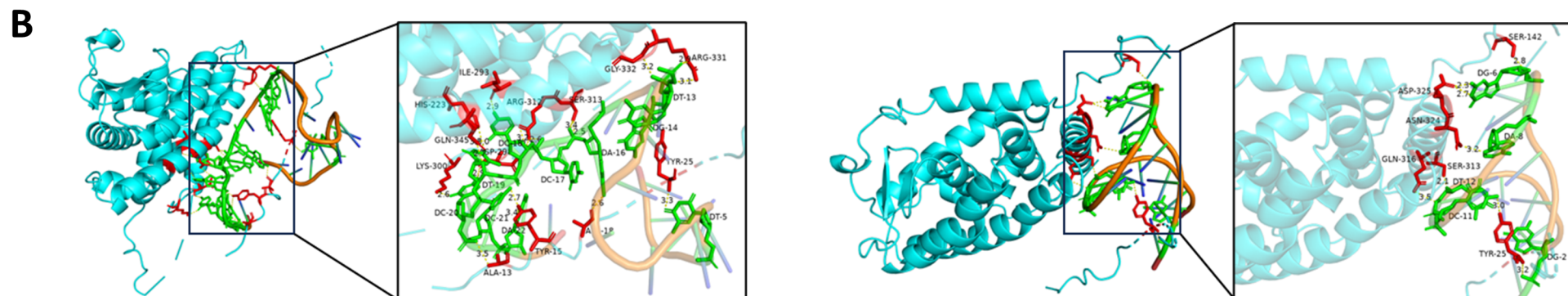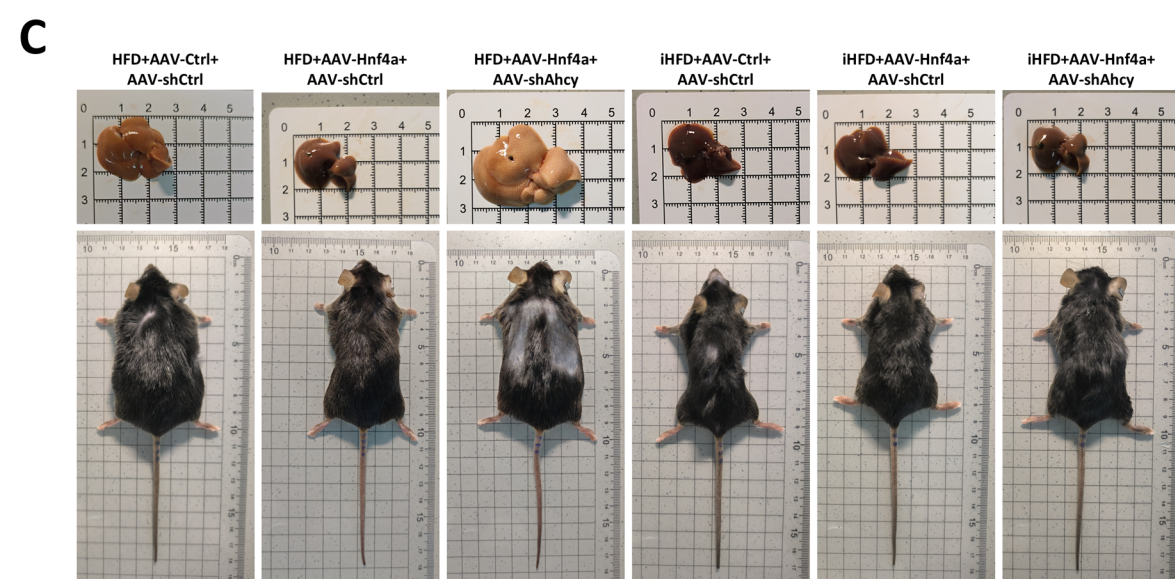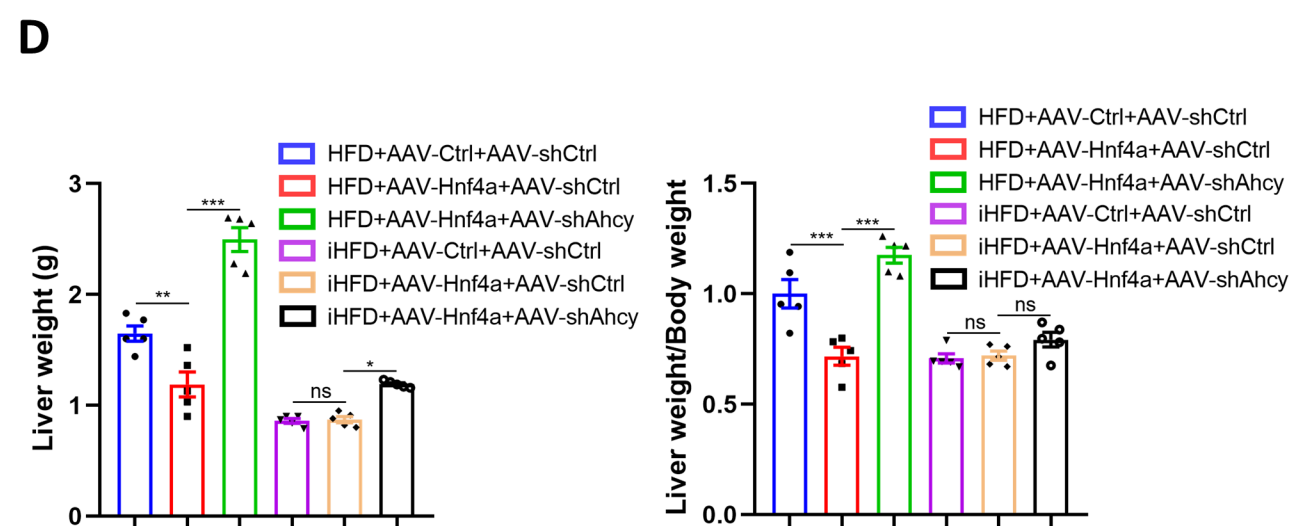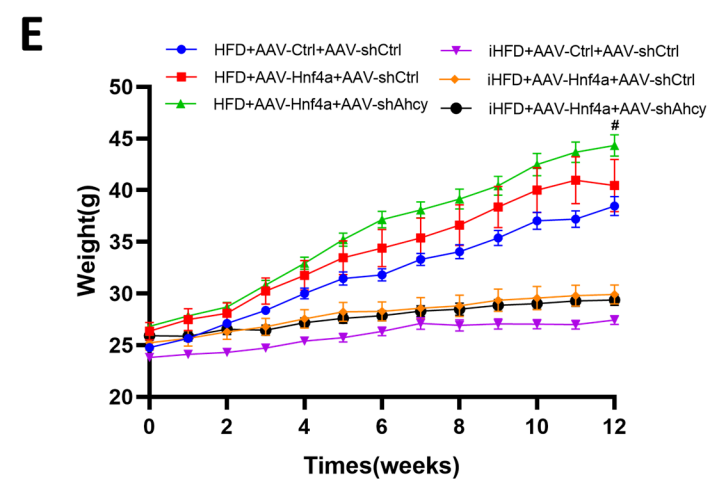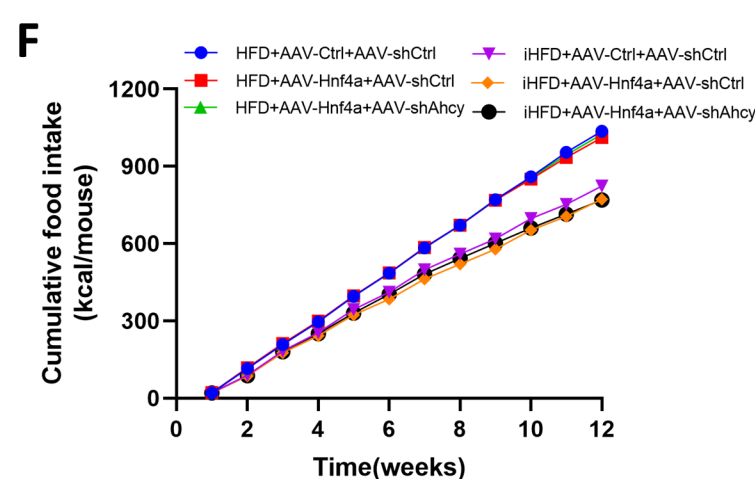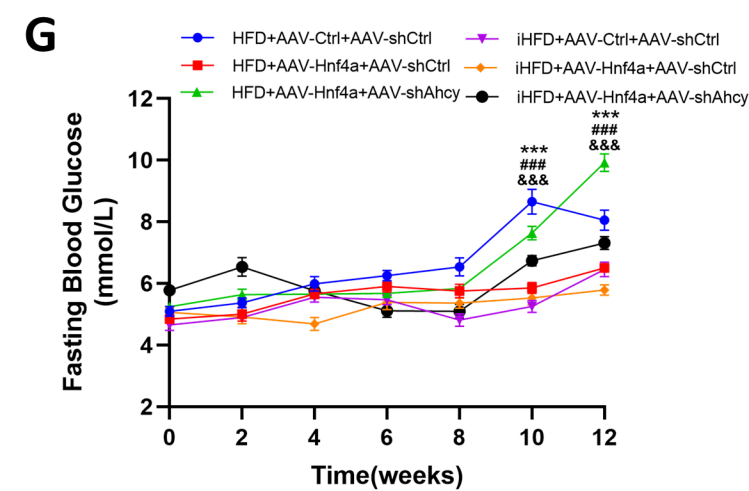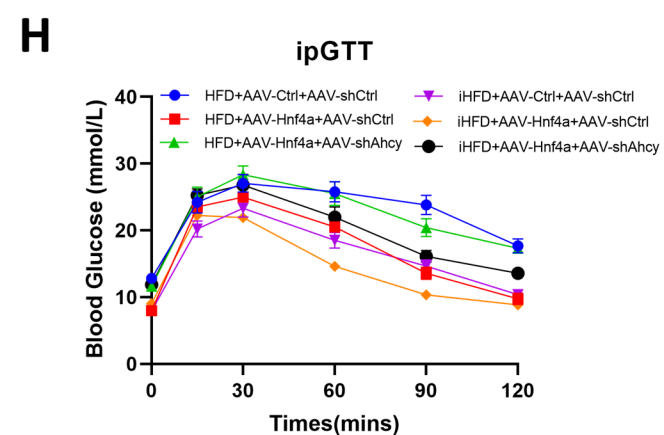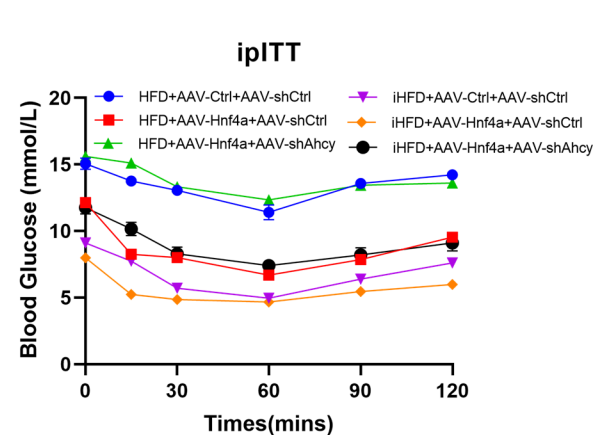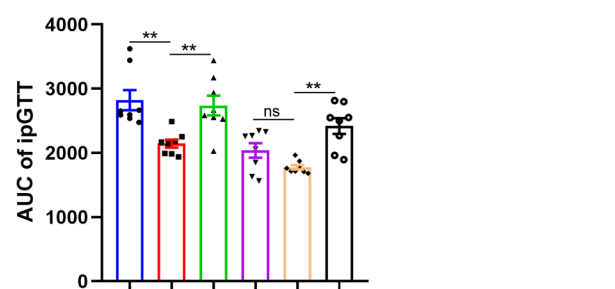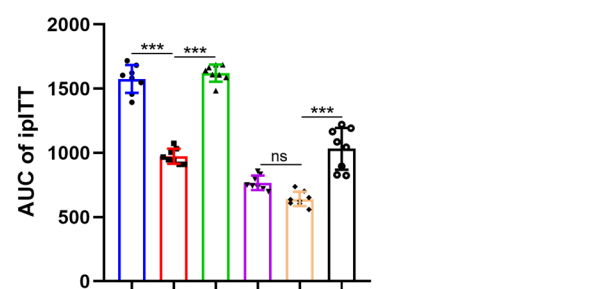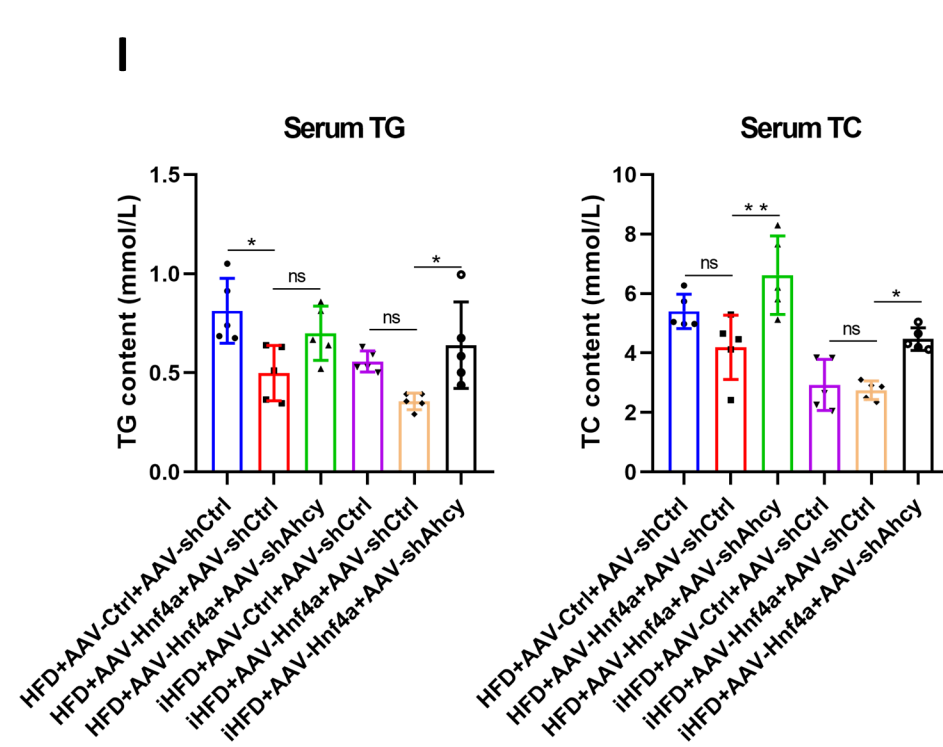

Supplement: Supplementary file 2 — Supporting File 2: advs76826‐sup‐0002‐FigureS1‐S9.zip. [file ADVS-9999-e76826-s001.zip › Supplementary Figure S5.PDF]

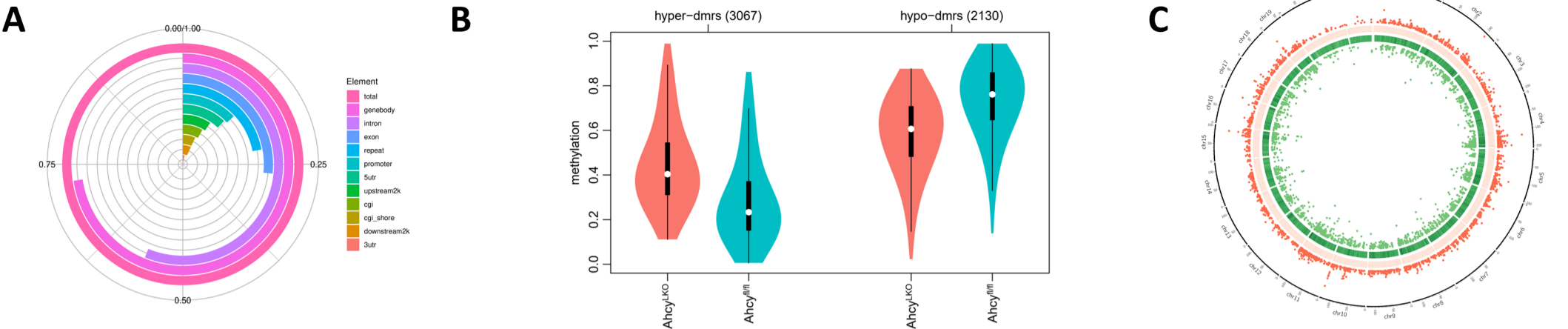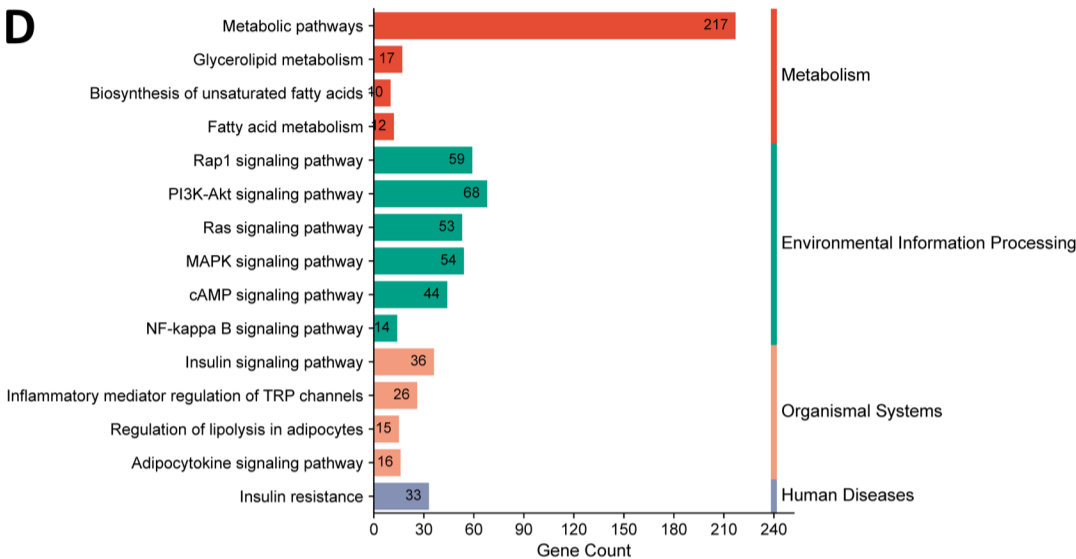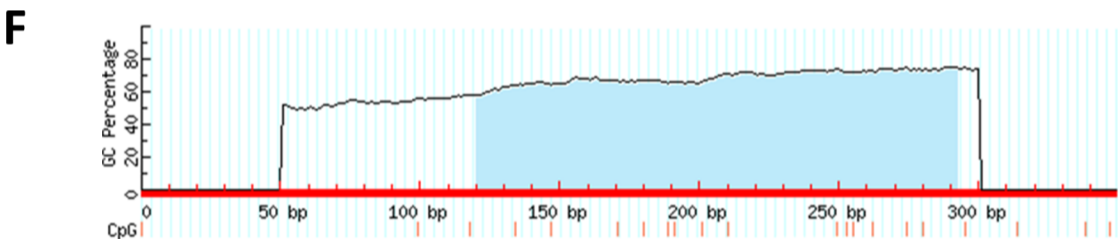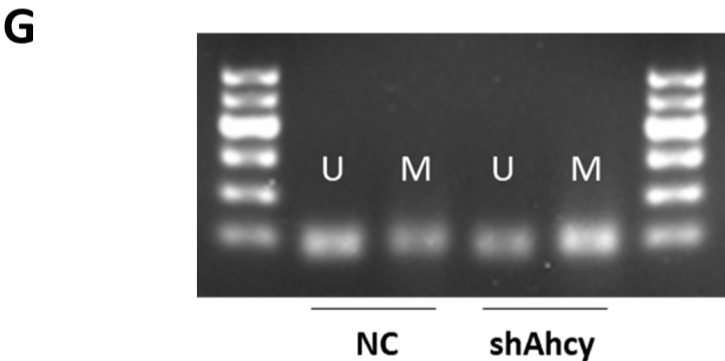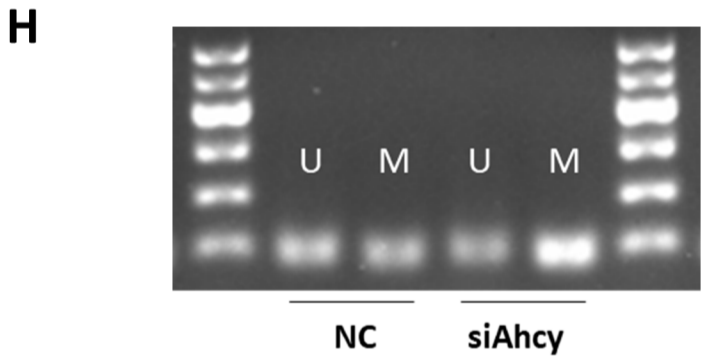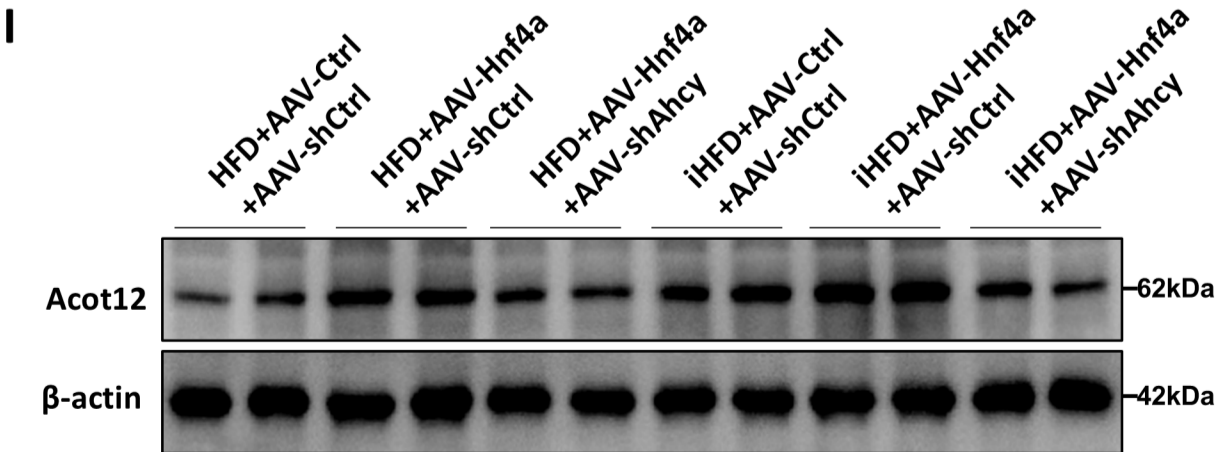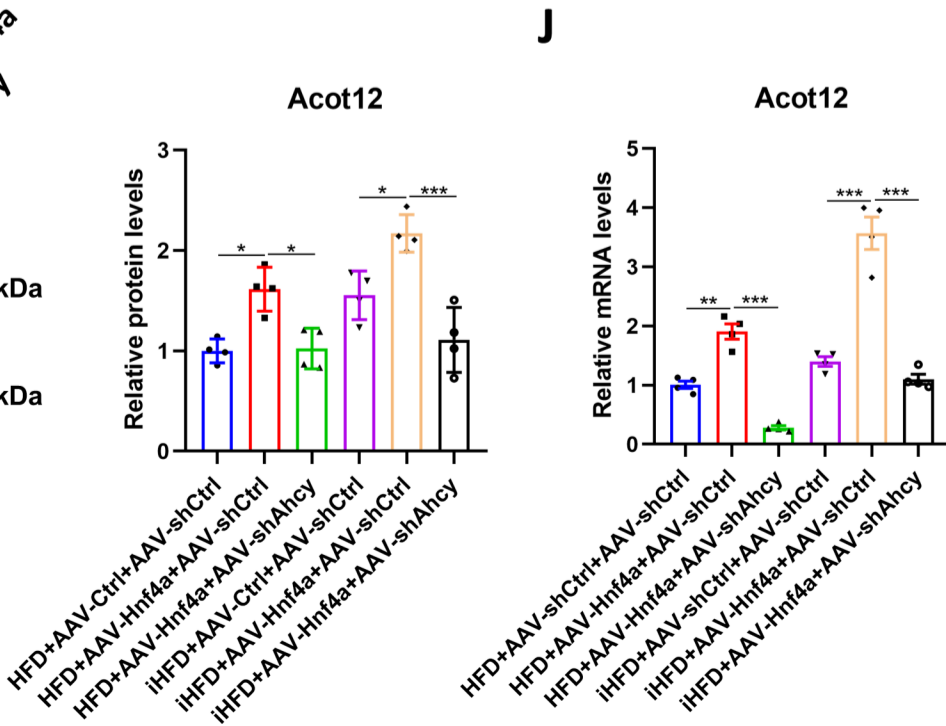

Supplement: Supplementary file 2 — Supporting File 2: advs76826‐sup‐0002‐FigureS1‐S9.zip. [file ADVS-9999-e76826-s001.zip › Supplementary Figure S6.PDF]

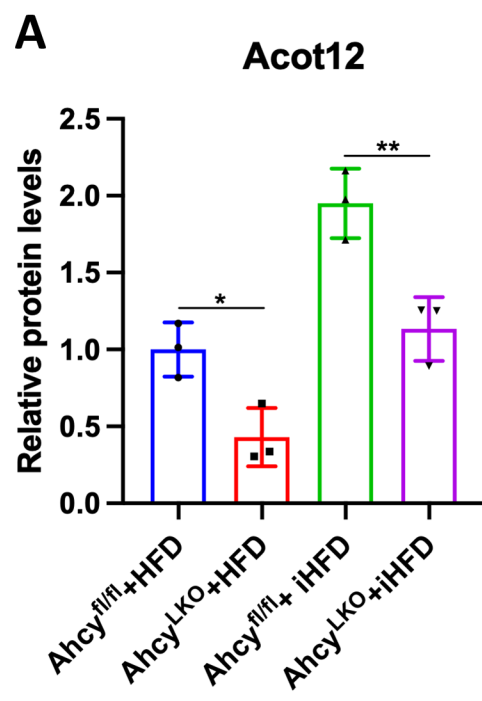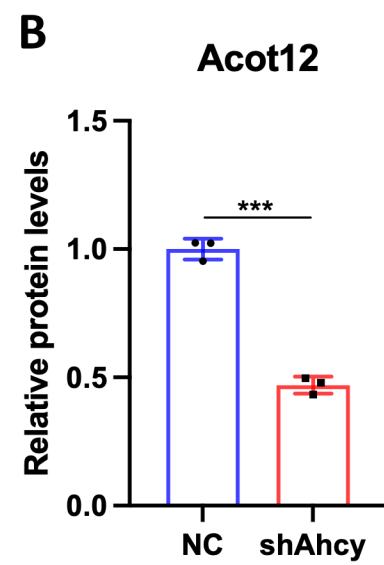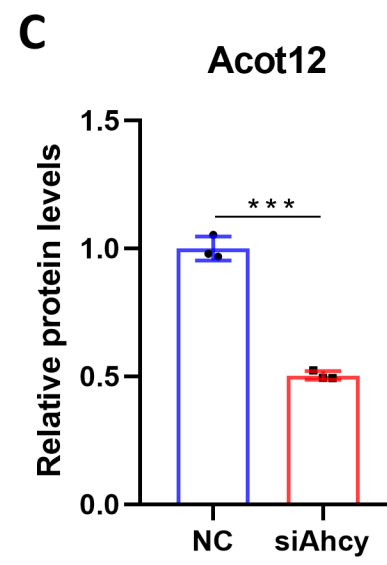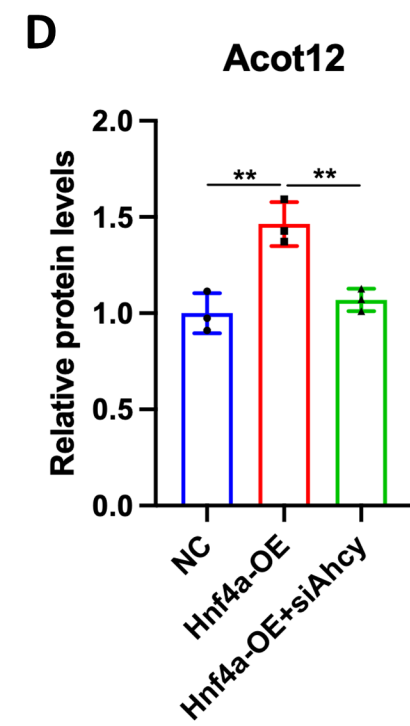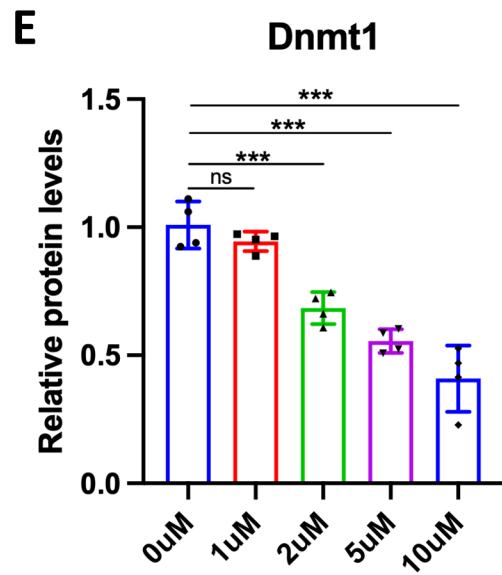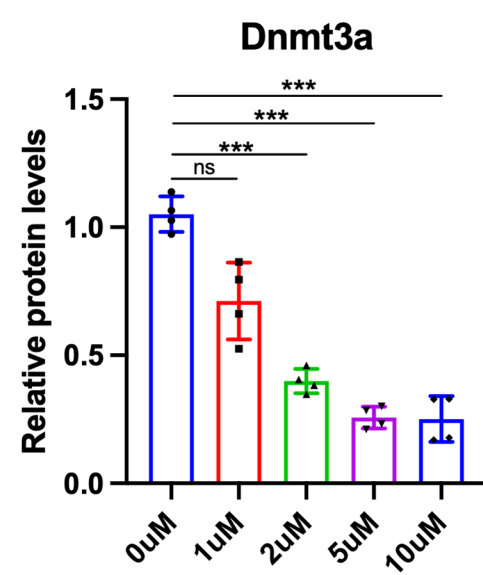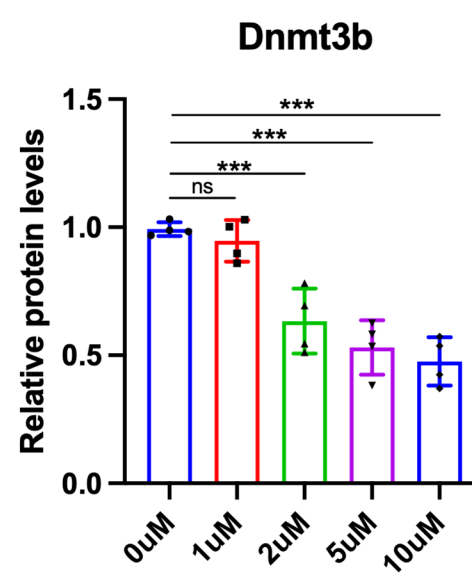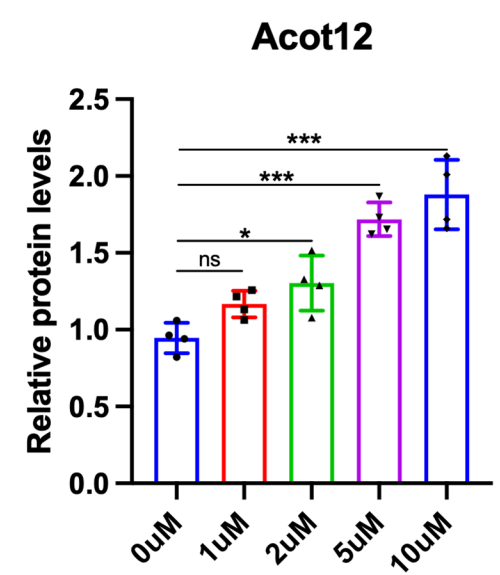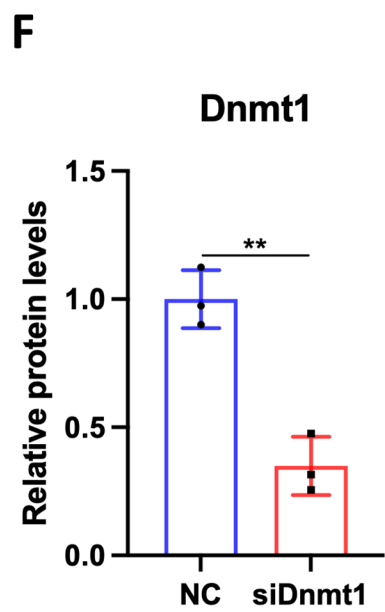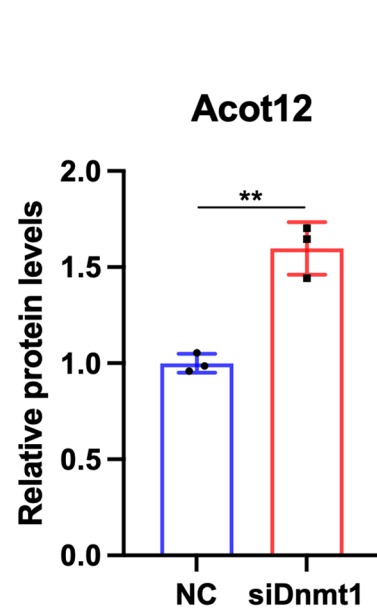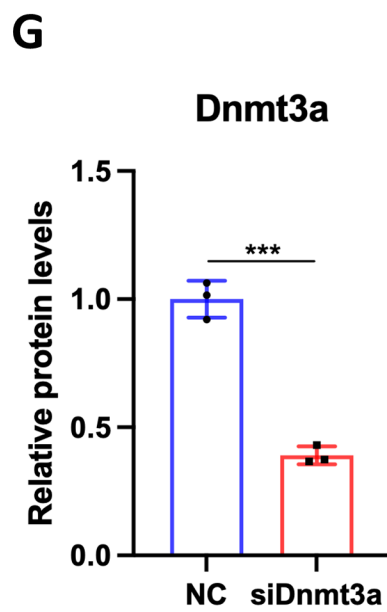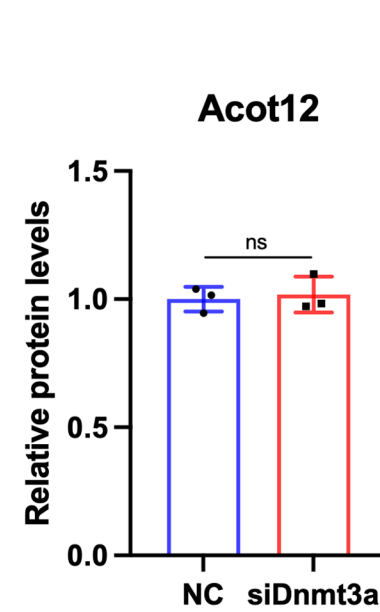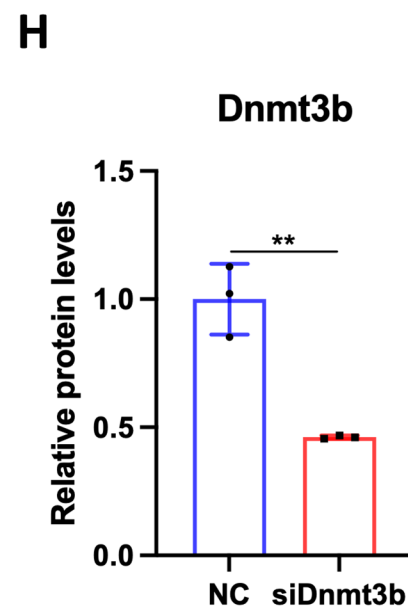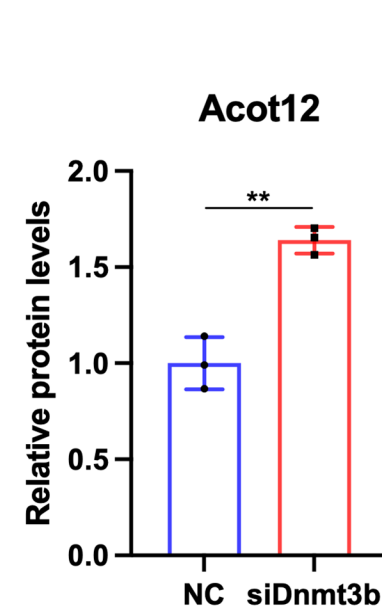

Supplement: Supplementary file 2 — Supporting File 2: advs76826‐sup‐0002‐FigureS1‐S9.zip. [file ADVS-9999-e76826-s001.zip › Supplementary Figure S7.PDF]

**A**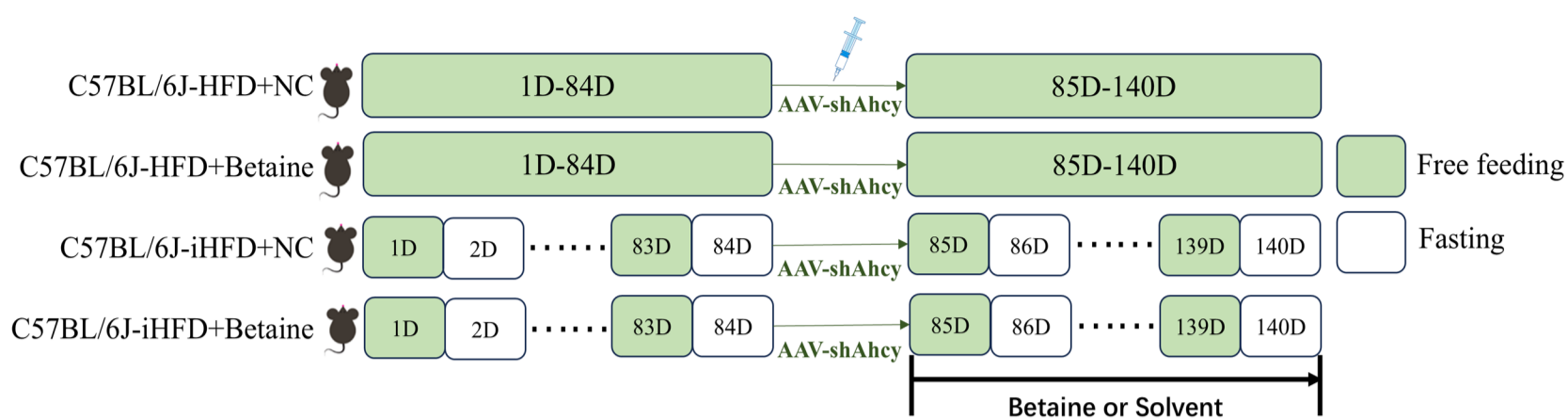**B**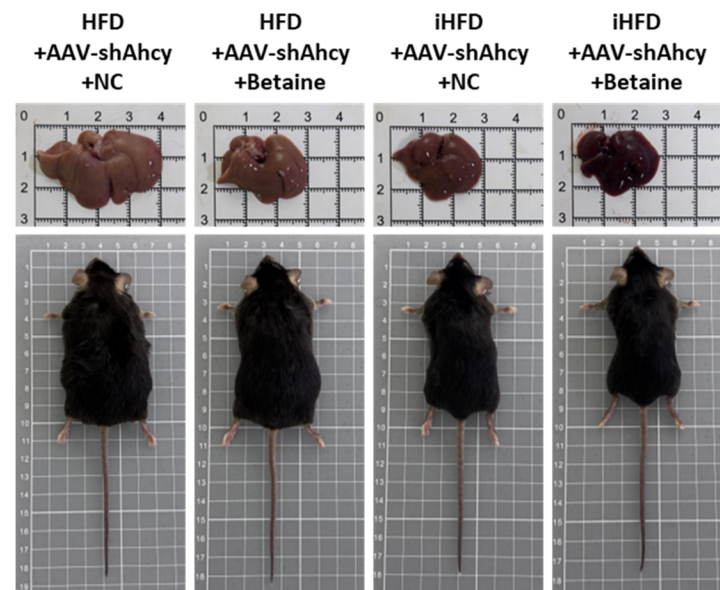**C**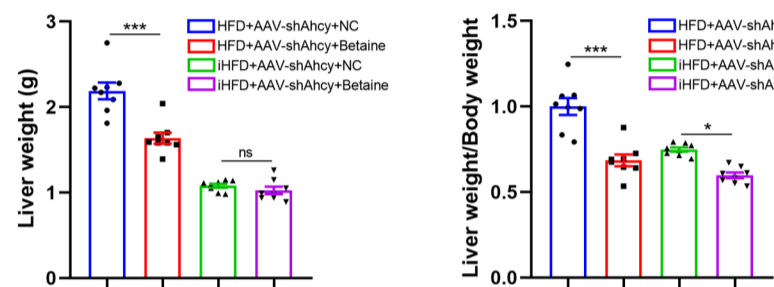**D**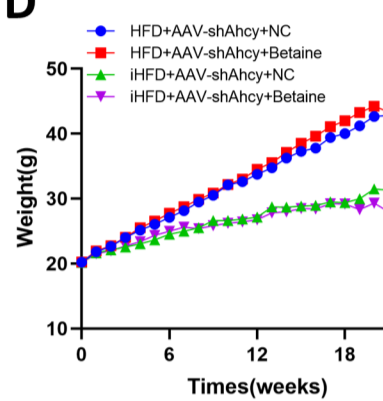**E**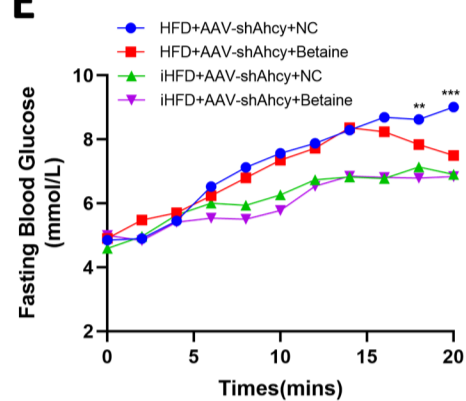**F**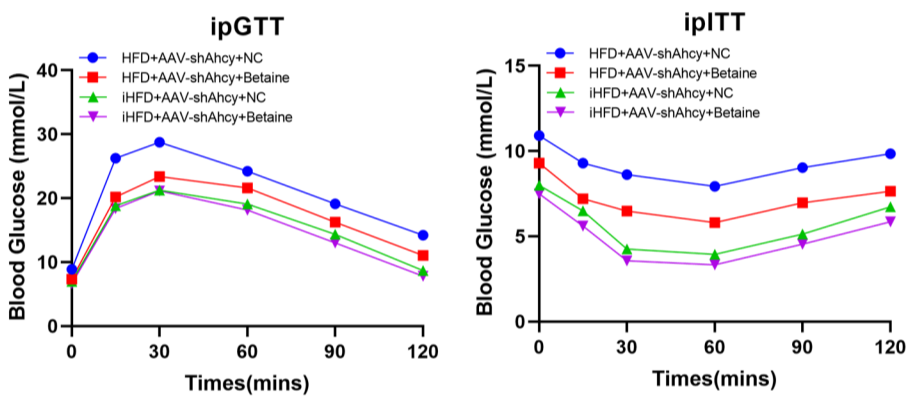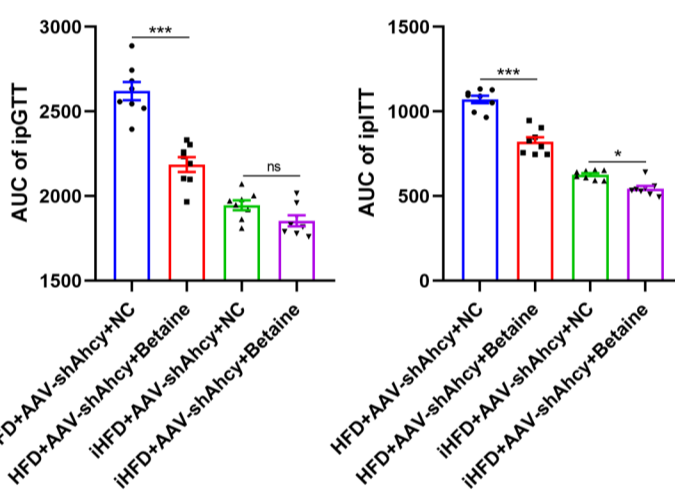**G**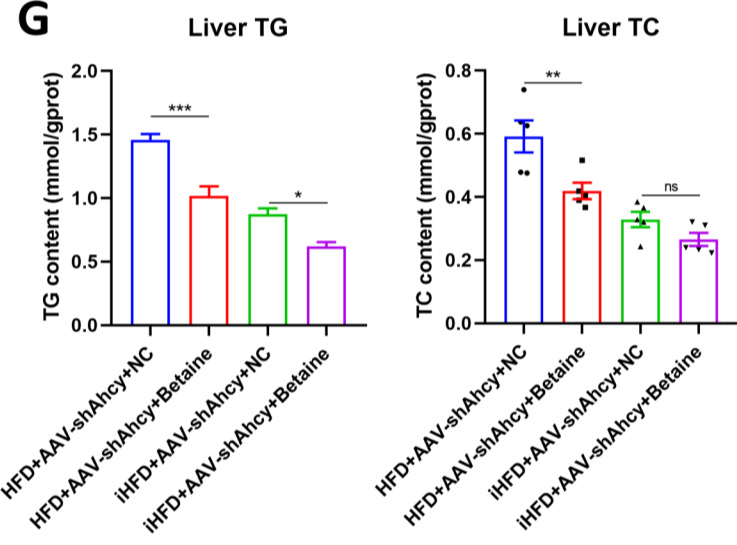**H**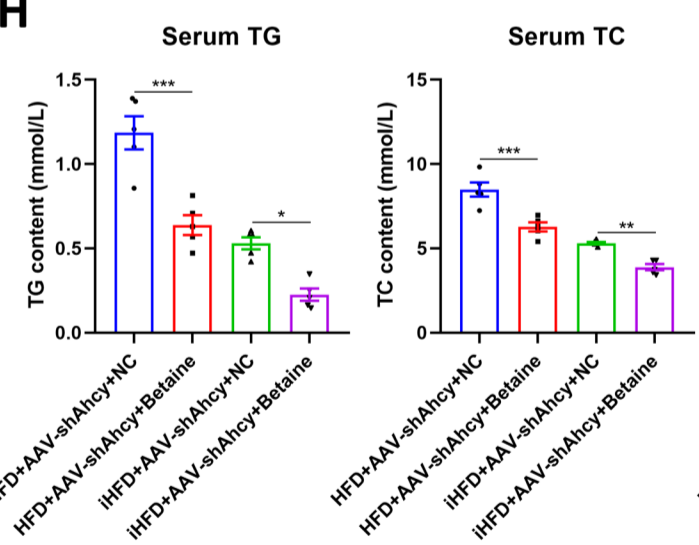**I**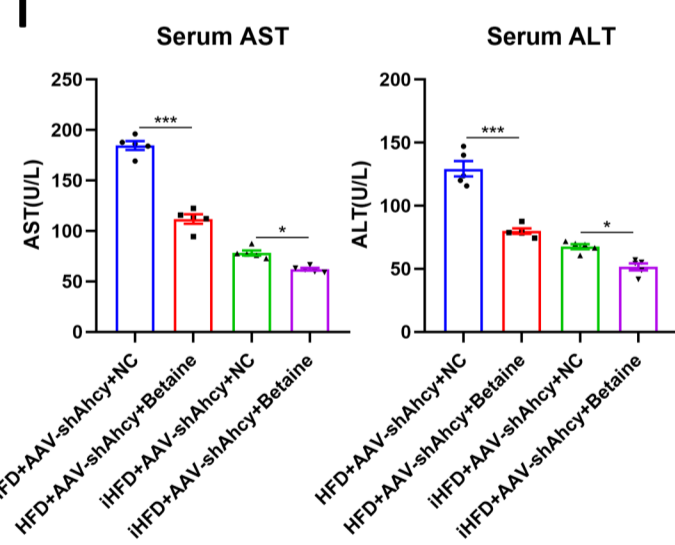**J**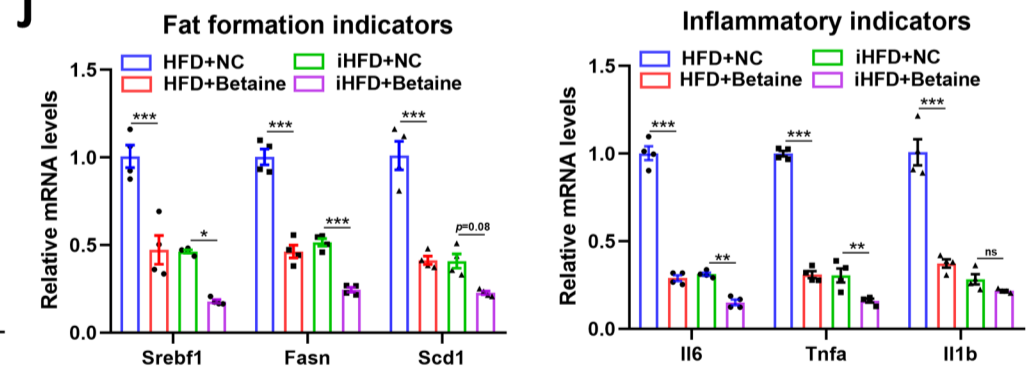**K**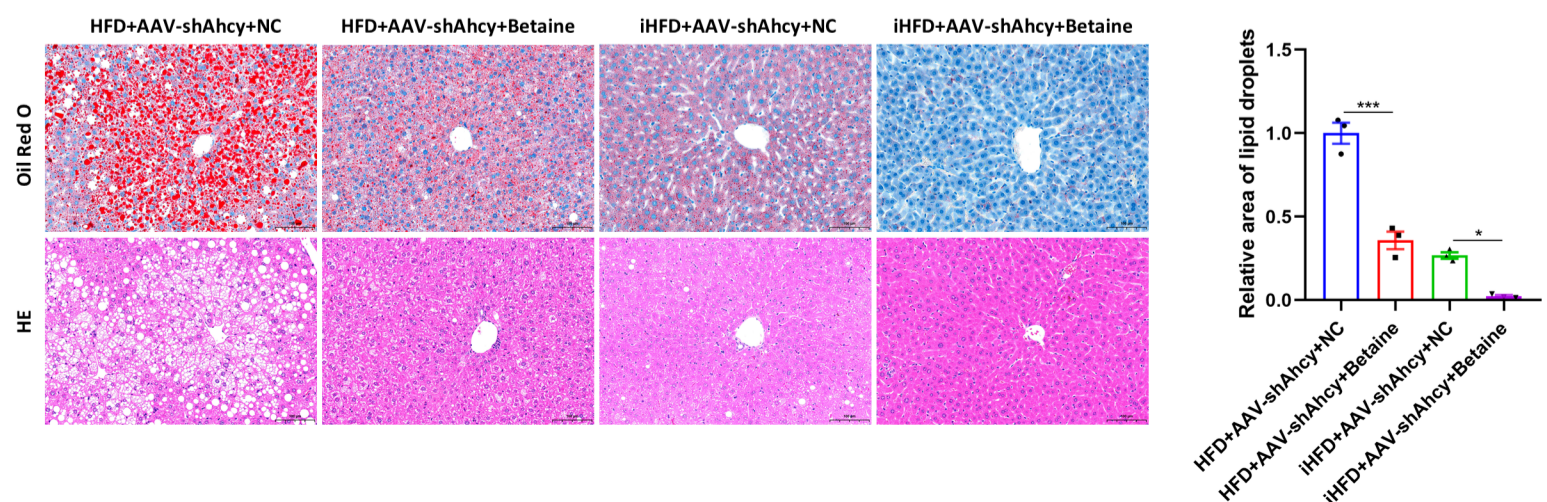

Supplement: Supplementary file 2 — Supporting File 2: advs76826‐sup‐0002‐FigureS1‐S9.zip. [file ADVS-9999-e76826-s001.zip › Supplementary Figure S8.PDF]

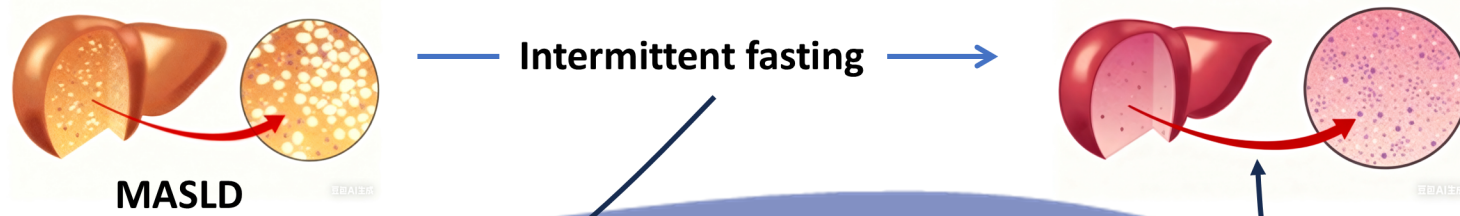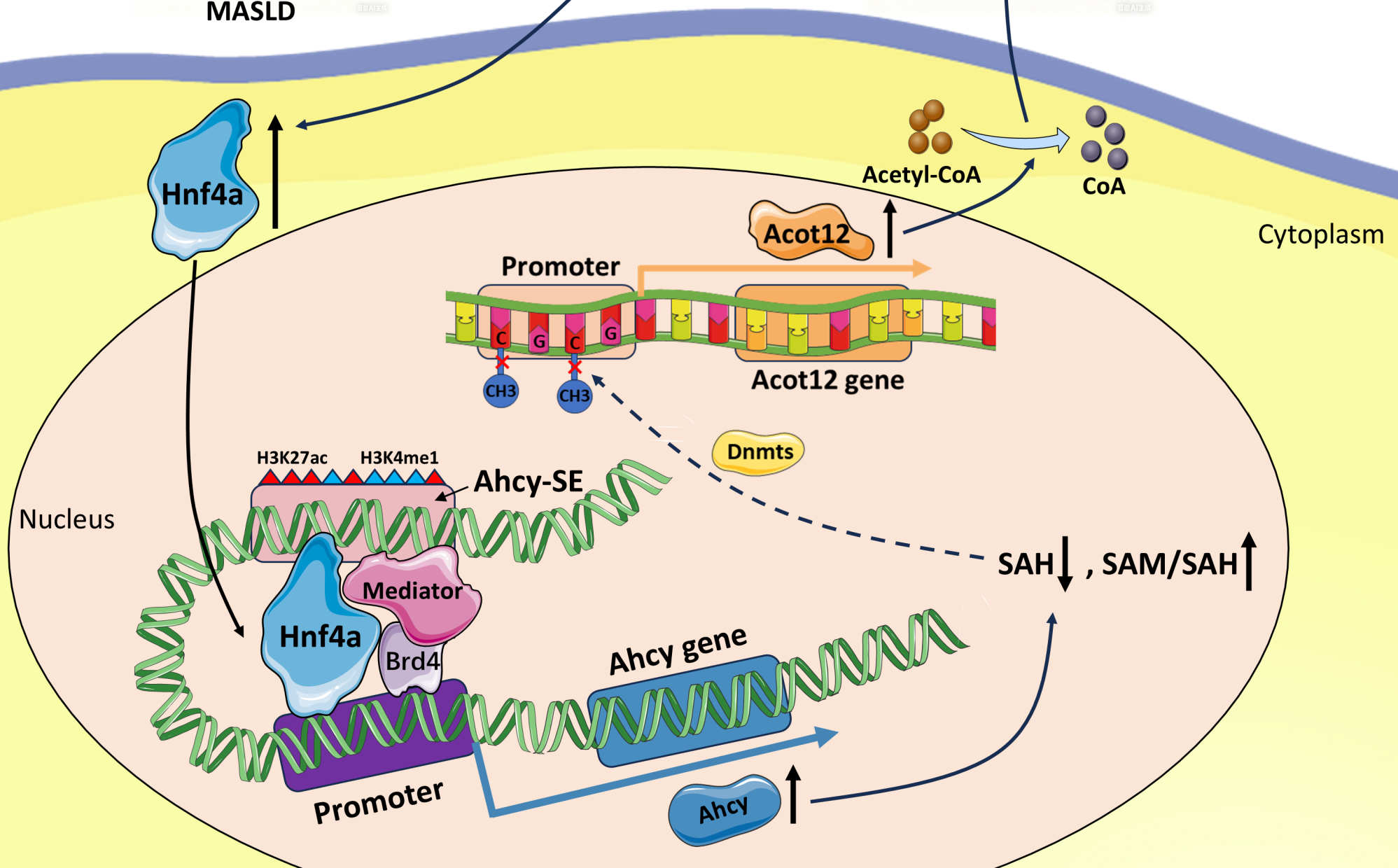

Supplement: Supplementary file 2 — Supporting File 2: advs76826‐sup‐0002‐FigureS1‐S9.zip. [file ADVS-9999-e76826-s001.zip › Supplementary Figure S9.PDF]
